# Design, Synthesis, and In Vitro and In Silico Study of New Hybrid 1-(2-(4-Arylthiazol-2-yl)hydrazineylidene)-5,6-dihydro-4*H*-pyrrolo[3,2,1-*ij*]quinolin-2-ones as Factor Xa and Factor XIa Inhibitors

**DOI:** 10.3390/molecules30173544

**Published:** 2025-08-29

**Authors:** Anna A. Skoptsova, Athina Geronikaki, Anthi Petrou, Nadezhda P. Novichikhina, Nadezhda A. Podoplelova, Georgii A. Bykov, Aleksandr A. Anis’kov, Svetlana A. Soloveva, Khidmet S. Shikhaliev

**Affiliations:** 1 Department of Organic Chemistry, Faculty of Chemistry, Voronezh State University, 1 Universitetskaya Sq., 394018 Voronezh, Russia; annk0611@mail.ru (A.A.S.); novichikhina@chem.vsu.ru (N.P.N.); 2 School of Pharmacy, Aristotle University of Thessaloniki, 54124 Thessaloniki, Greece; anthi.petrou.thessaloniki1@gmail.com; 3 Center for Theoretical Problems of Physicochemical Pharmakology, 119991 Moscow, Russia; podoplelova.nadezhda@ctppcp.ru (N.A.P.); bga20000@gmail.com (G.A.B.); 4 R&D Department, VIC Animal Health, VIC Group, 308570 Belgorod, Russia; aniskovalvis@gmail.com; 5 Nesmeyanov Institute of Organoelement Compounds, Russian Academy of Sciences, 119334 Moscow, Russia; aksenova.sa@phystech.edu

**Keywords:** anticoagulant, factor Xa, factor XIa, inhibitors, molecular docking, pyrrolo[3,2,1-ij]quinolin-2-one, thiazole

## Abstract

To develop efficient and structurally novel anticoagulants, a library of new hybrid molecules—(Z)-1-(2-(4-arylthiazol-2-yl)hydrazineylidene)-5,6-dihydro-4H-pyrrolo[3,2,1-*ij*]quinolin-2-ones—was designed and synthesized through a two-step approach. The reaction of pyrrolo[3,2,1-*ij*]quinoline-1,2-diones with thiosemicarbazide produced thiosemicarbazones, which were subsequently reacted with α-bromoacetophenones. The structure of the resulting compounds was determined by HPLC-HRMS-ESI analysis, ^1^H NMR spectroscopy, and ^13^C NMR spectroscopy. X-ray diffraction analysis unambiguously confirmed the structure of the resulting substances. The synthesized compounds were tested for their anticoagulant activity in vitro. Among the tested derivatives, two substances have a dual effect and exhibit 98–100% inhibitory ability against blood coagulation factors Xa and XIa at 30 μM. IC_50_ values were also evaluated for these compounds. The results obtained show the high potential of the synthesized derivatives in the development of new multitarget anticoagulant drugs. The docking studies confirmed the experimental results.

## 1. Introduction

Thromboembolic diseases are one of the leading causes of morbidity and mortality in the modern world [1]. At the same time, traditional antithrombosis therapy has a number of imperfections, which include a high risk of bleeding, a narrow therapeutic window, and undesirable interactions with drugs and food [2]. The limitations of available therapy have led to new approaches in the development of antithrombotic agents. One such approach is the development of anticoagulants that target specific serine proteases involved in the blood coagulation cascade. Small molecule inhibitors, attractive due to the possibility of oral administration, are currently widely studied as inhibitors of blood coagulation factors in clinical trials [3].

In particular, the attention of researchers has focused on small-molecule inhibitors affecting factor Xa [4]. Factor Xa is an enzyme that occupies a central position at the intersection of three pathways in the blood coagulation cascade. Inhibition of FXa is an important mechanism for suppressing coagulation. The effect on factor Xa is accompanied by an antithrombotic effect due to the reduction in thrombin formation, which reduces the activation of both coagulation and platelets without affecting the activity of existing thrombin. This is usually sufficient to ensure normal hemostasis [4,5]. Several small-molecule oral factor Xa inhibitors have already entered clinical practice or are in various stages of clinical trials, such as rivaroxaban, apixaban, edoxaban, betrixaban, and eribaxaban [4].

An enzyme belonging to the internal pathway of the blood coagulation cascade, factor XIa, has in recent years attracted increasing attention from researchers as a target protein. Developments in this area show that inhibition of factor XIa has a negligible effect on normal hemostasis [3,6,7]. Natural factor XI deficiency in people with hemophilia C has been found to not lead to serious bleeding disorders [8,9]. It is likely that its deficiency does not prevent thrombus formation but rather causes significant instability of the clot, which leads to the prevention of vascular obstruction [10,11]. Apixaban and milvexian, two oral small-molecule FXIa inhibitors, are currently undergoing phase 3 clinical trials to prevent stroke [3].

Despite the obvious achievements of modern medicine in the prevention of thrombosis and associated pathological diseases, the shortcomings of the drugs introduced into the market encourage researchers to discover new, highly effective, bioavailable anticoagulants with a better safety profile.

Clinical practice shows that dual antithrombotic therapy mediated by action on more than one target has beneficial effects [12]. In particular, the importance of co-inhibition of factors Xa and XIa to prevent increased thrombosis due to arterial injury in mice has been described in the literature [13]. Also, inhibitors acting on this combination of targets are found in systems of natural origin [14,15].

The concept of hybrid molecules, which has been actively developing over the past decades in the field of drug development, can become a convenient tool for the design of new multitarget anticoagulant drugs. This approach is used to obtain multifunctional structures capable of binding to several target proteins, which allows biological activity to be synergized and also reduces overall toxicity. It is worth noting that from a pharmaceutical point of view, hybrid molecules are more attractive in comparison with drugs based on individual substructures [16].

### Rational Design

**Figure 1:**
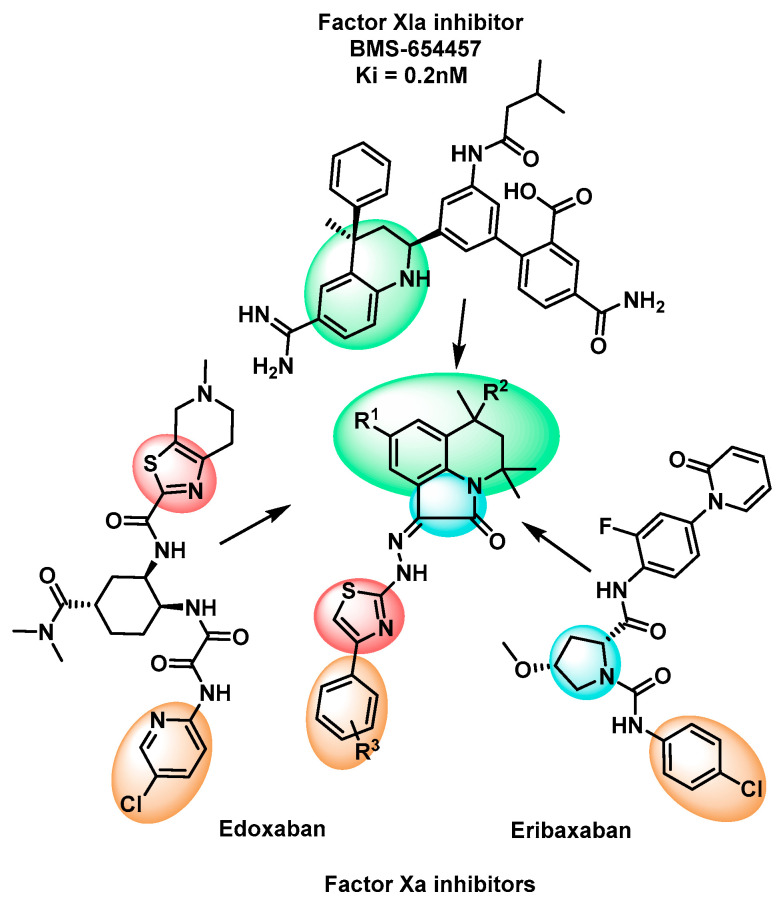
Design of target molecules. Factor XIa inhibitor BMS-654457 [17].

Herein, we report the design and synthesis of new hybrid molecules incorporating privileged quinoline, pyrrole, and thiazole fragments in their structure. These pharmacophores are presented in drugs with pronounced inhibitory activity against blood coagulation factors Xa and XIa [4,11,17]. Taking this into account, we assumed that our molecules probably will have an anticoagulant potential due to dual action, being inhibitors of factors Xa and XIa at the same time (Figure 1).

According to the X-ray crystal structure of a ligand with factor Xa or factor XIa, potential inhibitors should contain several pharmacophoric units—a central framework and chemical fragments capable of binding in specified pockets of the active center of the target protein. Pyrrolo[3,2,1-*ij*]quinoline-1,2-dione is a heterofunctional compound that has already proven to be a convenient matrix for introducing a variety of pharmacophore fragments as well as various substituents [18,19,20,21].

The basis of the factor Xa binding site consists of pockets S1 and S4, as well as surrounding residues. The lower part of the deep S1 pocket is covered by the negatively charged residue Asp189. The S4 binding site is an aromatic block formed by Tyr99, Phe174, and Trp215 [4]. The literature survey revealed that substituents containing various halogens as well as a methoxy substituent are P1 fragments that bind in the S1 pocket [2,4], while the S4 pocket is characterized by binding to various mono- and biaryl fragments of inhibitor molecules [2,4].

The active site of factor XIa has a single deep pocket, S1, with Asp189 at its base. XIa inhibitors are mainly fixed there, spreading to the immediate environment and usually additionally binding in the S1′ and S2′ pockets. The S2 pocket is bounded by Tyr58B, while the S2′ pocket contains residues Ile151 and Tyr143, where polar interactions can be observed [22]. There are numerous data in the literature regarding fragments that can act as P2′ fragments [22,23,24,25,26,27,28,29]. It has been shown that benzyl and phenyl groups often act as P1′ fragments [22,23,24,29]. It has been noted that the introduction of halogen atoms into the benzene ring increases its affinity as a P1 fragment [23,25,30,31,32]. Also, 6-chloro-3,4-dihydro-1*H*-quinolin-2-one can act as a P1 fragment [24,26,33].

Based on the above, as well as on the principles of a structure-based approach for the development of new effective inhibitors, we synthesized and studied new hybrid molecules—1-(2-(4-arylthiazol-2-yl)hydrazineylidene)-5,6-dihydro-4*H*-pyrrolo[3,2,1-*ij*]quinolin-2-ones—in which the pyrrolo[3,2,1-*ij*]quinolin-2-one core is connected to a thiazole moiety via a hydrazine linker. Furthermore, in order to prepare drug candidates with potentially better binding to the active site of the target proteins, we varied substituents in positions 6 and 8 of the pyrrolo[3,2,1-*ij*]quinolin-2-one backbone and in the phenyl substituent of the thiazole ring.

## 2. Results and Discussion

### 2.1. Chemistry

As a starting material, 4,4,6-trimethyl-5,6-dihydro-4*H*-pyrrolo[3,2,1-*ij*]quinoline-1,2-dione **1** with various substituents at 6 and 8 was chosen.

**Scheme 1:**
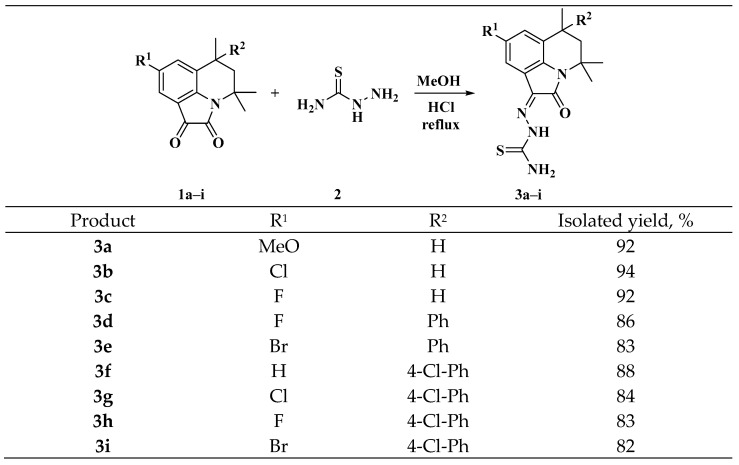
Preparation of 2-(4,4,6-trimethyl-2-oxo-5,6-dihydro-4*H*-pyrrolo[3,2,1-*ij*]quinolin-1(2*H*)-ylidene)hydrazine-1-carbothioamides **3a**–**i**.

A convenient two-step approach was used to obtain the target hybrid molecules **5a**–**o**. The presence of an active carbonyl group in position 2 of compound **1** makes it easy to carry out reactions with various nucleophilic agents [18,19,34,35]. Therefore, at the first stage, we synthesized intermediate thiosemicarbazones **3** through the reaction of pyrrolo[3,2,1-*ij*]quinoline-1,2-diones **1** with thiosemicarbazide **2** as an *N*-nucleophile [35]. Based on the literature data [34,35,36,37,38], we selected simple reaction conditions that allow isolation of the resulting thiosemicarbazones **3** in high yields (up to 94%). Thus, by boiling the starting substances **1a**–**i** and **2** in methanol followed by the addition of catalytic amounts of HCl, a series of 2-(4,4,6-trimethyl-2-oxo-5,6-dihydro-4*H*-pyrrolo[3,2,1-*ij*]quinolin-1(2*H*)-ylidene)hydrazine-1-carbothioamides **3a**–**i**, including the previously undescribed **3b**–**i**, were obtained (Scheme 1).

The structure of the obtained compounds was characterized by ^1^H and ^13^C spectroscopy and LCMS analysis. Thus, in the ^1^H NMR spectra, a set of signals characteristic of all structures of **3** is observed. The signals of the protons of the methyl groups at C^4^ and C^6^ are observed in the range of 0.69–1.70 ppm, while the signals of protons at C^5^ are at δ 1.54–1.87 ppm for **3b**,**c** and 2.10–2.56 ppm for **3d**–**i**. For compounds **3b**,**c**, the H^6^ signal is a multiplet at δ 2.79–3.01 ppm. Aromatic protons are observed in the region of 7.05–7.84 ppm in the form of singlets, doublets, and triplets depending on the corresponding substituents. Signals of two amino groups are detected at δ~8.7, 9.1 ppm and δ~12.4 ppm.

The presence of a double C=N bond, which is a structural feature of compounds **3a**–**i**, leads to the possible existence of the resulting thiosemicarbazones in the form of *E*/*Z* isomers.

According to LCMS analysis, as well as NMR spectroscopy, it was established that all the synthesized compounds are in the form of a single isomer. According to literary data [34,37,39] the Z-configuration is a more stable configuration for similar structures due to the possibility of forming an intramolecular hydrogen bond NH with the C=O fragment in position 2. Based on this, we assume that the thiosemicarbazones **3a**–**i** also exist in the form of *Z*-isomers.

**Scheme 2:**
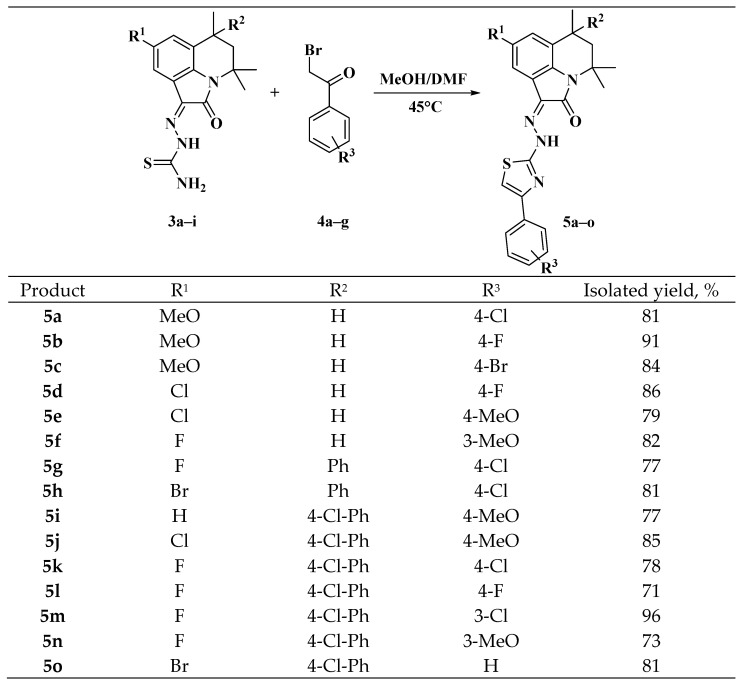
Synthesis of target compounds **5a**–**o**.

The resulting thiosemicarbazones **3**, due to the presence of a carbamothioylhydrazineylidene fragment in their structure, can be used to form a variety of *N*- and *S*-containing heterocycles [40,41]. In particular, the construction of a thiazole ring can be achieved through the reaction of thiosemicarbazones with α-halocarbonyl compounds according to the Hantzsch reaction [42]. Substituted α-bromoacetophenones **4a**–**g** as α-halogenketones were chosen in order to additionally introduce an aryl substituent into the structure of the molecule. A series of target 1-(2-(4-arylthiazol-2-yl)hydrazineylidene)-5,6-dihydro-4*H*-pyrrolo[3,2,1-*ij*]quinolin-2-ones **5a**–**o** was synthesized, as shown in Scheme 2, by the reaction of intermediates **3a**–**i** with various bromoacetophenones **4a**–**g** in the methanol/DMF system (*v*/*v* 5:1) in high yield following the literature [38].

The structure of the target molecules **5a**–**o** was characterized spectroscopically. Thus, in the ^1^H NMR spectra of **5a**–**o**, a number of additional signals are observed relative to the starting compounds **3a**–**i**: a singlet of the methine proton of the thiazole at δ~6.88–7.70 ppm and signals of the protons of the aryl substituent on the thiazole ring in the 6.91–7.95 ppm region. Furthermore, the disappearance of signals of NH_2_ group protons is detected.

**Scheme 3:**
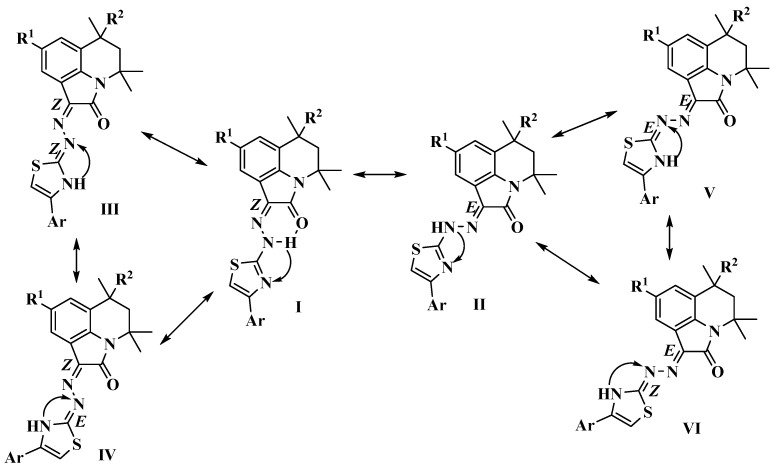
The structure of probable forms **I–VI** of compounds **5**.

Similar to the starting thiosemicarbazones **3a**–**i**, compounds **5a**–**o** also have the possibility of forming geometric isomers at the C=N bond. In addition, for similar structures of the isatin series, the possibility of prototropic tautomerism has been described in view of the possibility of proton migration between the nitrogen atoms of the thiazole ring and the hydrazone [36,43]. The structures of probable forms **I**–**VI** of compounds **5a**–**o** are shown in Scheme 3. It is noted that for forms **III**–**VI**, the formation of *E*/*Z* isomers is also possible at the exo-bond C=N of the thiazole ring. In accordance with the data of HPLC-MS analysis, as well as ^1^H and ^13^C NMR spectroscopy, the synthesized compounds represent one of the possible isomers, as evidenced by the lack of duplication of characteristic signals for all compounds **5a**–**o** in the ^1^H NMR and ^13^C NMR spectra.

**Figure 2:**
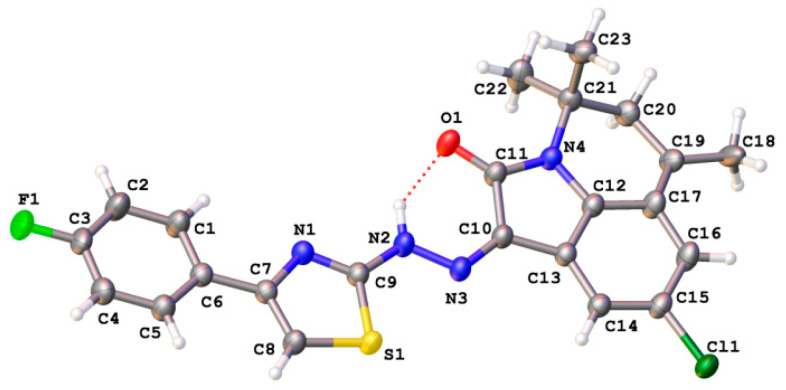
General view of **5d** with atoms shown as thermal ellipsoids at 50% probability level. The red dotted line indicates the hydrogen bond.

The structure of compounds **5a**–**o** was unambiguously confirmed by X-ray diffraction analysis of a single crystal of **5d** (Figure 2), which featured an intramolecular hydrogen bond between the NH group of hydrazone and the C=O fragment of pyrroloquinolinone (N…O 2.693(2) Å, NHO 137(3)°). The latter probably contributes to the additional stabilization of the *Z*-configuration of the molecule. Thus, compounds **5a**–**o** adopt the form **I** with an endo-C=N bond in the thiazole ring and a *Z*-configuration of the C=N bond at the pyrroloquinoline fragment. This stabilization favors the Z-configuration, maintaining an orientation of the pharmacophoric moieties to enable efficient insertion into the active sites of factors Xa and XIa. Such conformational adaptability, together with internal hydrogen-bond stabilization, may potentially enhance target affinity and could contribute to the potent inhibitory activity.

### 2.2. Anticoagulant Studies

All target hybrid molecules **5a**–**o** were screened for their in vitro FXa and FXIa inhibitory activity at 30 μM (Table 1). As a reference drug, the commercially available factor Xa inhibitor rivaroxaban was used. The best activity exhibited compounds **5d** and **5h** towards factors Xa and XIa with 98–100% inhibition. For further characterization of lead compounds **5d** and **5h**, their IC_50_ values were determined through measurements of the hydrolysis kinetics of specific substrates S2765 (for FXa) and S2366 (for FXIa) in buffer solution and in the presence of various concentrations of compounds (see Supplementary Materials, Figures S70–S73). Importantly, selectivity assessment of compounds **5d** and **5h** against plasmin—a key fibrinolytic serine protease—revealed minimal inhibition (<10% at 30 μM) for both **5d** and **5h** (Table 1), demonstrating their preferential activity against the target coagulation factors.

In addition, compound **5g** showed an average value of FXa inhibition and a good value of FXIa inhibition, while compound **5k** also demonstrated moderate activity against FXIa. The negative values obtained for compounds **5a**–**c**,**f**,**i** indicate a potential activation effect and require a separate detailed study to uncover the molecular mechanisms underlying the potentiation of serine proteases.

**Figure 3:**
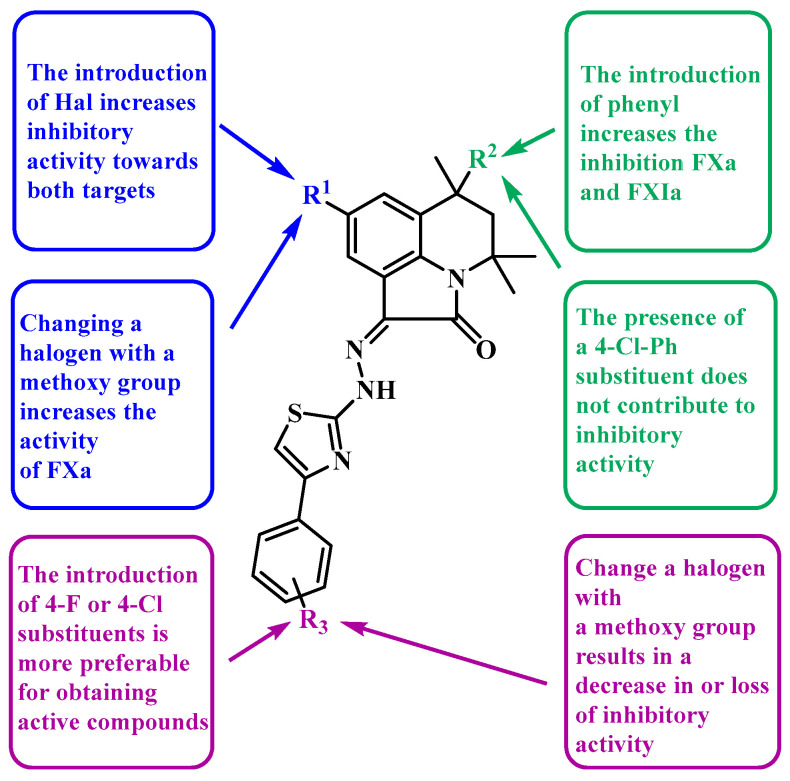
Structure–activity relationships.

Comparison of the obtained inhibition data allows us to suggest general patterns of structure–activity relationships for these compounds (Figure 3):

- The introduction of halogen substituents into position 8 of pyrrolo[3,2,1-*ij*]quinolin-2-one may contribute to the inhibition of both factors Xa and XIa (**5d**,**g**,**h**), thus increasing activity from −30% to 98% for Xa and from −6% to 99% for XIa.
- The presence of a methoxy group at position 8 of pyrrolo[3,2,1-*ij*]quinolin-2-one or in the phenyl substituent of the thiazole ring can result in a moderate potentiation of factor Xa activity by up to 40% but does not have a pronounced effect on factor XIa (**5a–c**,**f**,**i**).
- The introduction of F or Cl atoms into the para-position of the phenyl fragment in the thiazole ring is more preferable for the preparation of active compounds (**5d**,**g**,**h**,**k**) for XIa from −19% (**5n**) to 37% (**5k**) or from −8% and −2% (**5e**) to 98% and 99% (**5d**) for Xa and XIa, respectively.
- The appearance of a phenyl substituent in position 6 of pyrrolo[3,2,1-*ij*]quinoline-2-one increases the inhibitory ability (**5g**,**h**), thus increasing activity from −12% to 51% for Xa and from 37% to 76% for XIa.

### 2.3. Docking Studies

To identify the structural patterns essential for binding to the target protein, the geometries of protein–ligand complexes predicted by docking for the top inhibitors of factor Xa and factor XIa were analyzed (Table 2).

#### 2.3.1. Docking to Factor Xa

**Figure 4:**
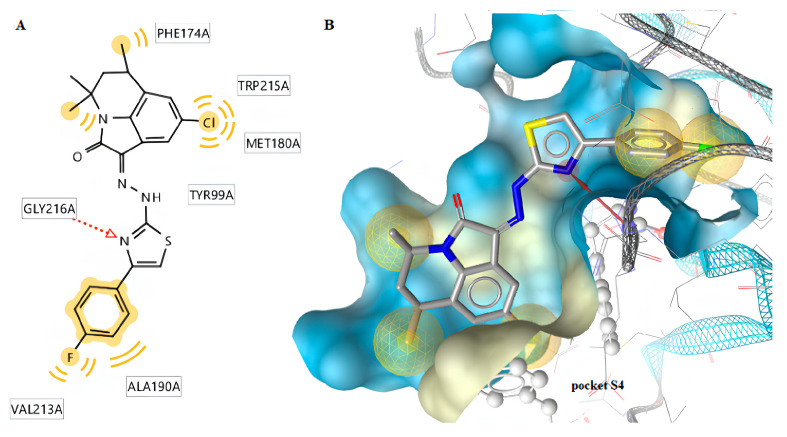
(**A**) A 2D diagram of the most active compound, **5d**, in the active site of factor Xa. (**B**) Docking pose of compound **5d** in the active site of factor Xa. Red dotted arrows indicate H-bonds, and yellow spheres indicate hydrophobic interactions.

The binding modes to factor Xa of the most active compounds, **5d** and **5h**, were predicted using docking studies. As shown in Figure 4, the central scaffold of compound **5d** is positioned over Gly-216, with the nitrogen atom of the thiazole ring oriented towards Gly-216, forming a hydrogen bond. This positioning promotes the compound’s binding within the S4 pocket. Additionally, the benzene ring may engage in π-stacking interactions with the aromatic ring of Tyr-99 (Figure 4). Furthermore, plenty of hydrophobic interactions with Phe174, Trp211, Met180, Ala190, and Val213 further contribute to the stability of the ligand–enzyme complex.

**Figure 5:**
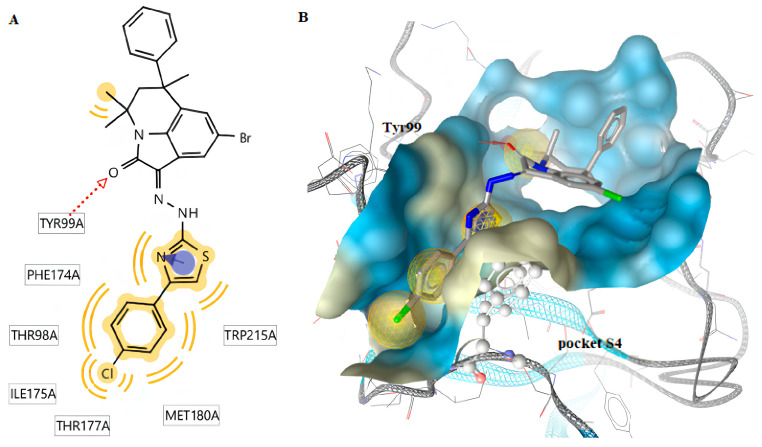
(**A**) A 2D diagram of the most active compound, **5h**, in the active site of factor Xa. (**B**) Docking pose of compound **5h** in the active site of factor Xa. Red dotted arrows indicate H-bonds, blue arrows indicate positive ionizable interactions, and yellow spheres indicate hydrophobic interactions.

Compound **5h** is located slightly closer to Tyr-99, potentially forming a hydrogen bond between its C=O oxygen atom and the phenolic hydroxyl group of Tyr-99 (Figure 5). Seven hydrophobic interactions of the molecule with amino acids Phe174, Thr98, Met175, Thr177, Met180, and Trp215 further stabilize the ligand–enzyme complex, showing lower free energy of binding compared to compound **5d**.

#### 2.3.2. Docking to Factor XIa

**Figure 6:**
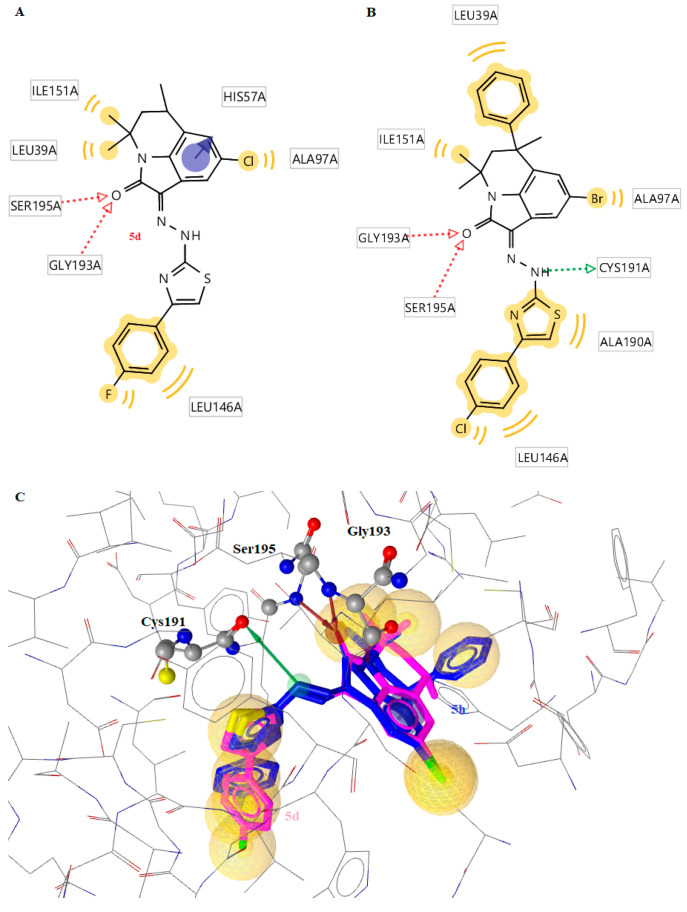
(**A**) A 2D diagram of the most active compound, **5d**, in factor XIa. (**B**) A 2D diagram of the most active compound, **5h**, in factor XIa. (**C**) Superposition of the most active compounds, **5d** (blue) and **5h** (pink), in the active center of factor XIa. Red and green dotted arrows indicate H-bonds, blue arrows indicate positive ionizable interactions, and yellow spheres indicate hydrophobic interactions.

In the case of factor XIa, the most active compounds, **5d** and **5h**, are located in the active site of the target protein in a similar way. The binding mode of structures **5d** and **5h** in the active site of factor XIa is shown in Figure 6. The docking poses of both compounds reveal that they bind in the same way, forming hydrogen bonds with residues Ser195 and Gly193. Moreover, compound **5h** forms an extra hydrogen bond with residue Cys191, in contrast to **5d**. Furthermore, plenty of hydrophobic interactions between the compound and Ala190 and Leu146, Ala97, Ile151, Leu39, and Leu146 further stabilize the ligand–enzyme complex. Compound **5h** forms an extra hydrogen bond with residue Cys191 as well as a hydrophobic interaction with Ala190, in contrast to **5d**, explaining its better free energy of binding.

### 2.4. Drug-Likeness

One of the key challenges in drug development is achieving efficient oral absorption, as it plays a crucial role in determining the therapeutic potential of a compound. Therefore, we predicted drug-likeness; the results for all compounds are summarized in Table 3.

In this study, compounds **5a**–**5f**, including the most active compound, **5d**, exhibited high gastrointestinal (GI) absorption, showing only one violation of Lipinski’s Rule of Five, with a bioavailability score of 0.55, which should suggest a high probability of efficient absorption. These compounds are also predicted to inhibit the cytochrome P450 enzyme CYP3A4 and act as substrates of P-glycoprotein (P-gp), which may influence their metabolic stability and efflux profiles.

In contrast, compounds **5g**–**5o** demonstrated low GI absorption and had two violations of Lipinski’s rule, indicating a reduced likelihood of favorable oral bioavailability. Moreover, toxicity predictions revealed the presence of pain-related alerts in all these compounds, with specific structural alerts identified, including imine and statin moieties. This alert surely requires attention in the next stages of study. Nevertheless, it has to be highlighted that several safe drugs in various therapeutic areas, including anti-inflammatory, anti-infective, anti-cancer, and antidiabetic areas, have been found to have the presence of pain-related alerts [44].

Overall, compounds **5a**–**5f** stand out, with better pharmacokinetic and drug-likeness characteristics, particularly compound **5d**, which emerges as a promising candidate for further lead optimization and development.

Moreover, we calculated the ADMET properties of the compounds using the pkCSM pharmacokinetics server at https://biosig.lab.uq.edu.au/pkcsm/prediction (accessed on 26 August 2025), and the results are presented in Table 4.

One of the most critical challenges for oral medications is their ability to cross the intestinal epithelial barrier, which controls the rate and extent of human absorption and, consequently, their bioavailability. The Caco-2 permeability assay was used to predict how well orally administered drugs will be absorbed; a value > 8 × 10^−6^ cm/s indicates high permeability. For the pkCSM predictive model, a value > 0.90 indicates high Caco2 permeability. All compounds have higher values than this threshold, indicating good permeability. The positive values of all compounds except the reference drug rivaroxaban show that they can be transported across the cell membrane by the ATP-binding cassette (ABC) transporter, a component of P-glycoprotein.

The volume of distribution (VDss) indicates the extent to which a drug is evenly distributed throughout the body. A VDss value below 0.15 log VDss (log L/kg) suggests low distribution, while values exceeding 0.45 log VDss indicate high distribution. In this context, all compounds are considered high VDss compounds except for the reference drug. The blood–brain barrier (BBB) permeability reflects a substance’s ability to enter the brain. A logBB value greater than 0.3 typically signifies BBB penetration. However, the logBB values for all compounds indicate low BBB permeability. Similarly, the majority of compounds exhibit low permeability to the central nervous system (CNS), which have logPS values below −2.0.

The metabolism prediction analysis suggests that all investigated compounds are likely to function both as substrates and inhibitors of the cytochrome P450 isoform CYP3A4. Regarding hepatotoxicity, all compounds—except for **5h**, **5o**, and the reference drug—are predicted to possess hepatotoxic potential, while the other three are classified as non-hepatotoxic. Furthermore, ADMET toxicity profiling indicates that compounds **5h**, **5k**, **5m**, and **5o**, along with the reference drug rivaroxaban, may present additional toxicity risks.

## 3. Materials and Methods

The purity of the starting materials and the synthesized compounds, as well as the analysis of reaction mixtures, was monitored by TLC on silica gel 60 F_254_ plates (Merck, Albany, GA, USA) using chloroform, methanol, or their mixtures as eluent. The purity of the obtained compounds was confirmed by HPLC analysis. HPLC analysis was performed using an Agilent 1260 Infinity liquid chromatograph (Agilent Technologies, Waldbronn, Germany) equipped with a UV detector in combination with an Agilent 6230 TOF LC/MS detector. The melting points (m.p.) of the resulting compounds were determined on a Stuart SMP30 instrument (Bibby Scientific Ltd., Stone, Staffordshire, UK). ^1^H NMR and ^13^C NMR spectra were recorded on an Agilent-400MR-vnmrs400 (400 and 101 MHz) spectrometer (Agilent Corp, Santa-Clara, CA, USA). The spectra were recorded at 20 °C employing CDCl_3_ or DMSO-*d*_6_ as solvents. Chemical shifts (δ) were expressed in ppm relative to TMS as the internal standard. For synthetic purposes, commercially available solvents and reagents (SigmaAldrich (St. Louis, MO, USA), Merck, and Acros Organics (Geel, Belgium)) were used.

### 3.1. Synthesis 

**General procedure for the synthesis of carbothioamide derivatives 3a**–**i.**

A mixture of 4,4,6-trimethyl-5,6-dihydro-4*H*-pyrrolo[3,2,1-*ij*]quinoline-1,2-dione **1a**–**i** (5 mmol) and thiosemicarbazide **2** (7.5 mmol) in methanol (20 mL) was refluxed for 5 min until the starting reagents were completely dissolved. HCl (1 mL) was added to the solution, and the resulting precipitate was filtered, washed with methanol (3 mL), and dried. The physicochemical and spectral data of (*Z*)-2-(8-methoxy-4,4,6-trimethyl-2-oxo-5,6-dihydro-4*H*-pyrrolo[3,2,1-*ij*]quinolin-1(2*H*)-ylidene)hydrazine-1-carbothioamide **3a** have been previously described [34].

*(Z)-2-(8-Chloro-4,4,6-trimethyl-2-oxo-5,6-dihydro-4H-pyrrolo[3,2,1-ij]quinolin-1(2H)-ylidene)hydrazine-1-carbothioamide* (**3b**). Yellow-orange solid; 1.58 g; yield 94%; m.p. 268–270 °C; ^1^H NMR (400 MHz, DMSO-*d*_6_), δ (ppm): 1.29 (3H, d, *J* = 6.7 Hz, C^6^-CH_3_), 1.32 (3H, s, C^4^-CH_3_), 1.54 (1H, t, *J* = 12.9 Hz, C^5^-H), 1.69 (3H, s, C^4^-CH_3_), 1.86 (1H, dd, *J* = 13.7 Hz, *J* = 4.5 Hz, C^5^-H), 2.83–2.96 (1H, m, C^6^-H), 7.32 (1H, s, Ar–H), 7.52 (1H, s, Ar–H), 8.74 (1H, s, NH_2_), 9.10 (1H, s, NH_2_), 12.39 (1H, s, NH).^13^C NMR (101 MHz, DMSO-*d*_6_), δ (ppm): 18.2, 24.9, 26.0, 26.9, 45.2, 54.9, 118.5, 119.9, 127.3, 127.5, 128.2, 131.0, 138.5, 160.4, 179.2. HPLC-HRMS-ESI, *m*/*z*([M + H]^+^), calcd for C_15_H_17_ClN_4_OS + H^+^ 337.0885, found 337.0882.

*(Z)-2-(8-Fluoro-4,4,6-trimethyl-2-oxo-5,6-dihydro-4H-pyrrolo[3,2,1-ij]quinolin-1(2H)-ylidene)hydrazine-1-carbothioamide* (**3c**). Yellow-orange solid; 1.45 g; yield 92%; m.p. 262–264 °C; ^1^H NMR (400 MHz, DMSO-*d*_6_), δ (ppm): 1.19–1.41 (6H, m, C^6^-CH_3_ + C^4^-CH_3_), 1.54 (1H, t, *J* = 12.9 Hz, C^5^-H), 1.70 (3H, s, C^4^-CH_3_), 1.87 (1H, dd, *J* = 13.6 Hz, *J* = 4.0 Hz, C^5^-H), 2.79–3.01 (1H, m, C^6^-H), 7.16 (1H, d, *J* = 10.3 Hz, Ar–H), 7.27 (1H, d, *J* = 7.3 Hz, Ar–H), 8.68 (1H, s, NH_2_), 9.09 (1H, s, NH_2_), 12.46 (1H, s, NH).^13^C NMR (101 MHz, DMSO-*d*_6_), δ (ppm): 18.3, 24.8, 26.1, 26.9, 45.3, 54.7, 105.7, 105.9, 114.4, 114.7, 119.3, 119.4, 128.1, 128.2, 131.6, 131.6, 136.0, 158.2, 160.5, 179.2. HPLC-HRMS-ESI, *m*/*z*([M + H]^+^), calcd for C_15_H_17_FN_4_OS + H^+^ 321.1181, found 321.1185.

*(Z)-2-(8-Fluoro-4,4,6-trimethyl-2-oxo-6-phenyl-5,6-dihydro-4H-pyrrolo[3,2,1-ij]quinolin-1(2H)-ylidene)hydrazine-1-carbothioamide* (**3d**). Orange solid; 1.67 g; yield 86%; m.p. 264–266 °C; ^1^H NMR (400 MHz, DMSO-*d*_6_), δ (ppm): 0.69 (3H, s, C^4^-CH_3_), 1.61 (3H, s, C^4^-CH_3_), 1.67 (3H, s, C^6^-CH_3_), 2.11 (1H, d, *J* = 14.4 Hz, C^5^-H), 2.42–2.55 (1H, m, C^5^-H), 7.06 (2H, d, *J* = 7.8 Hz, Ar–H), 7.16 (1H, t, *J* = 7.0 Hz, Ar–H), 7.24 (2H, t, *J* = 7.6 Hz, Ar–H), 7.29 (1H, dd, *J* = 10.5 Hz, *J* = 2.4 Hz, Ar–H), 7.46 (1H, dd, *J* = 7.8 Hz, *J* = 2.3 Hz, Ar–H), 8.76 (1H, s, NH_2_), 9.14 (1H, s, NH_2_), 12.43 (1H, s, NH). ^13^C NMR (101 MHz, DMSO-*d*_6_), δ (ppm): 25.2, 28.1, 30.7, 40.3, 51.1, 54.7, 106.6, 106.9, 116.0, 116.2, 120.1, 120.2, 126.7, 126.9, 128.7, 128.8, 131.4, 131.5, 136.1, 147.7, 158.1, 160.4, 179.2. HPLC-HRMS-ESI, *m*/*z*([M + H]^+^), calcd for C_21_H_21_FN_4_OS + H^+^ 397.1494, found 397.1491.

*(Z)-2-(8-Bromo-4,4,6-trimethyl-2-oxo-6-phenyl-5,6-dihydro-4H-pyrrolo[3,2,1-ij]quinolin-1(2H)-ylidene)hydrazine-1-carbothioamide* (**3e**). Yellow-orange solid; 1.86 g; yield 83%; m.p. 259–261 °C; ^1^H NMR (400 MHz, DMSO-*d*_6_), δ (ppm): 0.71 (3H, s, C^4^-CH_3_), 1.61 (3H, s, C^4^-CH_3_), 1.68 (3H, s, C^6^-CH_3_), 2.10 (1H, d, *J* = 14.4 Hz, C^5^-H), 2.42–2.53 (1H, m, C^5^-H), 7.05 (2H, d, *J* = 7.8 Hz, Ar–H), 7.16 (1H, t, *J* = 7.2 Hz, Ar–H), 7.25 (2H, t, *J* = 7.6 Hz, Ar–H), 7.54 (1H, s, Ar–H), 7.84 (1H, s, Ar–H), 8.81 (1H, s, NH_2_), 9.12 (1H, s, NH_2_), 12.35 (1H, s, NH).^13^C NMR (101 MHz, DMSO-*d*_6_), δ (ppm): 25.3, 28.1, 30.5, 40.3, 51.2, 54.8, 115.3, 121.0, 122.1, 126.7, 126.9, 128.7, 129.4, 130.8, 131.5, 138.9, 147.7, 160.2, 179.2. HPLC-HRMS-ESI, *m*/*z*([M + H]^+^), calcd for C_21_H_21_BrN_4_OS + H^+^ 457.0693, found 457.0691.

*(Z)-2-(6-(4-Chlorophenyl)-4,4,6-trimethyl-2-oxo-5,6-dihydro-4H-pyrrolo[3,2,1-ij]quinolin-1(2H)-ylidene)hydrazine-1-carbothioamide* (**3f**). Yellow-orange solid; 1.78 g; yield 88%; m.p. 246–248 °C; ^1^H NMR (400 MHz, DMSO-*d*_6_), δ (ppm): 0.75 (3H, s, C^4^-CH_3_), 1.63 (3H, s, C^4^-CH_3_), 1.66 (3H, s, C^6^-CH_3_), 2.13 (1H, d, *J* = 14.4 Hz, C^5^-H), 2.40–2.55 (1H, m, C^5^-H), 7.08 (2H, d, *J* = 8.5 Hz, Ar–H), 7.19 (1H, t, *J* = 7.6 Hz, Ar–H), 7.29 (2H, d, *J* = 8.4 Hz, Ar–H), 7.39 (1H, d, *J* = 7.9 Hz, Ar–H), 7.62 (1H, d, *J* = 7.4 Hz, Ar–H), 8.69 (1H, s, NH_2_), 9.06 (1H, s, NH_2_), 12.52 (1H, s, NH). ^13^C NMR (101 MHz, DMSO-*d*_6_), δ (ppm): 25.5, 28.1, 30.8, 39.7, 51.0, 54.6, 118.9, 119.7, 123.3, 126.5, 128.5, 129.0, 129.7, 131.3, 132.2, 139.7, 147.3, 160.6, 179.1. HPLC-HRMS-ESI, *m*/*z*([M + H]^+^), calcd for C_21_H_21_ClN_4_OS + H^+^ 413.1198, found 413.1196.

*(Z)-2-(8-Chloro-6-(4-chlorophenyl)-4,4,6-trimethyl-2-oxo-5,6-dihydro-4H-pyrrolo[3,2,1-ij]quinolin-1(2H)-ylidene)hydrazine-1-carbothioamide* (**3g**). Orange solid; 1.84 g; yield 84%; m.p. 243–245 °C; ^1^H NMR (400 MHz, DMSO-*d*_6_), δ (ppm): 0.74 (3H, s, C^4^-CH_3_), 1.61 (3H, s, C^4^-CH_3_), 1.67 (3H, s, C^6^-CH_3_), 2.11 (1H, d, *J* = 14.5 Hz, C^5^-H), 2.38–2.56 (1H, m, C^5^-H), 7.09 (2H, d, *J* = 8.5 Hz, Ar–H), 7.31 (2H, d, *J* = 8.5 Hz, Ar–H), 7.44 (1H, d, *J* = 1.6 Hz, Ar–H), 7.71 (1H, d, *J* = 1.6 Hz, Ar–H), 8.81 (1H, s, NH_2_), 9.13 (1H, s, NH_2_), 12.35 (1H, s, NH). ^13^C NMR (101 MHz, DMSO-*d*_6_), δ (ppm): 25.4, 28.0, 30.5, 40.0, 50.9, 54.8, 119.5, 120.8, 127.7, 128.5, 128.6, 128.8, 129.0, 130.9, 131.4, 138.5, 146.7, 160.3, 179.2. HPLC-HRMS-ESI, *m*/*z*([M + H]^+^), calcd for C_21_H_20_Cl_2_N_4_OS + H^+^ 447.0809, found 447.0804.

*(Z)-2-(6-(4-Chlorophenyl)-8-fluoro-4,4,6-trimethyl-2-oxo-5,6-dihydro-4H-pyrrolo[3,2,1-ij]quinolin-1(2H)-ylidene)hydrazine-1-carbothioamide* (**3h**). Orange solid; 1.75 g; yield 83%; m.p. 219–221 °C; ^1^H NMR (400 MHz, DMSO-*d*_6_), δ (ppm): 0.73 (3H, s, C^4^-CH_3_), 1.62 (3H, s, C^4^-CH_3_), 1.66 (3H, s, C^6^-CH_3_), 2.11 (1H, d, *J* = 14.5 Hz, C^5^-H), 2.43–2.54 (1H, m, C^5^-H), 7.10 (2H, d, *J* = 8.5 Hz, Ar–H), 7.25–7.34 (3H, m, Ar–H), 7.47 (1H, dd, *J* = 7.9 Hz, *J* = 2.3 Hz, Ar–H), 8.76 (1H, s, NH_2_), 9.13 (1H, s, NH_2_), 12.41 (1H, s, NH). ^13^C NMR (101 MHz, DMSO-*d*_6_), δ (ppm): 25.4, 28.01, 30.6, 40.0, 51.0, 54.7, 106.8, 107.1, 115.9, 116.2, 120.2, 120.3, 128.2, 128.2, 128.6, 129.0, 131.4, 136.0, 146.8, 158.1, 160.5, 179.2. HPLC-HRMS-ESI, *m*/*z*([M + H]^+^), calcd for C_21_H_20_ClFN_4_OS + H^+^ 431.1104, found 431.1108.

*(Z)-2-(8-Bromo-6-(4-chlorophenyl)-4,4,6-trimethyl-2-oxo-5,6-dihydro-4H-pyrrolo[3,2,1-ij]quinolin-1(2H)-ylidene)hydrazine-1-carbothioamide* (**3i**). Yellow-orange solid; 1.98 g; yield 82%; m.p. 242–244 °C; ^1^H NMR (400 MHz, DMSO-*d*_6_), δ (ppm): 0.74 (3H, s, C^4^-CH_3_), 1.60 (3H, s, C^4^-CH_3_), 1.67 (3H, s, C^6^-CH_3_), 2.10 (1H, d, *J* = 14.5 Hz, C^5^-H), 2.39–2.53 (1H, m, C^5^-H), 7.08 (2H, d, *J* = 8.5 Hz, Ar–H), 7.31 (2H, d, *J* = 8.5 Hz, Ar–H), 7.55 (1H, d, *J* = 1.5 Hz, Ar–H), 7.84 (1H, d, *J* = 1.4 Hz, Ar–H), 8.82 (1H, s, NH_2_), 9.13 (1H, s, NH_2_), 12.33 (1H, s, NH). ^13^C NMR (101 MHz, DMSO-*d*_6_), δ (ppm): 25.4, 28.0, 30.4, 40.0, 50.9, 54.7, 115.4, 121.1, 122.3, 128.6, 128.9, 129.0, 130.7, 131.4, 138.8, 146.8, 155.0, 160.2, 179.2. HPLC-HRMS-ESI, *m*/*z*([M + H]^+^), calcd for C_21_H_20_BrClN_4_OS + H^+^ 491.0303, found 491.0308.

**General procedure for the synthesis of (*Z*)-1-(2-(4-arylthiazol-2-yl)hydrazineylidene)-4,4,6-trimethyl-5,6-dihydro-4*H*-pyrrolo[3,2,1-*ij*]quinolin-2(1*H*)-ones 5a–o.**

A mixture of thiosemicarbazones **3a**–**i** and the corresponding α-bromoacetophenone **4** was dissolved in a methanol/DMF system (*v*/*v* 5:1). The reaction mixture was stirred at 45 °C for 1–2 h until a precipitate formed. The reaction progress was monitored using TLC (eluent: chloroform/methanol (10:1)). The resulting precipitate was filtered, washed with methanol (3 mL) and then water (10mL), and dried. If necessary, the resulting precipitate was recrystallized from DMF.

*(Z)-1-(2-(4-(4-Chlorophenyl)thiazol-2-yl)hydrazineylidene)-8-methoxy-4,4,6-trimethyl-5,6-dihydro-4H-pyrrolo[3,2,1-ij]quinolin-2(1H)-one* (**5a**). Orange solid; 0.38 g; yield 81%; m.p. 211–213 °C; ^1^H NMR (400 MHz, DMSO-*d*_6_), δ (ppm): 1.30 (3H, d, *J* = 6.5 Hz, C^6^-CH_3_), 1.35 (3H, s, C^4^-CH_3_), 1.58 (1H, t, *J* = 12.8 Hz, C^5^-H), 1.75 (3H, s, C^4^-CH_3_), 1.85 (1H, dd, *J* = 13.7 Hz, *J* = 4.4 Hz, C^5^-H), 2.83–2.96 (1H, m, C^6^-H), 3.76 (3H, s, MeO), 6.80 (1H, s, Ar–H), 6.89 (1H, s, Ar–H), 7.38 (2H, d, *J* = 8.4 Hz, Ar–H), 7.51 (1H, s, C_thiazole_-H), 7.85 (2H, d, *J* = 8.5 Hz, Ar–H), 13.49 (1H, s, NH). ^13^C NMR (101 MHz, DMSO-*d*_6_), δ (ppm): 18.4, 24.8, 26.1, 27.1, 45.9, 54.5, 56.1, 103.0, 107.5, 113.7, 118.5, 127.1, 127.7, 128.9, 132.6, 132.7, 132.9, 133.2, 150.3, 156.4, 161.2, 166.5. HPLC-HRMS-ESI, *m*/*z*([M + H]^+^), calcd for C_24_H_23_ClN_4_O_2_S + H^+^ 467.1304, found 467.1308.

*(Z)-1-(2-(4-(4-Fluorophenyl)thiazol-2-yl)hydrazineylidene)-8-methoxy-4,4,6-trimethyl-5,6-dihydro-4H-pyrrolo[3,2,1-ij]quinolin-2(1H)-one* (**5b**). Orange solid; 0.41 g; yield 91%; m.p. 204–206 °C; ^1^H NMR (400 MHz, DMSO-*d*_6_), δ (ppm): 1.31 (3H, d, *J* = 6.5 Hz, C^6^-CH_3_), 1.35 (3H, s, C^4^-CH_3_), 1.53–1.63 (1H, m, C^5^-H), 1.74 (3H, s, C^4^-CH_3_), 1.89 (1H, dd, *J* = 13.8 Hz, *J* = 4.5 Hz, C^5^-H), 2.82–2.97 (1H, m, C^6^-H), 3.78 (3H, s, MeO), 6.88 (1H, d, *J* = 2.2 Hz, Ar–H), 6.93 (1H, d, *J* = 2.2 Hz, Ar–H), 7.25 (2H, t, *J* = 8.8 Hz, Ar–H), 7.60 (1H, s, C_thiazole_-H), 7.92 (dd, 2H, *J* = 8.5 Hz, *J* = 5.7 Hz, Ar–H), 13.50 (1H, s, NH). ^13^C NMR (101 MHz, DMSO-*d*_6_), δ (ppm): 18.5, 24.9, 26.1, 27.1, 45.6, 54.6, 56.3, 103.0, 107.1, 114.0, 115.9, 116.1, 118.5, 127.5, 128.2, 128.2, 131.0, 132.7, 132.8, 150.5, 154.9, 156.5, 161.1, 161.2, 163.5, 166.5. HPLC-HRMS-ESI, *m*/*z*([M + H]^+^), calcd for C_24_H_23_FN_4_O_2_S + H^+^ 451.1600, found 451.1604.

*(Z)-1-(2-(4-(4-Bromophenyl)thiazol-2-yl)hydrazineylidene)-8-methoxy-4,4,6-trimethyl-5,6-dihydro-4H-pyrrolo[3,2,1-ij]quinolin-2(1H)-one* (**5c**). Orange solid; 0.43 g; yield 84%; m.p. 207–209 °C; ^1^H NMR (400 MHz, DMSO-*d*_6_), δ (ppm): 1.30 (3H, d, *J* = 6.5 Hz, C^6^-CH_3_), 1.35 (3H, s, C^4^-CH_3_), 1.53–1.63 (1H, m, C^5^-H), 1.74 (3H, s, C^4^-CH_3_), 1.88 (1H, dd, *J* = 14.0 Hz, *J* = 4.4 Hz, C^5^-H), 2.83–2.97 (1H, m, C^6^-H), 3.78 (3H, s, MeO), 6.88 (1H, d, *J* = 1.4 Hz, Ar–H), 6.93 (1H, d, *J* = 2.2 Hz, Ar–H), 7.60 (2H, d, *J* = 8.5 Hz, Ar–H), 7.69 (1H, s, C_thiazole_-H), 7.84 (2H, d, *J* = 8.5 Hz, Ar–H), 13.51 (1H, s, NH). ^13^C NMR (101 MHz, DMSO-*d*_6_), δ (ppm): 18.4, 24.8, 26.1, 27.1, 45.6, 54.6, 56.2, 103.0, 108.2, 114.0, 118.5, 121.5, 127.5, 128.1, 132.1, 132.7, 132.9, 133.6, 141.4, 150.3, 156.4, 161.2. HPLC-HRMS-ESI, *m*/*z*([M + H]^+^), calcd for C_24_H_23_BrN_4_O_2_S + H^+^ 511.0799, found 511.0794.

*(Z)-8-Chloro-1-(2-(4-(4-fluorophenyl)thiazol-2-yl)hydrazineylidene)-4,4,6-trimethyl-5,6-dihydro-4H-pyrrolo[3,2,1-ij]quinolin-2(1H)-one* (**5d**). Orange solid; 0.39 g; yield 86%; m.p. 244–246 °C; ^1^H NMR (400 MHz, DMSO-*d*_6_), δ (ppm): 1.32 (3H, d, *J* = 6.8 Hz, C^6^-CH_3_), 1.36 (3H, s, C^4^-CH_3_), 1.55–1.63 (1H, m, C^5^-H), 1.75 (3H, s, C^4^-CH_3_), 1.91 (1H, dd, *J* = 13.6 Hz, *J* = 4.4 Hz, C^5^-H), 2.89–2.99 (1H, m, C^6^-H), 7.25 (2H, t, *J* = 8.8 Hz, Ar–H), 7.35 (1H, s, Ar–H), 7.38 (1H, s, Ar–H), 7.64 (1H, s, C_thiazole_-H), 7.93 (2H, dd, *J* = 8.6 Hz, *J* = 5.7 Hz, Ar–H), 13.41 (1H, s, NH). ^13^C NMR (101 MHz, DMSO-*d*_6_), δ (ppm): 18.3, 25.0, 26.1, 27.0, 45.3, 54.9, 107.6, 115.9, 116.2, 117.3, 119.5, 126.8, 127.6, 128.2, 128.3, 128.3, 130.2, 130.3, 131.0, 131.4, 137.4, 150.5, 152.6, 161.1. HPLC-HRMS-ESI, *m*/*z*([M + H]^+^), calcd for C_23_H_20_ClFN_4_OS + H^+^ 455.1104, found 455.1106.

*(Z)-8-Chloro-1-(2-(4-(4-methoxyphenyl)thiazol-2-yl)hydrazineylidene)-4,4,6-trimethyl-5,6-dihydro-4H-pyrrolo[3,2,1-ij]quinolin-2(1H)-one* (**5e**). Orange solid; 0.37 g; yield 79%; m.p. 230–232 °C; ^1^H NMR (400 MHz, CDCl_3_), δ (ppm): 1.37 (3H, d, *J* = 6.7 Hz, C^6^-CH_3_), 1.43 (3H, s, C^4^-CH_3_), 1.66 (1H, t, C^5^-H), 1.78–1.88 (4H, m, C^4^-CH_3_+ C^5^-H), 2.89–2.99 (1H, m, C^6^-H), 3.84 (3H, s, MeO), 6.88 (1H, s, C_thiazole_-H), 6.93 (2H, d, *J* = 8.7 Hz, Ar–H), 7.16 (1H, s, Ar–H), 7.43 (1H, s, Ar–H), 7.76 (2H, d, *J* = 8.7 Hz, Ar–H), 13.47 (1H, s, NH). ^13^C NMR (101 MHz, CDCl_3_), δ (ppm): 18.2, 25.0, 26.1, 27.1, 46.1, 54.7, 55.4, 103.7, 114.0, 117.8, 117.9, 119.7, 126.0, 126.9, 127.2, 127.3, 128.2, 130.7, 136.8, 151.7, 159.5, 161.2, 166.4. HPLC-HRMS-ESI, *m*/*z*([M + H]^+^), calcd for C_24_H_23_ClN_4_O_2_S + H^+^ 467.1304, found 467.1303.

*(Z)-8-Fluoro-1-(2-(4-(3-methoxyphenyl)thiazol-2-yl)hydrazineylidene)-4,4,6-trimethyl-5,6-dihydro-4H-pyrrolo[3,2,1-ij]quinolin-2(1H)-one* (**5f**). Orange solid; 0.37 g; yield 82%; m.p. 209–211 °C; ^1^H NMR (400 MHz, CDCl_3_), δ (ppm): 1.36 (3H, d, *J* = 6.8 Hz, C^6^-CH_3_), 1.44 (3H, s, C^4^-CH_3_), 1.67 (1H, t, C^5^-H), 1.79–1.90 (4H, m, C^4^-CH_3_+ C^5^-H), 2.89–3.00 (1H, m, C^6^-H), 3.88 (3H, s, MeO), 6.87 (1H, dd, *J* = 8.1 Hz, *J* = 2.5 Hz, Ar–H), 6.92 (1H, d, *J* = 10.1 Hz, Ar–H), 7.03 (1H, s, C_thiazole_-H), 7.17 (1H, dd, *J* = 7.9 Hz, *J* = 2.3 Hz, Ar–H), 7.31 (1H, t, *J* = 7.9 Hz, Ar–H), 7.40 (1H, d, *J* = 7.7 Hz, Ar–H), 7.43 (1H, s, Ar–H), 13.52 (1H, s, NH). ^13^C NMR (101 MHz, CDCl_3_), δ (ppm): 18.2, 24.9, 26.2, 27.1, 46.1, 54.6, 55.3, 105.0, 105.2, 105.8, 111.0, 112.8, 113.1, 114.3, 118.2, 126.9, 129.6, 131.3, 134.4, 135.6, 151.9, 158.4, 159.9, 160.8, 161.4, 166.4. HPLC-HRMS-ESI, *m*/*z*([M + H]^+^), calcd for C_24_H_23_FN_4_O_2_S + H^+^ 451.1600, found 451.1595.

*(Z)-1-(2-(4-(4-Chlorophenyl)thiazol-2-yl)hydrazineylidene)-8-fluoro-4,4,6-trimethyl-6-phenyl-5,6-dihydro-4H-pyrrolo[3,2,1-ij]quinolin-2(1H)-one* (**5g**). Orange solid; 0.41 g; yield 77%; m.p. 148–150 °C; ^1^H NMR (400 MHz, DMSO-*d*_6_), δ (ppm): 0.73 (3H, s, C^4^-CH_3_), 1.66 (3H, s, C^4^-CH_3_), 1.69 (3H, s, C^6^-CH_3_), 2.14 (1H, d, *J* = 14.4 Hz, C^5^-H), 2.46–2.54 (1H, m, C^5^-H), 7.06 (2H, d, *J* = 7.6 Hz, Ar–H), 7.16 (1H, t, *J* = 6.0 Hz, Ar–H), 7.20–7.28 (3H, m, Ar–H), 7.32 (1H, dd, *J* = 7.8 Hz, *J* = 2.3 Hz, Ar–H), 7.44 (2H, d, *J* = 8.6 Hz, Ar–H), 7.68 (1H, s, C_thiazole_-H), 7.89 (2H, d, *J* = 8.6 Hz, Ar–H), 13.44 (1H, s, NH). ^13^C NMR (101 MHz, DMSO-*d*_6_), δ (ppm): 25.3, 28.2, 31.1, 40.3, 51.2, 54.7, 105.7, 105.9, 108.4, 112.1, 112.2, 124.2, 126.7, 126.9, 127.8, 128.6, 129.1, 129.3, 131.8, 132.9, 133.2, 135.0, 147.7, 150.3, 158.1, 161.1, 166.3. HPLC-HRMS-ESI, *m*/*z*([M + H]^+^), calcd for C_29_H_24_ClFN_4_OS + H^+^ 531.1417, found 531.1417.

*(Z)-8-Bromo-1-(2-(4-(4-chlorophenyl)thiazol-2-yl)hydrazineylidene)-4,4,6-trimethyl-6-phenyl-5,6-dihydro-4H-pyrrolo[3,2,1-ij]quinolin-2(1H)-one* (**5h**). Yellow solid; 0.48 g; yield 81%; m.p. 286–288 °C; ^1^H NMR (400 MHz, CDCl_3_), δ (ppm): 0.88 (3H, s, C^4^-CH_3_), 1.60–1.83 (6H, m, C^6^-CH_3_ + C^4^-CH_3_), 2.16 (1H, d, *J* = 14.3 Hz, C^5^-H), 2.39 (1H, d, *J* = 14.3 Hz, C^5^-H), 7.00–7.08 (3H, m, C_thiazole_-H + Ar–H), 7.20 (1H, t, *J* = 7.2 Hz, Ar–H), 7.23–7.30 (2H, m, Ar–H), 7.37 (2H, d, *J* = 8.5 Hz, Ar–H), 7.39 (1H, d, *J* = 1.8 Hz, Ar–H), 7.70 (1H, d, *J* = 1.7 Hz, Ar–H), 7.77 (2H, d, *J* = 8.5 Hz, Ar–H), 13.49 (1H, s, NH). ^13^C NMR (101 MHz, CDCl_3_), δ (ppm): 25.4, 28.3, 30.74, 40.1, 52.3, 54.8, 106.0, 115.7, 120.6, 121.3, 121.4, 126.5, 127.2, 127.3, 128.5, 128.6, 128.8, 128.9, 130.7, 130.8, 132.5, 133.8, 137.4, 147.13, 150.7, 160.9, 166.7 HPLC-HRMS-ESI, *m*/*z*([M + H]^+^), calcd for C_29_H_24_BrClN_4_OS + H^+^ 591.0617, found 591.0612.

*(Z)-6-(4-Chlorophenyl)-1-(2-(4-(4-methoxyphenyl)thiazol-2-yl)hydrazineylidene)-4,4,6-trimethyl-5,6-dihydro-4H-pyrrolo[3,2,1-ij]quinolin-2(1H)-one* (**5i**). Orange solid; 0.42 g; yield 77%; m.p. 206–208 °C; ^1^H NMR (400 MHz, DMSO-*d*_6_), δ (ppm): 0.78 (3H, s, C^4^-CH_3_), 1.67 (6H, s, C^4^-CH_3_ + C^6^-CH_3_), 2.16 (1H, d, *J* = 14.4 Hz, C^5^-H), 2.45–2.54 (1H, m, C^5^-H), 3.77 (3H, s, MeO), 6.95 (2H, d, *J* = 8.8 Hz, Ar–H), 7.08 (2H, d, *J* = 8.6 Hz, Ar–H), 7.19 (1H, t, *J* = 7.7 Hz, Ar–H), 7.28 (2H, d, *J* = 8.7 Hz, Ar–H), 7.37 (1H, d, *J* = 7.8 Hz, Ar–H), 7.42 (1H, s, C_thiazole_-H), 7.51 (1H, d, *J* = 7.4 Hz, Ar–H), 7.80 (2H, d, *J* = 8.8 Hz, Ar–H), 13.38 (1H, s, NH). ^13^C NMR (101 MHz, DMSO-*d*_6_), δ (ppm): 25.5, 28.2, 30.8, 39.7, 51.1, 54.6, 55.5, 105.2, 114.4, 118.5, 118.7, 123.3, 126.5, 127.2, 127.5, 128.5, 129.0, 131.3, 132.1, 138.5, 141.5, 147.3, 151.4, 159.5, 161.2, 166.2. HPLC-HRMS-ESI, *m*/*z*([M + H]^+^), calcd for C_30_H_27_ClN_4_O_2_S + H^+^ 543.1617, found 543.1613.

*(Z)-8-Chloro-6-(4-chlorophenyl)-1-(2-(4-(4-methoxyphenyl)thiazol-2-yl)hydrazineylidene)-4,4,6-trimethyl-5,6-dihydro-4H-pyrrolo[3,2,1-ij]quinolin-2(1H)-one* (**5j**). Red solid; 0.49 g; yield 85%; m.p. 264–266 °C; ^1^H NMR (400 MHz, CDCl_3_), δ (ppm): 0.92 (3H, s, C^4^-CH_3_), 1.67–1.76 (6H, m, C^4^-CH_3_+ C^6^-CH_3_), 2.16 (1H, d, *J* = 14.3 Hz, C^5^-H), 2.34 (1H, d, *J* = 14.4 Hz, C^5^-H), 3.84 (3H, s, MeO), 6.91 (1H, s, C_thiazole_-H), 6.94 (2H, d, *J* = 8.7 Hz, Ar–H), 6.99 (2H, d, *J* = 8.6 Hz, Ar–H), 7.20 (1H, d, *J* = 1.8 Hz, Ar–H), 7.23 (2H, d, *J* = 8.5 Hz, Ar–H), 7.57 (1H, d, *J* = 1.8 Hz, Ar–H), 7.77 (2H, d, *J* = 8.7 Hz, Ar–H), 13.50 (1H, s, NH). ^13^C NMR (101 MHz, CDCl_3_), δ (ppm): 25.6, 28.2, 30.8, 39.8, 52.2, 54.7, 55.4, 103.7, 114.1, 118.7, 120.4, 127.3, 127.4, 127.4, 127.7, 122.0, 128.4, 128.6, 128.6, 132.4, 136.9, 145.8, 146.9, 159.7, 160.9, 166.7. HPLC-HRMS-ESI, *m*/*z*([M + H]^+^), calcd for C_30_H_26_Cl_2_N_4_O_2_S + H^+^ 577.1228, found 577.1223.

*(Z)-6-(4-Chlorophenyl)-1-(2-(4-(4-chlorophenyl)thiazol-2-yl)hydrazineylidene)-8-fluoro-4,4,6-trimethyl-5,6-dihydro-4H-pyrrolo[3,2,1-ij]quinolin-2(1H)-one* (**5k**). Yellow-orange solid; 0.44 g; yield 78%; m.p. 284–286 °C; ^1^H NMR (400 MHz, DMSO-*d*_6_), δ (ppm): 0.77 (3H, s, C^4^-CH_3_), 1.62–1.72 (6H, m, C^6^-CH_3_ + C^4^-CH_3_), 2.15 (1H, d, *J* = 14.3 Hz, C^5^-H), 2.45–2.56 (1H, m, C^5^-H), 7.10 (2H, d, *J* = 8.6 Hz, Ar–H), 7.32–7.24 (3H, m, Ar–H), 7.34 (1H, dd, *J* = 8.0 Hz, *J* = 2.5 Hz, Ar–H), 7.45 (2H, d, *J* = 8.5 Hz, Ar–H), 7.70 (1H, s, C_thiazole_-H), 7.90 (2H, d, *J* = 8.5 Hz, Ar–H), 13.43 (1H, s, NH). ^13^C NMR (101 MHz, DMSO-*d*_6_), δ (ppm): 25.5, 28.1, 30.6, 40.1, 52.4, 54.7, 108.5, 115.1, 115.2, 119.4, 119.5, 122. 8, 122.9, 127.8, 128.6, 128.9, 129.1, 129.3, 131.3, 131.4, 132.9, 133.2, 146.8, 150.3, 158.2, 160.5, 161.1, 166.3. HPLC-HRMS-ESI, *m*/*z*([M + H]^+^), calcd for C_29_H_23_Cl_2_FN_4_OS + H^+^ 565.1027, found 565.1025.

*(Z)-6-(4-Chlorophenyl)-8-fluoro-1-(2-(4-(4-fluorophenyl)thiazol-2-yl)hydrazineylidene)-4,4,6-trimethyl-5,6-dihydro-4H-pyrrolo[3,2,1-ij]quinolin-2(1H)-one* (**5l**). Yellow solid; 0.39 g; yield 71%; m.p. 254–256 °C; ^1^H NMR (400 MHz, DMSO-*d*_6_), δ (ppm): 0.76 (3H, s, C^4^-CH_3_), 1.63–1.71 (6H, m, C^4^-CH_3_ + C^6^-CH_3_), 2.15 (1H, d, *J* = 14.8 Hz, C^5^-H), 2.46–2.56 (1H, m, C^5^-H), 7.11 (2H, d, *J* = 8.6 Hz, Ar–H), 7.24 (2H, t, *J* = 8.8 Hz, Ar–H), 7.27–7.39 (4H, m, Ar–H), 7.64 (1H, s, C_thiazole_-H), 7.92 (2H, dd, *J* = 8.7 Hz, *J* = 5.6 Hz, Ar–H), 13.42 (1H, s, NH). ^13^C NMR (101 MHz, DMSO-*d*_6_), δ (ppm): 25.5, 28.1, 30.6, 40.1, 50.9, 54.7, 105.8, 106.1, 107.6, 115.3, 115.5, 115.9, 116.1, 119.8, 119.9, 128.2, 128.3, 128.6, 129.0, 130.9, 131.0, 131.2, 131.4, 131.7, 131.7, 134.9, 146.8, 150.5, 158.2, 160.5, 161.1, 166.3. HPLC-HRMS-ESI, *m*/*z*([M + H]^+^), calcd for C_29_H_23_ClF_2_N_4_OS + H^+^ 549.1323, found 549.1327.

*(Z)-6-(4-Chlorophenyl)-1-(2-(4-(3-chlorophenyl)thiazol-2-yl)hydrazineylidene)-8-fluoro-4,4,6-trimethyl-5,6-dihydro-4H-pyrrolo[3,2,1-ij]quinolin-2(1H)-one* (**5m**). Yellow solid; 0.39 g; yield 69%; m.p. 238–240 °C; ^1^H NMR (400 MHz, CDCl_3_), δ (ppm): 0.91 (3H, s, C^4^-CH_3_), 1.70 (3H, s, C^4^-CH_3_), 1.74 (3H, s, C^6^-CH_3_), 2.17 (1H, d, *J* = 14.3 Hz, C^5^-H), 2.35 (1H, d, *J* = 14.3 Hz, C^5^-H), 6.95 (1H, dd, *J* = 10.1 Hz, *J* = 2.3 Hz, Ar–H), 6.99 (2H, d, *J* = 8.5 Hz, Ar–H), 7.06 (1H, s, C_thiazole_-H), 7.23 (2H, d, *J* = 8.3 Hz, Ar–H), 7.27–7.38 (3H, m, Ar–H), 7.71 (1H, d, *J* = 7.6 Hz, Ar–H), 7.83 (1H, s, Ar–H), 13.52 (1H, s, NH). ^13^C NMR (101 MHz, CDCl_3_), δ (ppm): 25.6, 28.3, 30.8, 39.9, 52.2, 54.6, 106.7, 114.4, 114.6, 120.1, 120.2, 124.0, 124.1, 126.0, 126.2, 127.2, 127.3, 128.0, 128.2, 128.3, 130.0, 131.3, 132.4, 134.5, 134.6, 135.8, 145.8, 150.5, 158.3, 160.6, 161.2, 166.7. HPLC-HRMS-ESI, *m*/*z*([M + H]^+^), calcd for C_29_H_23_Cl_2_FN_4_OS + H^+^ 565.1027, found 565.1026.

*(Z)-6-(4-Chlorophenyl)-8-fluoro-1-(2-(4-(3-methoxyphenyl)thiazol-2-yl)hydrazineylidene)-4,4,6-trimethyl-5,6-dihydro-4H-pyrrolo[3,2,1-ij]quinolin-2(1H)-one* (**5n**). Yellow solid; 0.41 g; yield 73%; m.p. 231–233 °C; ^1^H NMR (400 MHz, DMSO-*d*_6_), δ (ppm): 0.78 (3H, s, C^4^-CH_3_), 1.67 (6H, s, C^4^-CH_3_ + C^6^ -CH_3_), 2.14 (1H, d, *J* = 15.1 Hz, C^5^-H), 2.43–2.56 (1H, m, C^5^-H), 3.79 (3H, s, MeO), 6.85 (1H, d, *J* = 8.8 Hz, Ar–H), 7.08 (2H, d, *J* = 7.7 Hz, Ar–H), 7.21 (1H, d, *J* = 9.7 Hz, Ar–H), 7.24–7.51 (4H, m, Ar–H), 7.61 (1H, s, C_thiazole_-H), 13.43 (1H, s, NH). ^13^C NMR (101 MHz, DMSO-*d*_6_), δ (ppm): 25.5, 28.1, 30.7, 40.5, 51.1, 54.6, 55.5, 105.8, 106.0, 107.9, 111.4, 114.2, 115.0, 115.3, 118.4, 119.8, 119.9, 128.5, 128.6, 128.8, 130.1, 131.5, 134.8, 135.7, 146.6, 151.5, 158.1, 160.0, 161.1, 166.0. HPLC-HRMS-ESI, *m*/*z*([M + H]^+^), calcd for C_30_H_26_ClFN_4_O_2_S + H^+^ 561.1523, found 561.1521.

*(Z)-8-Bromo-6-(4-chlorophenyl)-4,4,6-trimethyl-1-(2-(4-phenylthiazol-2-yl)hydrazineylidene)-5,6-dihydro-4H-pyrrolo[3,2,1-ij]quinolin-2(1H)-one* (**5o**). Yellow-orange solid; 0.48 g; yield 81%; m.p. 265–267 °C; ^1^H NMR (400 MHz, CDCl_3_), δ (ppm): 0.92 (3H, s, C^4^-CH_3_), 1.58–1.83 (6H, m, C^4^-CH_3_ + C^6^-CH_3_), 2.16 (1H, d, *J* = 14.3 Hz, C^5^-H), 2.34 (1H, d, *J* = 14.3 Hz, C^5^-H), 6.99 (2H, d, *J* = 8.0 Hz, Ar–H), 7.06 (1H, s, C_thiazole_-H), 7.15–7.28(2H, m, Ar–H), 7.28–7.37 (2H, m, Ar–H), 7.41 (2H, t, *J* = 7.2 Hz, Ar–H), 7.71 (1H, s, Ar–H), 7.84 (2H, d, *J* = 7.3 Hz, Ar–H), 13.47 (1H, s, NH). ^13^C NMR (101 MHz, CDCl_3_), δ (ppm): 25.7, 28.3, 30.8, 39.8, 52.2, 54.7, 105.8, 115.7, 120.8, 121.4, 126.0, 127.9, 128.0, 128.1, 128.6, 128.7, 130.3, 130.3, 132.4, 134.1, 137.2, 145.8, 152.0, 160.9, 166.4. HPLC-HRMS-ESI, *m*/*z*([M + H]^+^), calcd for C_29_H_24_BrClN_4_OS + H^+^ 591.0617, found 591.0612.

### 3.2. X-Ray Diffraction

X-ray diffraction data for **5d** were collected at 100K on a four-circle Rigaku Synergy S diffractometer equipped with a HyPix6000HE area detector (kappa geometry, shutterless ω-scan technique) using graphite monochromated Cu K_α_ radiation. The intensity data were integrated and corrected for absorption and decay with the CrysAlisPro program (Version 1.171.41.106a) [45].Using OLEX2 (Version 1.3) [46], the structure was solved with the SHELXT structure solution program [47] using intrinsic phasing and refined with the SHELXL-2018 refinement package [48] using least-squares minimization against F^2^ in the anisotropic approximation for non-hydrogen atoms. The hydrogen atom of the NH group was found from difference Fourier synthesis and refined in the isotropic approximation. Positions of other hydrogen atoms were calculated, and they were refined in the isotropic approximation in the riding model. Crystal data and structure refinement parameters are given in Supplementary Materials, Table S1. Deposition Number CCDC 2393399 contains the supplementary crystallographic data for this paper. These data can be obtained free of charge via https://www.ccdc.cam.ac.uk/structures, accessed on 26 August 2025 (or from the CCDC, 12 Union Road, Cambridge CB2 1EZ, UK; Fax: +44 1223 336033; e-mail: deposit@ccdc.cam.ac.uk).

### 3.3. In Vitro Assays

The inhibition of the blood coagulation factors Xa and XIa or plasmin by the synthesized compounds **5a**–**o** was studied by measuring the kinetics of hydrolysis of substrates specific for each of these enzymes in the presence of the compounds. A specific low-molecular-weight chromogenic substrate S2765 (Z-D-Arg-Gly-Arg-pNA·2HCl) was used in the case of factor Xa, substrate S2366 (pyroGlu-Pro-Arg-pNA·HCl) was used for factor XIa, and S2288 (H-D-Ile-Pro-Arg-pNA·2HCl) was used for plasmin (all from Chromogenix, West Chester, PA, USA). A buffer containing 140 mM NaCl, 20 mM HEPES, and 0.1% PEG 6000 (pH 8.0) was placed in the wells of a 96-well plate, followed by the addition of factor Xa or XIa (final concentration 5 nmol·L^−1^) or plasmin (final concentration 10 nmol L^−1^); the substrate S2765, S2366, or S2288 (final concentration 200 µmol·L^−1^); and a solution of the test compound in DMSO (final concentration 30 µmol·L^−1^, the DMSO content in the well was no more than 2%). The kinetics of the formation of p-nitroaniline was measured using an Eppendorf PlateReader AF2200 microplate reader (Eppendorf, Hamburg, Germany) by the absorption of light with a wavelength of 405 nm. The initial rate of substrate degradation was determined from the initial slope of the 4-nitroaniline formation curve. Percent inhibition was calculated using the following formula:

% inhibition = 100 × (1 − initial rate of substrate degradation in presence of inhibitor/initial rate of substrate degradation without inhibitor)

The absolute value of negative inhibition values denotes percent activation.

The data obtained were processed using the GraphPad Prism 8.0.1 (244) and OriginPro 8 software.

### 3.4. Molecular Docking Studies

To comprehend the molecular binding mechanisms of the active compounds toward two distinct biological targets that are associated with FXa and FXIa, molecular docking studies were conducted. The protein structure of FXa was taken from the 3CEN complex, and the 4CRC complex was used to prepare FXIa protein from the Protein Data Bank [49] (PDB), which was taken into consideration for docking simulations. In the docking simulations, the proteins were considered without co-crystallized ligands. Validation of prepared protein models implied docking of the corresponding co-crystallized ligand. For both FXa and FXIa, reproduction of co-crystallized ligand conformation with an RMSD value less than 1.5 Å was obtained.

All docking calculations were performed using the AutoDock 4.2 [50] software. The docking input files were created, and the docking outcomes were analyzed using the AutoDockTools [50] software. A 90 × 90 × 90 grid box was created, covering nearly the whole surface of the protein, with grid points spaced 0.375 Å apart. Before the computations, all non-polar hydrogens and crystallographic water molecules were eliminated. The mass center of the TSA that was bound served as the docking grid’s center. Using genetic algorithm searches, 100 docked structures were produced in each case.

A default protocol was used, with an initial population of 50 randomly arranged conformations. Heavy atom comparison root mean square deviations (RMSD values) were determined, and initial ligand binding modes were plotted. Plots of protein–ligand interactions were generated using the LigandScout software (Version 4.4.5) [51].

## 4. Conclusions

Herein, a detailed investigation into the design and synthesis of novel hybrid molecules featuring a pyrrolo[3,2,1-*ij*]quinolin-2-one core linked to a thiazole moiety via a hydrazine linker is presented. Fifteen novel hybrids—1-(2-(4-arylthiazol-2-yl)hydrazineylidene)-5,6-dihydro-4*H*-pyrrolo[3,2,1-*ij*]quinolin-2-ones—were synthesized. The chemical structures of the compounds were confirmed using a comprehensive set of spectral data, including HPLC-MS, ^1^H NMR, and ^13^C NMR, complemented by X-ray structural analysis of compound **5d**, which indicated that compounds **5** exist in the *Z*-configuration. Compounds were evaluated for their potential as anticoagulant agents, specifically as dual inhibitors of coagulation factors Xa and XIa. Preliminary in vitro screening for inhibitory activity against FXa and FXIa revealed compounds **5d** and **5h** as the most promising candidates, exhibiting significant dual inhibitory activity within the studied series. Structure–activity relationship studies demonstrate that the introduction of halogen atoms at position 8 of pyrrolo[3,2,1-*ij*]quinolin-2-one, as well as in the para-position of the aryl substituent in the thiazole ring, is beneficial for the activity. Finally, docking studies confirmed the obtained results. These findings emphasize the promise of a hybrid molecule strategy for developing multitarget compounds and highlight the considerable potential of the target structures in advancing the development of next-generation anticoagulants. Compounds **5d** and **5h** can be considered as lead compounds for the development of new derivatives with better activity and safer profile.

## Figures and Tables

**Table 1:** Anticoagulant activity of compounds **5a**–**o**.

No.	Percent Inhibition at 30 μM ± SEM (n = 3)			IC_50_, μM ± SEM (n = 3)	
	Xa	XIa	Plasmin	Xa	XIa
**5a**	−30 ± 7	−13 ± 11			
**5b**	−30 ± 10	−6 ± 10			
**5c**	−40 ± 10	−7 ± 8			
**5d**	98 ± 1	99	5 ± 1	5.43 ± 0.02	7.70 ± 0.05
**5e**	−8 ± 10	−2 ± 11			
**5f**	−25 ± 9	−11 ± 9			
**5g**	51 ± 18	76 ± 3			
**5h**	99 ± 1	100	8 ± 2	5.75 ± 0.02	7.66 ± 0.08
**5i**	−21 ± 9	−14 ± 10			
**5j**	−3 ± 3	−3 ± 6			
**5k**	−12 ± 11	37 ± 8			
**5l**	−4 ± 3	−11 ± 7			
**5m**	−17 ± 11	−14 ± 7			
**5n**	−2 ± 3	−19 ± 8			
**5o**	−4 ± 6	−11 ± 12			
Rivaroxaban	94	8		0.007 ± 0.001	

The absolute value of negative inhibition values denotes percent activation.

**Table 2:** Molecular docking free binding energies (kcal/mol) to factor Xa and factor Xia.

No.	Est. Binding Energy (kcal/mol)		No.	Est. Binding Energy (kcal/mol)	
	FXa	FXIa		FXa	FXIa
**5a**	−6.62	−4.28	**5i**	−5.14	−4.67
**5b**	−6.44	-	**5j**	-	-
**5c**	−6.48	-	**5k**	−1.56	-
**5d**	−11.56	−11.60	**5l**	-	−2.56
**5e**	−1.28	-	**5m**	−4.63	−3.10
**5f**	−2.63	−1.34	**5n**	−1.87	-
**5g**	−6.59	−7.17	**5o**	-	−3.14
**5h**	−12.47	−12.80	**Rivaroxaban**	−13.48	−5.34

**Table 3:** Drug-likeness of the tested compounds.

Property		Predicted Value							Unit
	5a	5b	5c	5d	5e	5f	5g	5h	
Molecular weight	466.98	450.53	511.43	454.95	466.98	450.53	531.04	591.95	Numeric (g/mol)
Num. rotatable bonds	4	4	4	3	4	4	4	4	Numeric
Num. H-bond acceptors	4	5	4	4	4	5	4	3	Numeric
Num. H-bond donors	1	1	1	1	1	1	1	1	Numeric
TPSA	95.06	95.06	95.06	85.83	95.06	95.06	85.83	85.83	Numeric (Å^2^)
Consensus log*P*_o/w_	5.02	4.81	5.10	5.32	4.98	4.76	6.55	685	average of five methods
Solubility	Poor	Poor	Poor	Poor	Poor	Poor	Poor	Poor	Categorical
GI absorption	High	High	High	High	High	High	Low	Low	Categorical
P-gp substrate	Yes	Yes	Yes	Yes	Yes	Yes	No	No	Categorical
CYP2D6 inhibitor	No	No	No	No	No	No	No	Yes	Categorical
CYP3A4 inhibitor	Yes	Yes	Yes	Yes	Yes	Yes	No	No	Categorical
Lipinski	0	0	1	1	0	0	2	2	Categorical
Bioavailability Score	0.55	0.55	0.55	0.55	0.55	0.55	0.17	0.17	
PAINS	1 *	1 *	1 *	1 *	1 *	1 *	1 *	1 *	Categorical
-	**5i**	**5j**	**5k**	**5l**	**5m**	**5n**	**5o**		
Molecular weight	543.08	577.52	565.49	549.03	565.49	561.07	591.95		Numeric (g/mol)
Num. rotatable bonds	5	5	4	4	4	5	4		Numeric
Num. H-bond acceptors	4	4	4	5	4	5	3		Numeric
Num. H-bond donors	1	1	1	1	1	1	1		Numeric
TPSA	95.06	95.06	85.83	85.83	85.83	95.06	85.83		Numeric (Å^2^)
Consensus log*P*_o/w_	6.18	6.67	7.03	8.32	7.04	6.45	6.85		average of five methods
Solubility	Poor	Poor	Poor	Poor	Poor	Poor	Poor		Categorical
GI absorption	Low	Low	Low	Low	Low	Low	Low		Categorical
P-gp substrate	No	No	No	No	No	No	No		Categorical
CYP2D6 inhibitor	Yes	Yes	Yes	No	Yes	Yes	Yes		Categorical
CYP3A4 inhibitor	No	No	No	No	No	No	No		Categorical
Lipinski	2	2	2	2	2	2	2		Categorical
Bioavailability Score	0.17	0.17	0.17	0.17	0.17	0.17	0.17		
PAINS	1 *	1 *	1 *	1 *	1 *	1 *	1 *		Categorical

Consensus log*P*_o/w_: consensus logarithm of partition coefficient (octanol/water) predicted as an average of five methods (iLOGP, XLOGP3, WLOGP, MLOGP, and SILICOS-IT); TPSA: topological polar surface area (Å). * 1 alert: imine_one_isatin 
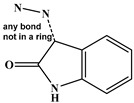
.

**Table 4:** ADMET values for the tested compounds.

Property	Model Name	Predicted Value								Unit
		Rivaroxaban	5a	5b	5c	5d	5e	5f	5g	
**Absorption**	Water solubility	−4.606	−5.766	−5.166	−5.831	−5.517	−5.525	−5.296	−6.060	Numeric (log mol/L)
**Absorption**	Caco2 permeability	1.049	0.71	1.111	0.702	1.101	1.121	1.119	1.063	Numeric (log Papp in 10^−6^ cm/s)
**Absorption**	Intestinal absorption (human)	92.743	91.439	92.534	91.372	90.499	91.907	93.014	91.282	Numeric (% absorbed)
**Absorption**	Skin permeability	−3.186	−2.768	−2.793	−2.767	−2.769	−2.789	−2.801	−2.724	Numeric (log Kp)
**Absorption**	P-glycoprotein substrate	No	Yes	Yes	Yes	Yes	Yes	Yes	Yes	Categorical (Yes/No)
**Absorption**	P-glycoprotein I inhibitor	Yes	Yes	Yes	Yes	Yes	Yes	Yes	Yes	Categorical (Yes/No)
**Absorption**	P-glycoprotein II inhibitor	Yes	Yes	Yes	Yes	Yes	Yes	Yes	Yes	Categorical (Yes/No)
**Distribution**	VDss (human)	−0.625	0.464	0.435	0484	0.509	0.581	0.388	0.471	Numeric (log L/kg)
**Distribution**	Fraction unbound (human)	0.002	0.01	0.065	0.007	0.062	0.021	0.022	0.155	Numeric (Fu)
**Distribution**	BBB permeability	−1.022	−0.054	−0.180	−0.055	0.075	−0.047	−0.201	0.031	Numeric (log BB)
**Distribution**	CNS permeability	−2.644	−1.588	−1.78	−1.565	−1.494	−1.622	−1.773	−1.218	Numeric (log PS)
**Metabolism**	CYP2D6 substrate	No	No	No	No	No	No	No	No	Categorical (Yes/No)
**Metabolism**	CYP3A4 substrate	Yes	Yes	Yes	Yes	Yes	Yes	Yes	Yes	Categorical (Yes/No)
**Metabolism**	CYP2D6 inhibitor	No	No	No	No	No	No	No	No	Categorical (Yes/No)
**Metabolism**	CYP3A4 inhibitor	Yes	Yes	Yes	Yes	Yes	Yes	Yes	Yes	Categorical (Yes/No)
**Excretion**	Total clearance	0.294	−0.01	−0.142	−0.032	−0.308	−0.128	−0.025	−0.189	Numeric (log ml/min/kg)
**Excretion**	Renal OCT2 substrate	No	No	No	No	No	No	No	No	Categorical (Yes/No)
**Toxicity**	AMES toxicity	Yes	No	No	No	No	No	No	No	Categorical (Yes/No)
**Toxicity**	Max. tolerated dose (human)	0.495	0.259	0.168	0.254	0.198	0.206	0.173	0.478	Numeric (log mg/kg/day)
**Toxicity**	Oral rat acute toxicity (LD50)	2.704	3.061	3.128	3.064	3.105	3.128	3.043	2.754	Numeric (mol/kg)
**Toxicity**	hERG I inhibitor	No	No	No	No	No	No	No	No	Categorical (Yes/No)
**Toxicity**	Hepatotoxicity	Yes	Yes	Yes	Yes	Yes	Yes	Yes	Yes	Categorical (Yes/No)
**Toxicity**	Skin Sensitization	No	No	No	No	No	No	No	No	Categorical (Yes/No)
**Property**	**Model name**	**Predicted value**								**Unit**
		**5h**	**5i**	**5j**	**5k**	**5l**	**5m**	**5n**	**5o**	
**Absorption**	Water solubility	−6.225	−5.647	−5.702	−6.044	−5.441	−6.026	−5.634	−6.193	Numeric (log mol/L)
**Absorption**	Caco2 permeability	1.037	1.036	1.054	1.056	1.097	1.05	1.052	1.039	Numeric (log Papp in 10^−6^ cm/s)
**Absorption**	Intestinal absorption (human)	90.174	94.393	92.432	90.970	92.065	91.731	93.539	91.357	Numeric (% Absorbed)
**Absorption**	Skin permeability	−2.723	−2.728	−2.728	−2.723	−2.731	−2.733	−2.728	−2.721	Numeric (log Kp)
**Absorption**	P-glycoprotein substrate	Yes	Yes	Yes	Yes	Yes	Yes	Yes	Yes	Categorical (Yes/No)
**Absorption**	P-glycoprotein I inhibitor	Yes	Yes	Yes	Yes	Yes	Yes	Yes	Yes	Categorical (Yes/No)
**Absorption**	P-glycoprotein II inhibitor	Yes	Yes	Yes	Yes	Yes	Yes	Yes	Yes	Categorical (Yes/No)
**Distribution**	VDss (human)	0.618	0.619	0.684	0.575	0.467	0.574	0.535	0.648	Numeric (log L/kg)
**Distribution**	Fraction unbound (human)	0.128	0.165	0.153	0.164	0.222	0.152	0.152	0.128	Numeric (Fu)
**Distribution**	BBB permeability	0.156	−0.075	−0.081	0.042	−0.085	0.05	−0.235	0.215	Numeric (log BB)
**Distribution**	CNS permeability	−1.037	−1.392	−1.272	−1.111	−1.303	−1.113	−1.424	−1.046	Numeric (log PS)
**Metabolism**	CYP2D6 substrate	No	No	No	No	No	No	No	No	Categorical (Yes/No)
**Metabolism**	CYP3A4 substrate	Yes	Yes	Yes	Yes	Yes	Yes	Yes	Yes	Categorical (Yes/No)
**Metabolism**	CYP2D6 inhibitor	No	No	No	No	No	No	No	No	Categorical (Yes/No)
**Metabolism**	CYP3A4 inhibitor	Yes	Yes	Yes	Yes	Yes	Yes	Yes	Yes	Categorical (Yes/No)
**Excretion**	Total clearance	−0.195	−0.125	−0.254	−0.32	−0.451	−0.314	−0.152	−0.194	Numeric (log ml/min/kg)
**Excretion**	Renal OCT2 substrate	No	No	No	No	No	No	No	No	Categorical (Yes/No)
**Toxicity**	AMES toxicity	Yes	No	No	Yes	No	Yes	No	Yes	Categorical (Yes/No)
**Toxicity**	Max. tolerated dose (human)	0.448	0.470	0.468	0.429	0.562	0.426	0.474	0.448	Numeric (log mg/kg/day)
**Toxicity**	Oral rat acute toxicity (LD50)	2.705	2.964	2.952	2.831	2.941	2.825	2.962	2.728	Numeric (mol/kg)
**Toxicity**	hERG I inhibitor	No	No	No	No	No	No	No	No	Categorical (Yes/No)
**Toxicity**	Hepatotoxicity	No	Yes	Yes	Yes	Yes	Yes	Yes	No	Categorical (Yes/No)
**Toxicity**	Skin sensitization	No	No	No	No	No	No	No	No	Categorical (Yes/No)

## Data Availability

See the Supplementary Material containing ^1^H and ^13^C NMR, data of HPLC-MS-ESI analysis for new synthesized compounds (Figures S1–S69), analysis of the dependence of inhibition of factor Xa- and XIa-induced chromogenic substrate hydrolysis on the concentration of compounds (Figures S70–S73), and crystal data and structure refinement parameters for **5d** (Table S1).

## Supplementary Materials

The following supporting information can be downloaded at https://www.mdpi.com/article/10.3390/molecules30173544/s1. Along with the manuscript, copies of ^1^H- and ^13^C-NMR spectra, data of HPLC-MS-ESI analysis for all new synthesized compounds (Figures S1–S69), analysis of the dependence of inhibition of factor Xa- and XIa-induced chromogenic substrate hydrolysis on the concentration of compounds **5d** and **5h** (Figures S70–S73), and crystal data and structure refinement parameters for **5d** (Table S1) have been submitted.
