# Supplementary material for: Design, Synthesis, and In Vitro and In Silico Study of New Hybrid 1-(2-(4-Arylthiazol-2-yl)hydrazineylidene)-5,6-dihydro-4H-pyrrolo[3,2,1-ij]quinolin-2-ones as Factor Xa and Factor XIa Inhibitors
Source: Molecules. 2025 Aug 29;30(17):3544. doi: 10.3390/molecules30173544 (PMC12430708; doi:10.3390/molecules30173544)
Supplement: Supplementary file 1 [file molecules-30-03544-s001.zip › molecules-3787334-supplementary.pdf]

Electronic Supplementary Material file

Design, Synthesis, and In Vitro and In Silico Study of New Hybrid 1-(2-(4-arylthiazol-2-yl)hydrazineylidene)-5,6-dihydro-4*H*-pyrrolo[3,2,1-*ij*]quinolin-2-ones as Factor Xa and Factor XIa Inhibitors

Anna A. Skoptsova [1], Athina Geronikaki [2,*], Anthi Petrou [2], Nadezhda P. Novichikhina [1], Nadezhda A. Podoplelova [3], Georgii A. Bykov [3], Aleksandr A. Anis'kov [4], Svetlana A. Soloveva [5] and Khidmet S. Shikhaliev [1,*]

1 Department of Organic Chemistry, Faculty of Chemistry, Voronezh State University, 1 Universitetskaya Sq., 394018 Voronezh, Russia; annk0611@mail.ru (A.A.S.); novichikhina@chem.vsu.ru (N.P.N.)
2 School of Pharmacy, Aristotle University of Thessaloniki, 54124 Thessaloniki, Greece; anthi.petrou.thessaloniki1@gmail.com
3 Center for Theoretical Problems of Physicochemical Pharmakology, 119991 Moscow, Russia; podoplelova.nadezhda@ctppcp.ru (N.A.P.); bga20000@gmail.com (G.A.B.)
4 R&D Department, VIC Animal Health, VIC Group, 308570 Belgorod, Russia; aniskovalvis@gmail.com
5 Nesmeyanov Institute of Organoelement Compounds, Russian Academy of Sciences, 119334 Moscow, Russia; aksenova.sa@phystech.edu
* Correspondence: geronik@pharm.auth.gr (A.G.); shikh1961@yandex.ru (K.S.S.)

Contents

Copies of spectral data of synthesized compounds S4

^{1}H, ^{13}C NMR and data HPLC-HRMS-ESI spectra of (*Z*)-2-(8-chloro-4,4,6-trimethyl-2-oxo-5,6-dihydro-4*H*-pyrrolo[3,2,1-*ij*]quinolin-1(2*H*)-ylidene)hydrazine-1-carbothioamide 3b S4

^{1}H, ^{13}C NMR and data HPLC-HRMS-ESI spectra of (*Z*)-2-(8-fluoro-4,4,6-trimethyl-2-oxo-5,6-dihydro-4*H*-pyrrolo[3,2,1-*ij*]quinolin-1(2*H*)-ylidene)hydrazine-1-carbothioamide 3c S5

^{1}H, ^{13}C NMR and data HPLC-HRMS-ESI spectra of (*Z*)-2-(8-fluoro-4,4,6-trimethyl-2-oxo-6-phenyl-5,6-dihydro-4*H*-pyrrolo[3,2,1-*ij*]quinolin-1(2*H*)-ylidene)hydrazine-1-carbothioamide 3d S7

^{1}H, ^{13}C NMR and data HPLC-HRMS-ESI spectra of (*Z*)-2-(8-bromo-4,4,6-trimethyl-2-oxo-6-phenyl-5,6-dihydro-4*H*-pyrrolo[3,2,1-*ij*]quinolin-1(2*H*)-ylidene)hydrazine-1-carbothioamide 3e S8

^{1}H, ^{13}C NMR and data HPLC-HRMS-ESI spectra of (*Z*)-2-(6-(4-chlorophenyl)-4,4,6-trimethyl-2-oxo-5,6-dihydro-4*H*-pyrrolo[3,2,1-*ij*]quinolin-1(2*H*)-ylidene)hydrazine-1-carbothioamide 3f S10

^{1}H, ^{13}C NMR and data HPLC-HRMS-ESI spectra of (*Z*)-2-(8-chloro-6-(4-chlorophenyl)-4,4,6-trimethyl-2-oxo-5,6-dihydro-4*H*-pyrrolo[3,2,1-*ij*]quinolin-1(2*H*)-ylidene)hydrazine-1-carbothioamide 3g S11

^{1}H, ^{13}C NMR and data HPLC-HRMS-ESI spectra of (*Z*)-2-(6-(4-chlorophenyl)-8-fluoro-4,4,6-trimethyl-2-oxo-5,6-dihydro-4*H*-pyrrolo[3,2,1-*ij*]quinolin-1(2*H*)-ylidene)hydrazine-1-carbothioamide 3h S13

^{1}H, ^{13}C NMR and data HPLC-HRMS-ESI spectra of (*Z*)-2-(8-bromo-6-(4-chlorophenyl)-4,4,6-trimethyl-2-oxo-5,6-dihydro-4*H*-pyrrolo[3,2,1-*ij*]quinolin-1(2*H*)-ylidene)hydrazine-1-carbothioamide 3i S14

^{1}H, ^{13}C NMR and data HPLC-HRMS-ESI spectra of (*Z*)-1-(2-(4-(4-chlorophenyl)thiazol-2-yl)hydrazineylidene)-8-methoxy-4,4,6-trimethyl-5,6-dihydro-4*H*-pyrrolo[3,2,1-*ij*]quinolin-2(1*H*)-one 5a S16

^{1}H, ^{13}C NMR and data HPLC-HRMS-ESI spectra of (*Z*)-1-(2-(4-(4-fluorophenyl)thiazol-2-yl)hydrazineylidene)-8-methoxy-4,4,6-trimethyl-5,6-dihydro-4*H*-pyrrolo[3,2,1-*ij*]quinolin-2(1*H*)-one 5b S17

^{1}H, ^{13}C NMR and data HPLC-HRMS-ESI spectra of (*Z*)-1-(2-(4-(4-bromophenyl)thiazol-2-yl)hydrazineylidene)-8-methoxy-4,4,6-trimethyl-5,6-dihydro-4*H*-pyrrolo[3,2,1-*ij*]quinolin-2(1*H*)-one 5c S19

^{1}H, ^{13}C NMR and data HPLC-HRMS-ESI spectra of (*Z*)-8-Chloro-1-(2-(4-(4-fluorophenyl)thiazol-2-yl)hydrazineylidene)-4,4,6-trimethyl-5,6-dihydro-4*H*-pyrrolo[3,2,1-*ij*]quinolin-2(1*H*)-one 5d S20

^{1}H, ^{13}C NMR and data HPLC-HRMS-ESI spectra of (*Z*)-8-chloro-1-(2-(4-(4-methoxyphenyl)thiazol-2-yl)hydrazineylidene)-4,4,6-trimethyl-5,6-dihydro-4*H*-pyrrolo[3,2,1-*ij*]quinolin-2(1*H*)-one 5e S22

^{1}H, ^{13}C NMR and data HPLC-HRMS-ESI spectra of (*Z*)-8-fluoro-1-(2-(4-(3-methoxyphenyl)thiazol-2-yl)hydrazineylidene)-4,4,6-trimethyl-5,6-dihydro-4*H*-pyrrolo[3,2,1-*ij*]quinolin-2(1*H*)-one 5f S23

^{1}H, ^{13}C NMR and data HPLC-HRMS-ESI spectra of (*Z*)-1-(2-(4-(4-chlorophenyl)thiazol-2-yl)hydrazineylidene)-8-fluoro-4,4,6-trimethyl-6-phenyl-5,6-dihydro-4*H*-pyrrolo[3,2,1-*ij*]quinolin-2(1*H*)-one 5g S25

^{1}H, ^{13}C NMR and data HPLC-HRMS-ESI spectra of (*Z*)-8-bromo-1-(2-(4-(4-chlorophenyl)thiazol-2-yl)hydrazineylidene)-4,4,6-trimethyl-6-phenyl-5,6-dihydro-4*H*-pyrrolo[3,2,1-*ij*]quinolin-2(1*H*)-one 5h S26

^{1}H, ^{13}C NMR and data HPLC-HRMS-ESI spectra of (*Z*)-6-(4-chlorophenyl)-1-(2-(4-(4-methoxyphenyl)thiazol-2-yl)hydrazineylidene)-4,4,6-trimethyl-5,6-dihydro-4*H*-pyrrolo[3,2,1-*ij*]quinolin-2(1*H*)-one 5i S28

^{1}H, ^{13}C NMR and data HPLC-HRMS-ESI spectra of (*Z*)-8-chloro-6-(4-chlorophenyl)-1-(2-(4-(4-methoxyphenyl)thiazol-2-yl)hydrazineylidene)-4,4,6-trimethyl-5,6-dihydro-4*H*-pyrrolo[3,2,1-*ij*]quinolin-2(1*H*)-one 5j S29

^{1}H, ^{13}C NMR and data HPLC-HRMS-ESI spectra of (*Z*)-6-(4-chlorophenyl)-1-(2-(4-(4-chlorophenyl)thiazol-2-yl)hydrazineylidene)-8-fluoro-4,4,6-trimethyl-5,6-dihydro-4*H*-pyrrolo[3,2,1-*ij*]quinolin-2(1*H*)-one 5k S31

^{1}H, ^{13}C NMR and data HPLC-HRMS-ESI spectra of (*Z*)-6-(4-chlorophenyl)-8-fluoro-1-(2-(4-(4-fluorophenyl)thiazol-2-yl)hydrazineylidene)-4,4,6-trimethyl-5,6-dihydro-4*H*-pyrrolo[3,2,1-*ij*]quinolin-2(1*H*)-one 5l S32

^{1}H, ^{13}C NMR and data HPLC-HRMS-ESI spectra of (*Z*)-6-(4-chlorophenyl)-1-(2-(4-(3-chlorophenyl)thiazol-2-yl)hydrazineylidene)-8-fluoro-4,4,6-trimethyl-5,6-dihydro-4*H*-pyrrolo[3,2,1-*ij*]quinolin-2(1*H*)-one 5m S34

^{1}H, ^{13}C NMR and data HPLC-HRMS-ESI spectra of (*Z*)-6-(4-chlorophenyl)-8-fluoro-1-(2-(4-(3-methoxyphenyl)thiazol-2-yl)hydrazineylidene)-4,4,6-trimethyl-5,6-dihydro-4*H*-pyrrolo[3,2,1-*ij*]quinolin-2(1*H*)-one 5n S35

^{1}H, ^{13}C NMR and data HPLC-HRMS-ESI spectra of (*Z*)-8-bromo-6-(4-chlorophenyl)-4,4,6-trimethyl-1-(2-(4-phenylthiazol-2-yl)hydrazineylidene)-5,6-dihydro-4*H*-pyrrolo[3,2,1-*ij*]quinolin-2(1*H*)-one 5o S37

The dependence of inhibition of factor Xa- and XIa-induced chromogenic substrate hydrolysis on the compound concentration. S38

The dependence of inhibition of factor Xa- and XIa-induced chromogenic substrate hydrolysis on the concentration of 5d S38

The dependence of inhibition of factor Xa- and XIa-induced chromogenic substrate hydrolysis on the concentration of 5h S39

Crystal data and structure refinement parameters for 5d S40

Copies of spectral data of synthesized compounds

¹H, ¹³C NMR and data HPLC-HRMS-ESI spectra of (*Z*)-2-(8-chloro-4,4,6-trimethyl-2-oxo-5,6-dihydro-4*H*-pyrrolo[3,2,1-*ij*]quinolin-1(2*H*)-ylidene)hydrazine-1-carbothioamide 3b

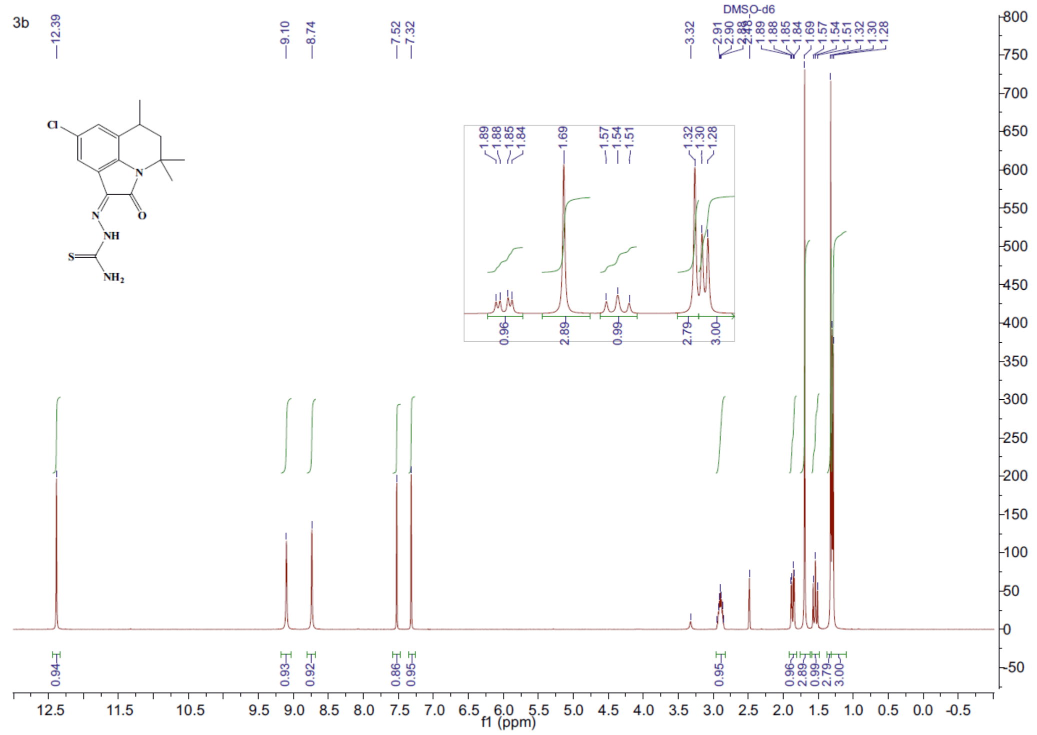

Figure S1. **^{1}H NMR (DMSO-d$_6$, 400 MHz) spectrum of compound 3b**

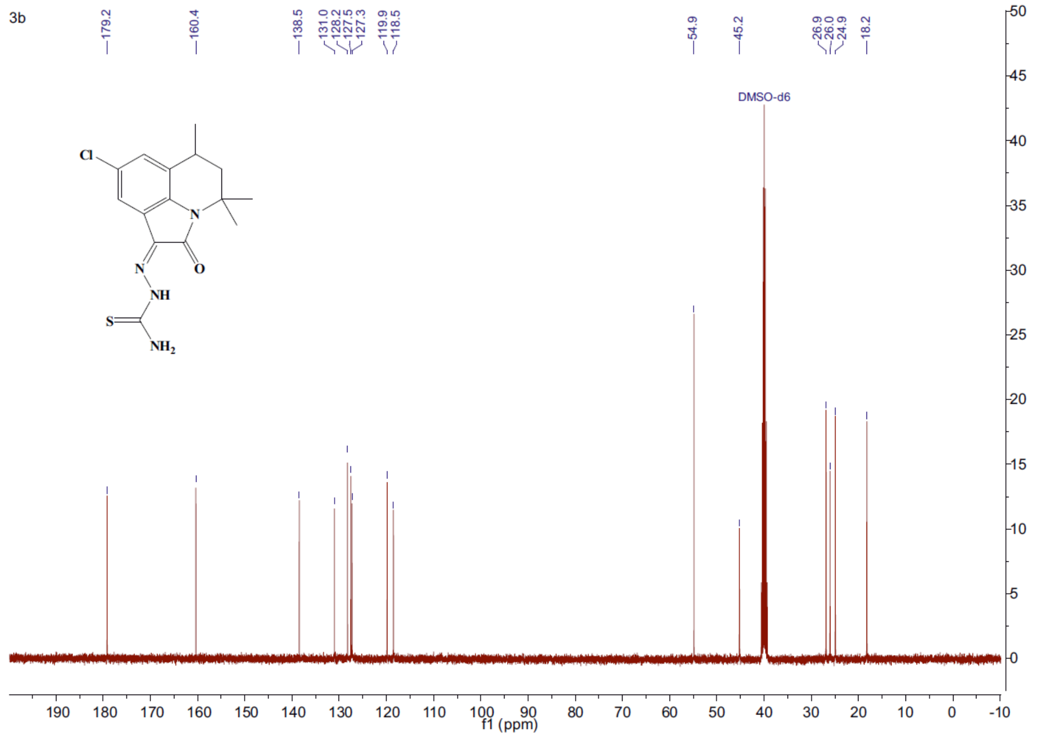

Figure S2. **^{13}C NMR (DMSO-d$_6$, 101 MHz) spectrum of compound 3b**

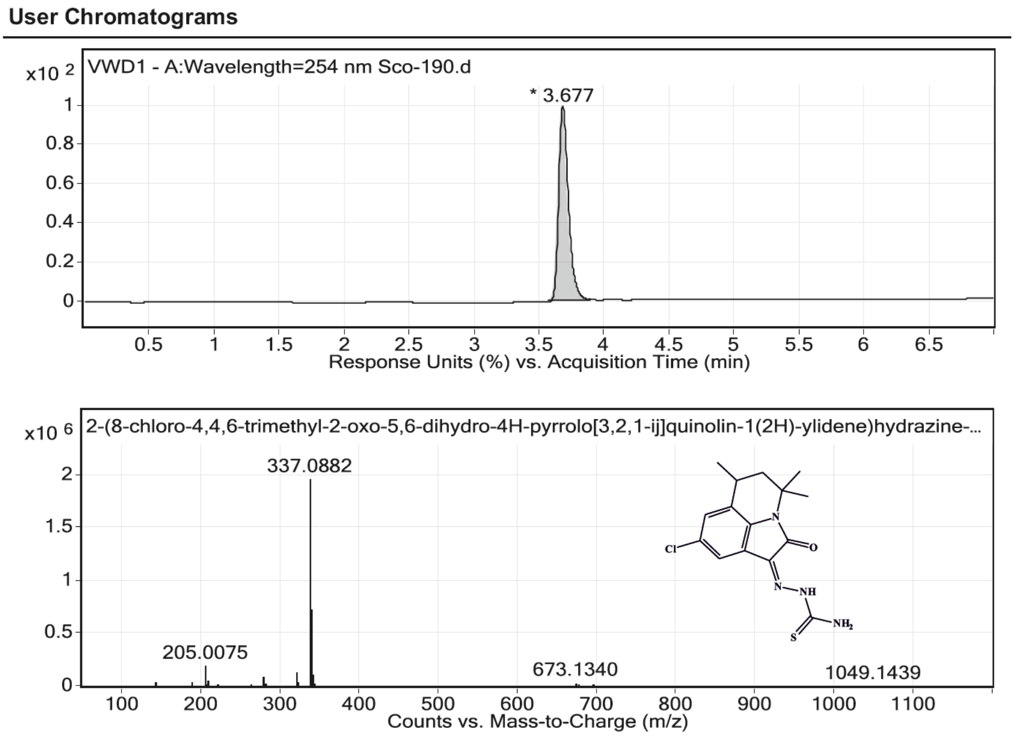

Figure S3. Data of HPLC-MS-ESI analysis of 3b

^{1}H, ^{13}C NMR and data HPLC-HRMS-ESI spectra of (*Z*)-2-(8-fluoro-4,4,6-trimethyl-2-oxo-5,6-dihydro-4*H*-pyrrolo[3,2,1-*ij*]quinolin-1(2*H*)-ylidene)hydrazine-1-carbothioamide 3c

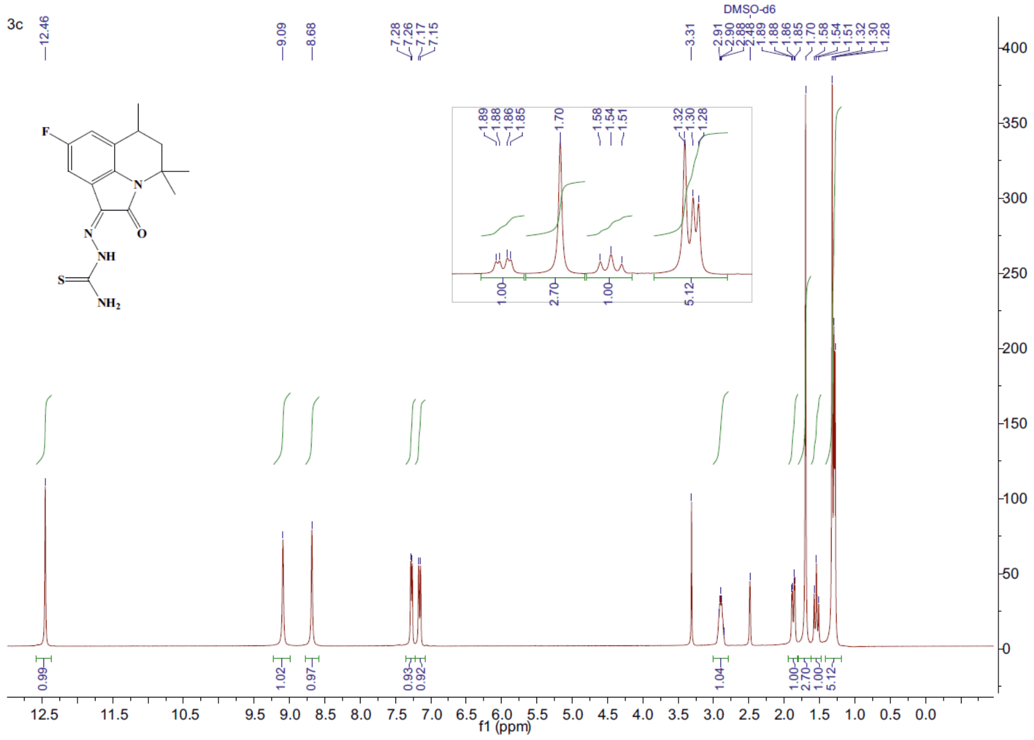

Figure S4. ^{1}H NMR (DMSO-d$_6$, 400 MHz) spectrum of compound 3c

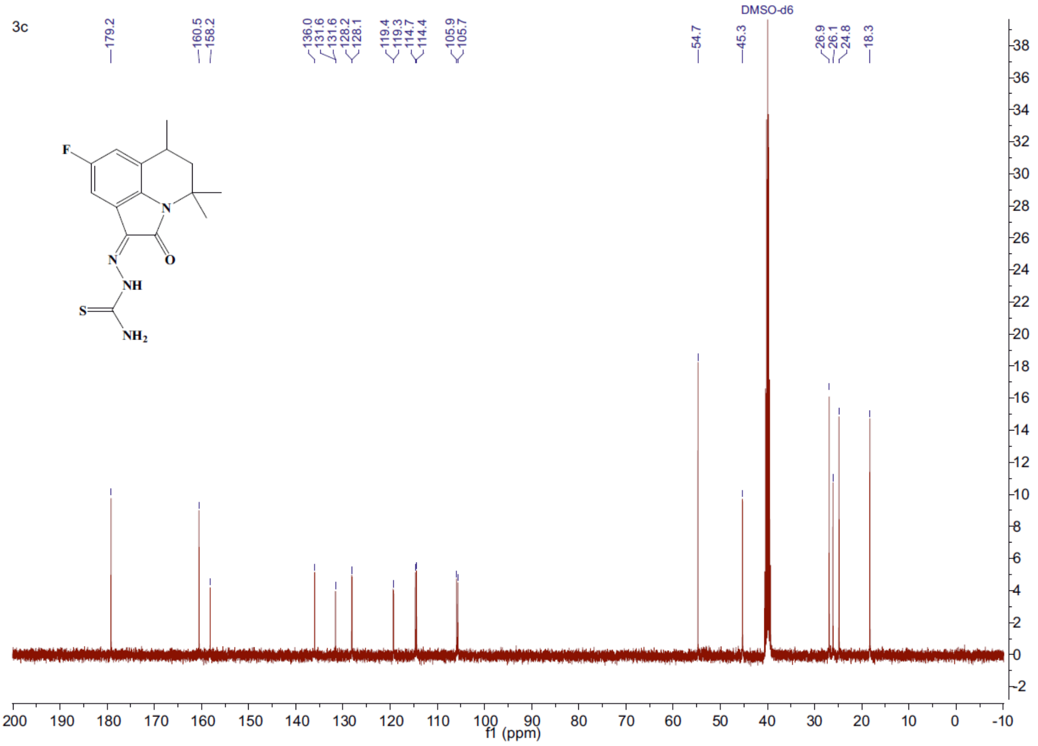

Figure S5. ^{13}**C NMR (DMSO-d**$_6$**, 101 MHz) spectrum of compound 3c**

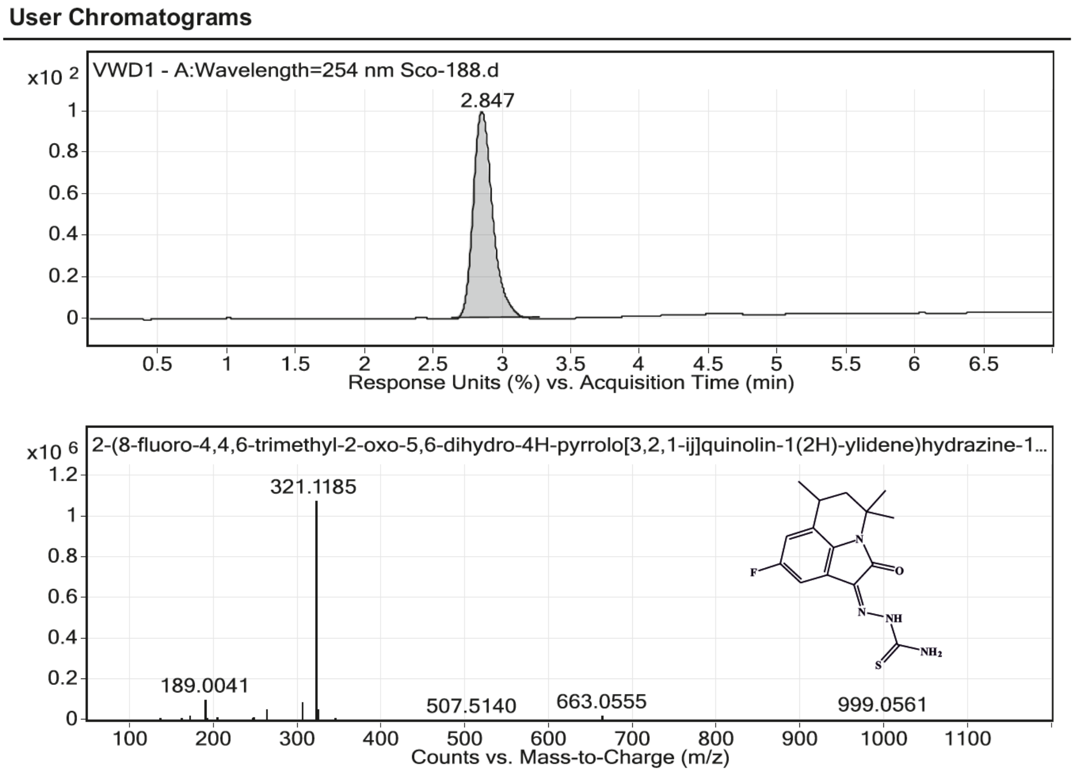

Figure S6. Data of HPLC-MS-ESI analysis of 3c

^{1}H, ^{13}C NMR and data HPLC-HRMS-ESI spectra of (*Z*)-2-(8-fluoro-4,4,6-trimethyl-2-oxo-6-phenyl-5,6-dihydro-4*H*-pyrrolo[3,2,1-*ij*]quinolin-1(2*H*)-ylidene)hydrazine-1-carbothioamide 3d

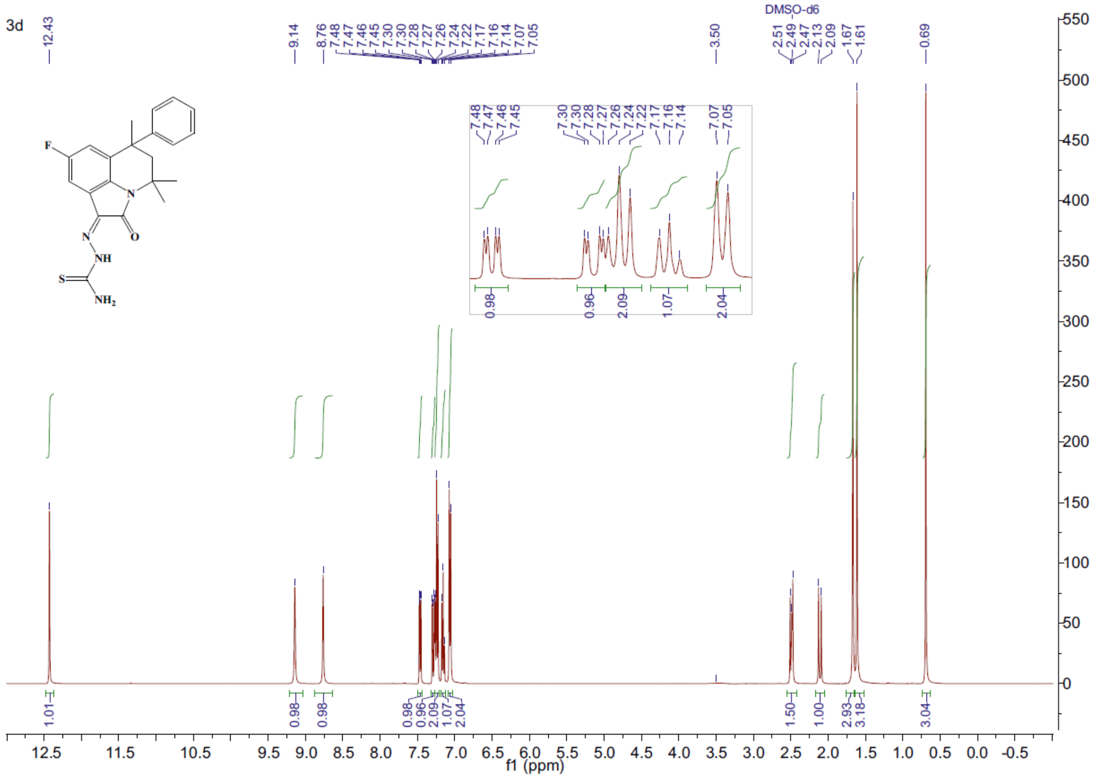

Figure S7. ^{1}H NMR (DMSO-d₆, 400 MHz) spectrum of compound 3d

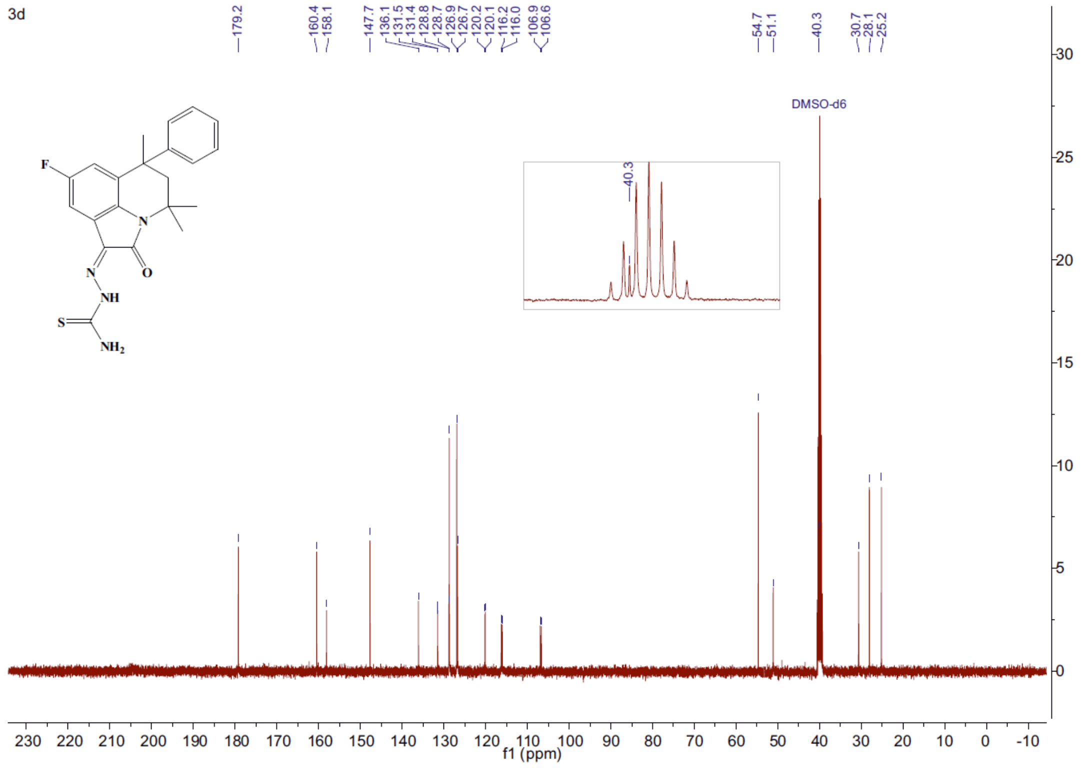

Figure S8. ^{13}C NMR (DMSO-d₆, 101 MHz) spectrum of compound 3d

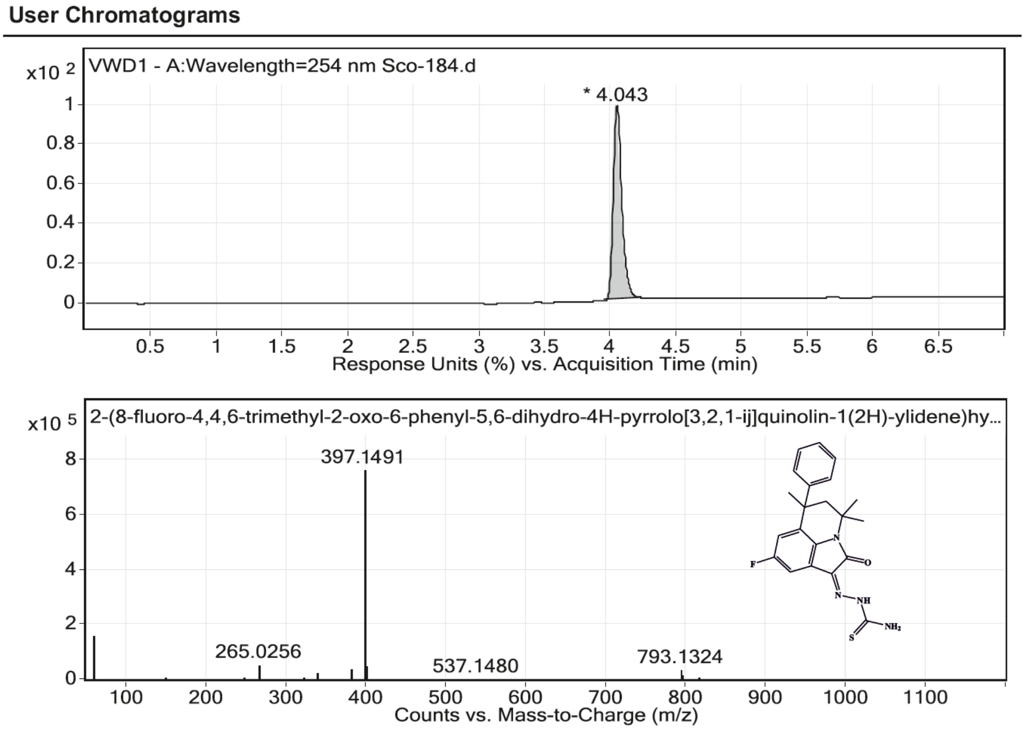

Figure S9. Data of HPLC-MS-ESI analysis of 3d

^{1}H, ^{13}C NMR and data HPLC-HRMS-ESI spectra of (*Z*)-2-(8-bromo-4,4,6-trimethyl-2-oxo-6-phenyl-5,6-dihydro-4*H*-pyrrolo[3,2,1-*ij*]quinolin-1(2*H*)-ylidene)hydrazine-1-carbothioamide 3e

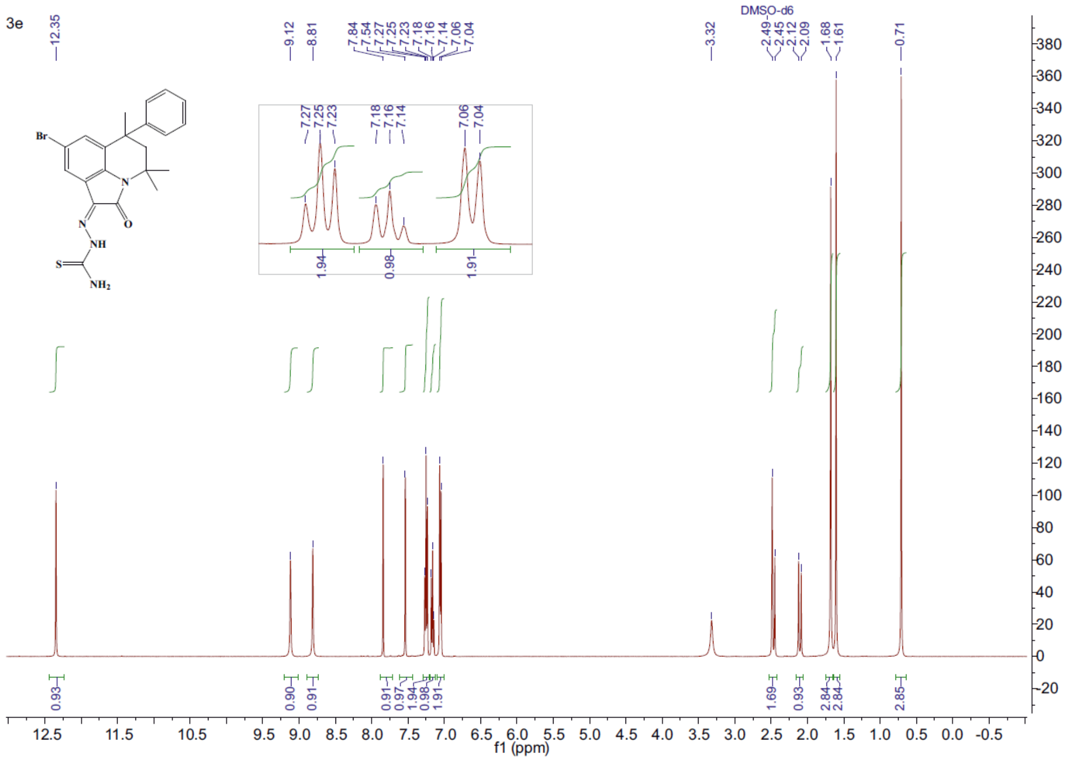

Figure S10. ^{1}H NMR (DMSO-d_6, 400 MHz) spectrum of compound 3e

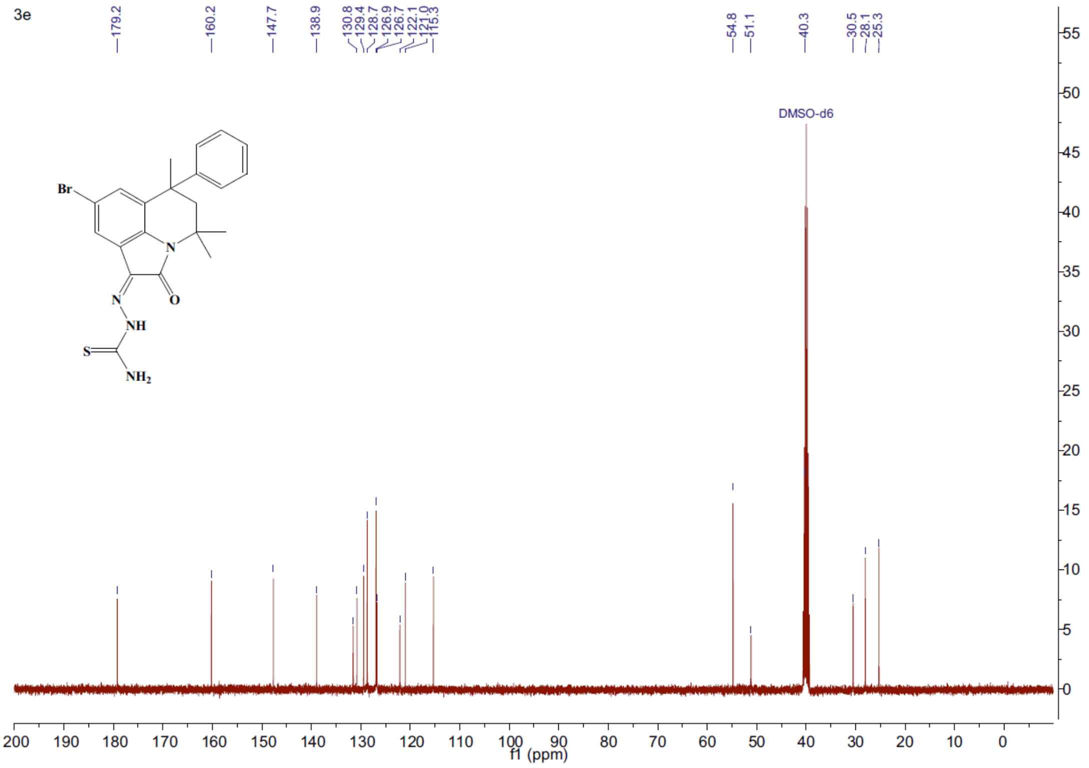

Figure S11. ^{13}**C NMR (DMSO-d**$_6$**, 101 MHz) spectrum of compound 3e**

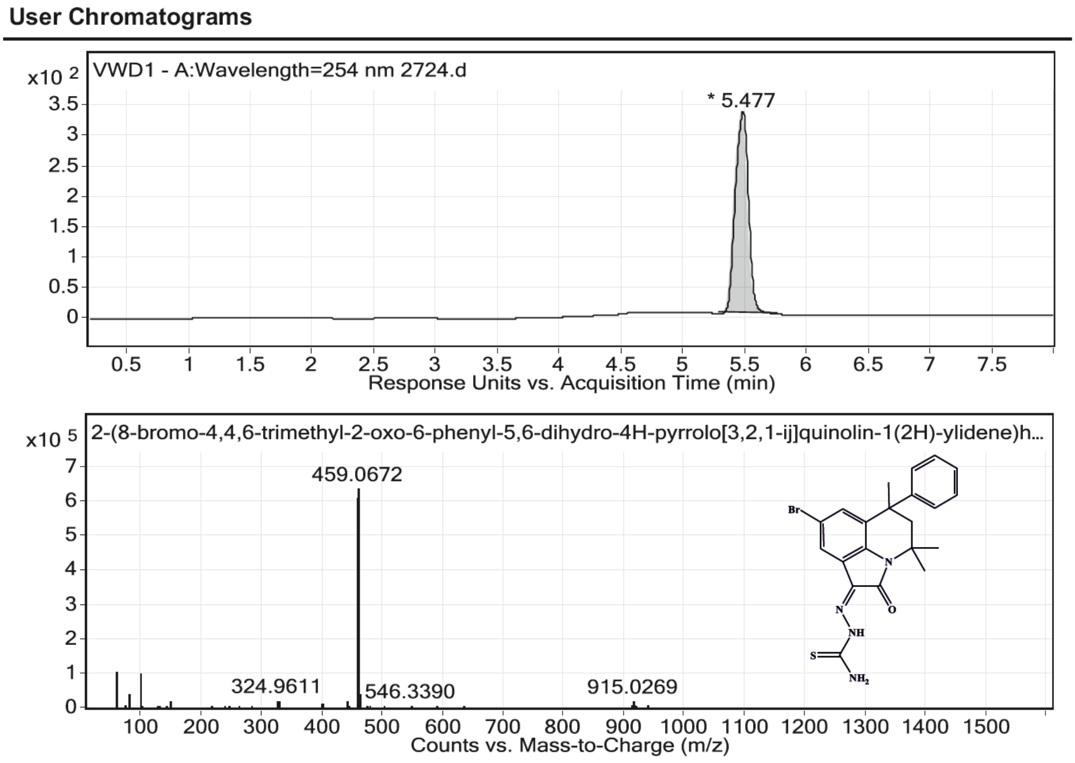

Figure S12. Data of HPLC-MS-ESI analysis of 3e

^{1}H, ^{13}C NMR and data HPLC-HRMS-ESI spectra of (*Z*)-2-(6-(4-chlorophenyl)-4,4,6-trimethyl-2-oxo-5,6-dihydro-4*H*-pyrrolo[3,2,1-*ij*]quinolin-1(2*H*)-ylidene)hydrazine-1-carbothioamide 3f

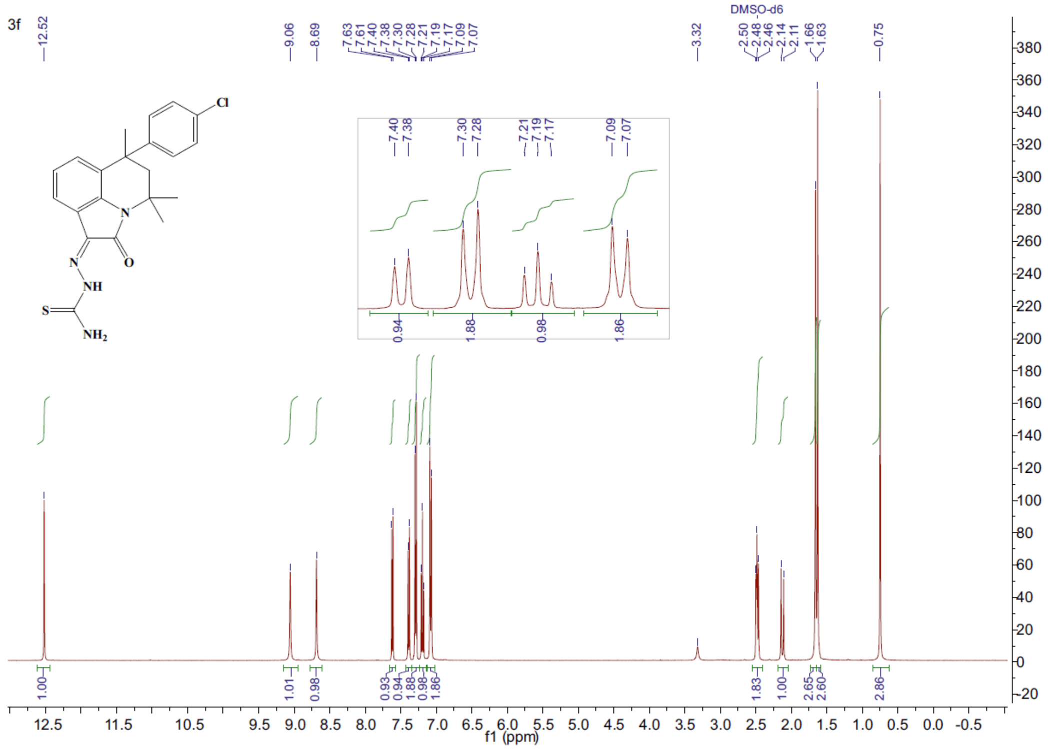

Figure S13. ^{1}H NMR (DMSO-d_6, 400 MHz) spectrum of compound 3f

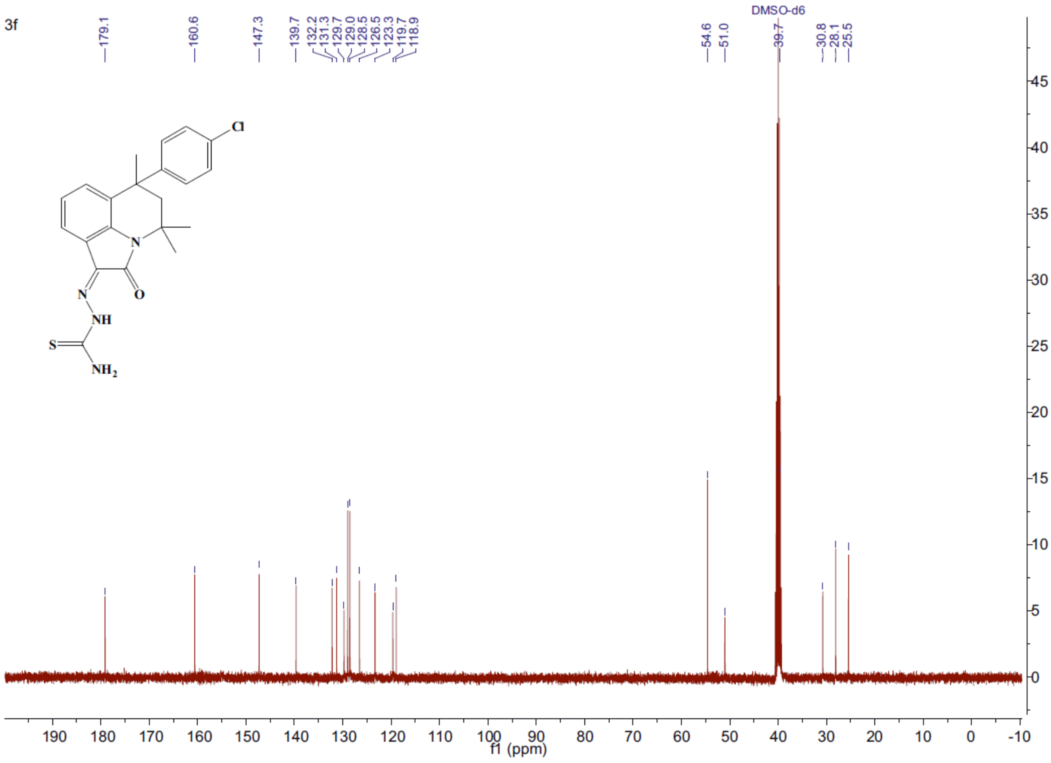

Figure S14. ^{13}C NMR (DMSO-d_6, 101 MHz) spectrum of compound 3f

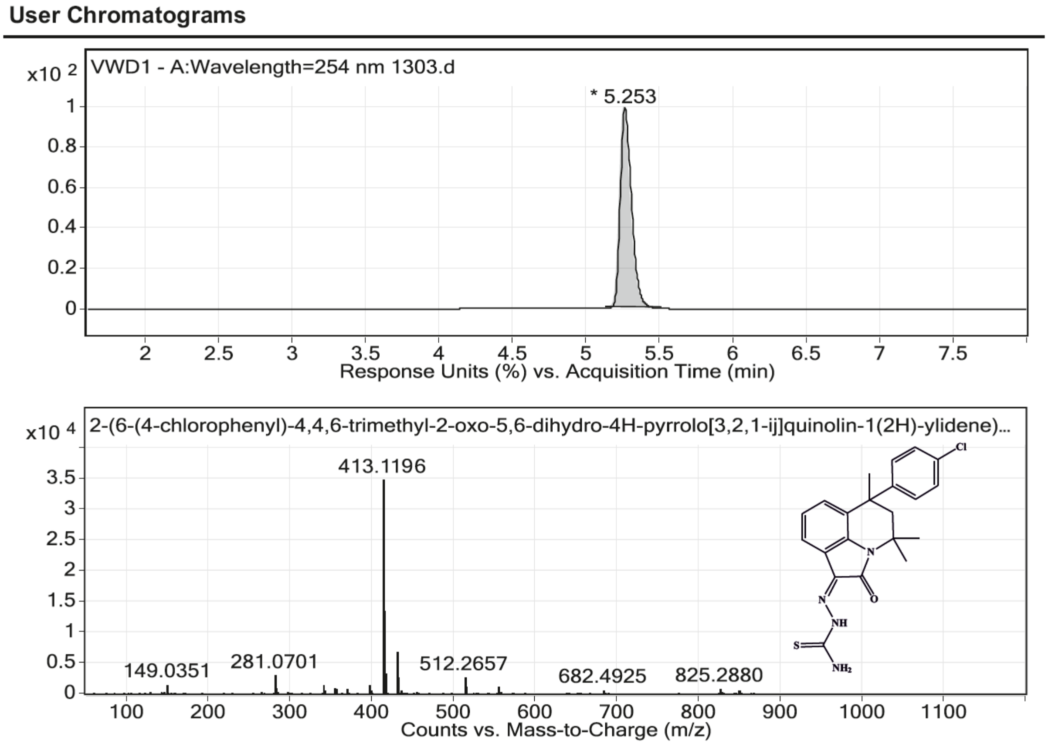

Figure S15. Data of HPLC-MS-ESI analysis of 3f

^{1}H, ^{13}C NMR and data HPLC-HRMS-ESI spectra of (*Z*)-2-(8-chloro-6-(4-chlorophenyl)-4,4,6-trimethyl-2-oxo-5,6-dihydro-4*H*-pyrrolo[3,2,1-*ij*]quinolin-1(2*H*)-ylidene)hydrazine-1-carbothioamide 3g

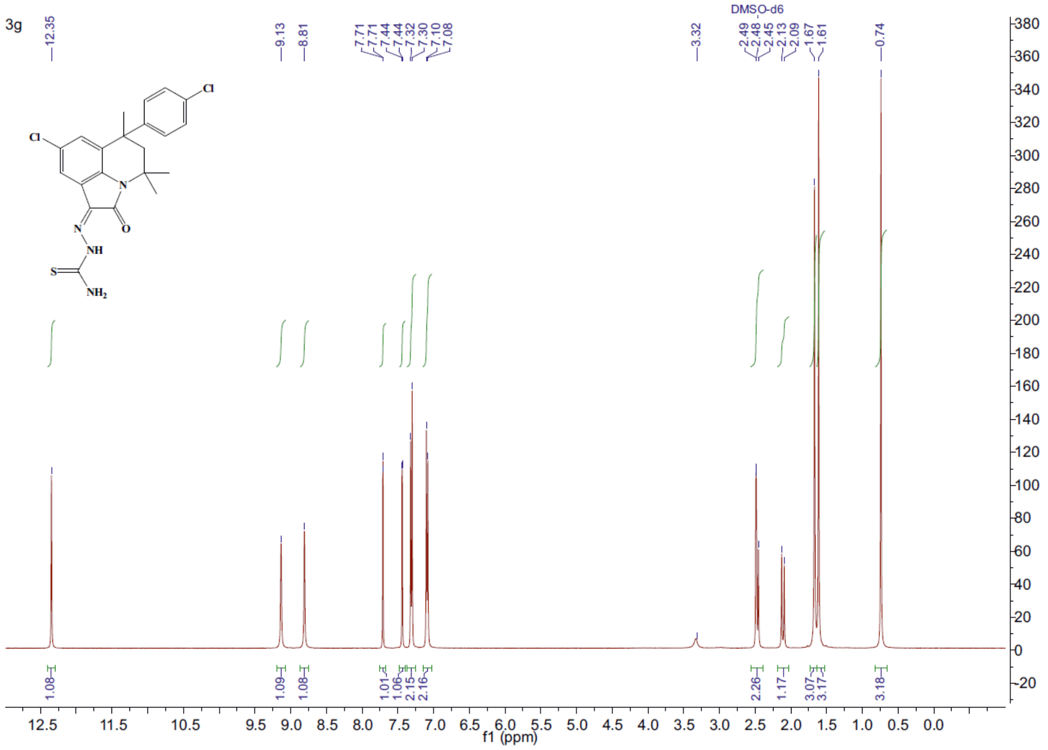

Figure S16. ^{1}H NMR (DMSO-d$_6$, 400 MHz) spectrum of compound 3g

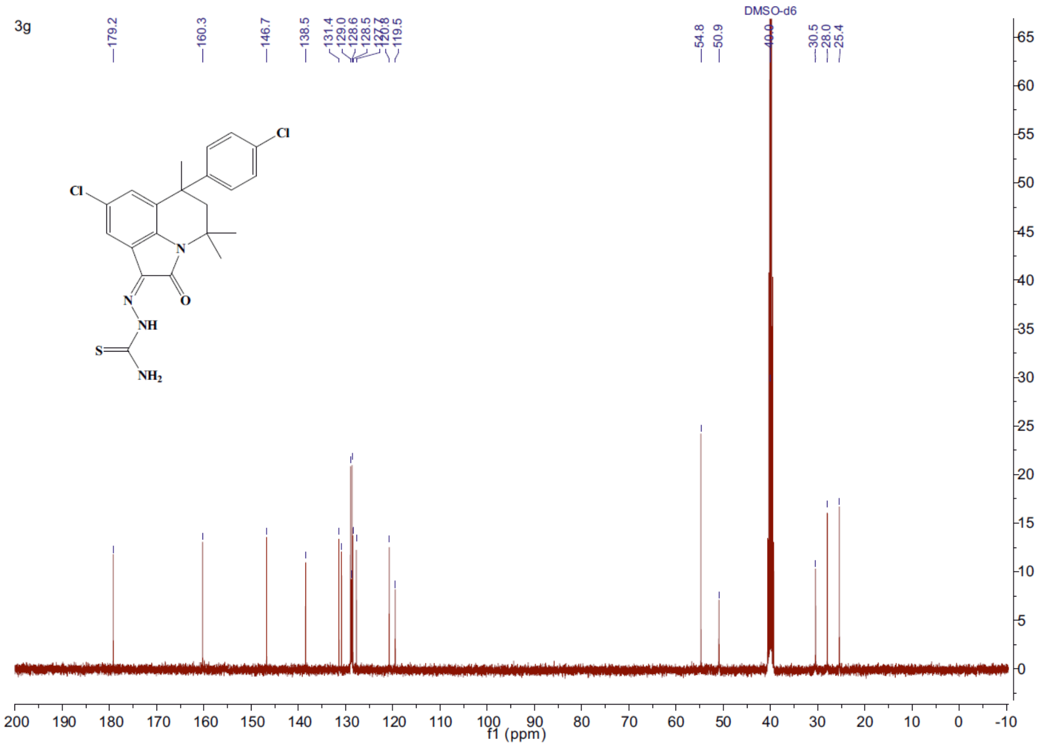

Figure S17. ^{13}C NMR (DMSO-d_6, 101 MHz) spectrum of compound 3g

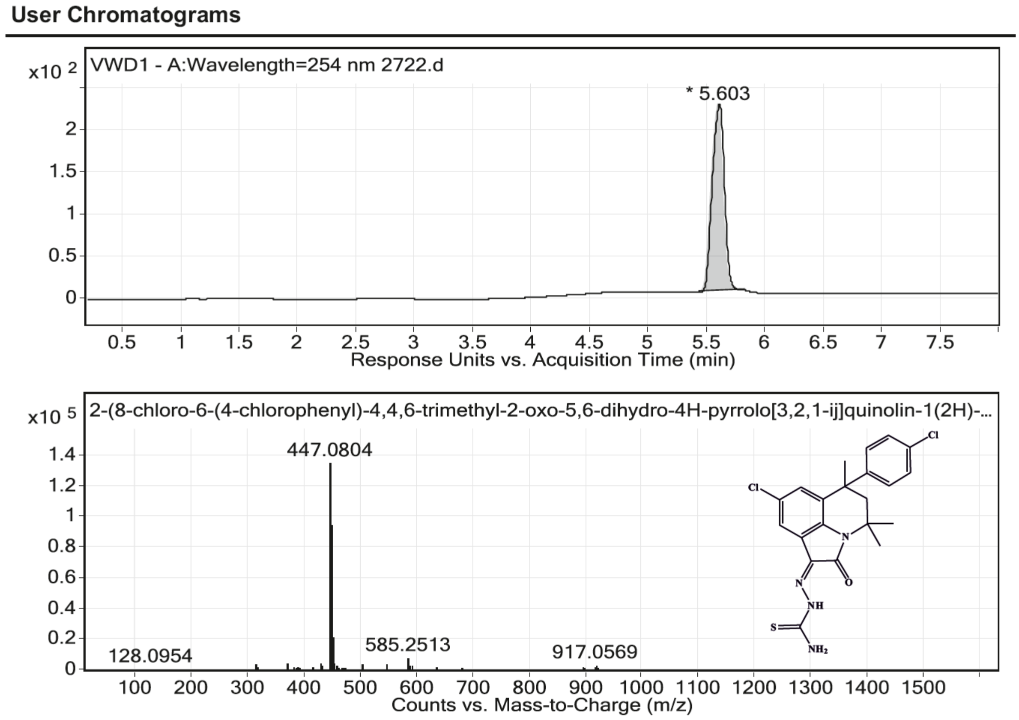

Figure S18. Data of HPLC-MS-ESI analysis of 3g

^{1}H, ^{13}C NMR and data HPLC-HRMS-ESI spectra of (*Z*)-2-(6-(4-chlorophenyl)-8-fluoro-4,4,6-trimethyl-2-oxo-5,6-dihydro-4*H*-pyrrolo[3,2,1-*ij*]quinolin-1(2*H*)-ylidene)hydrazine-1-carbothioamide 3h

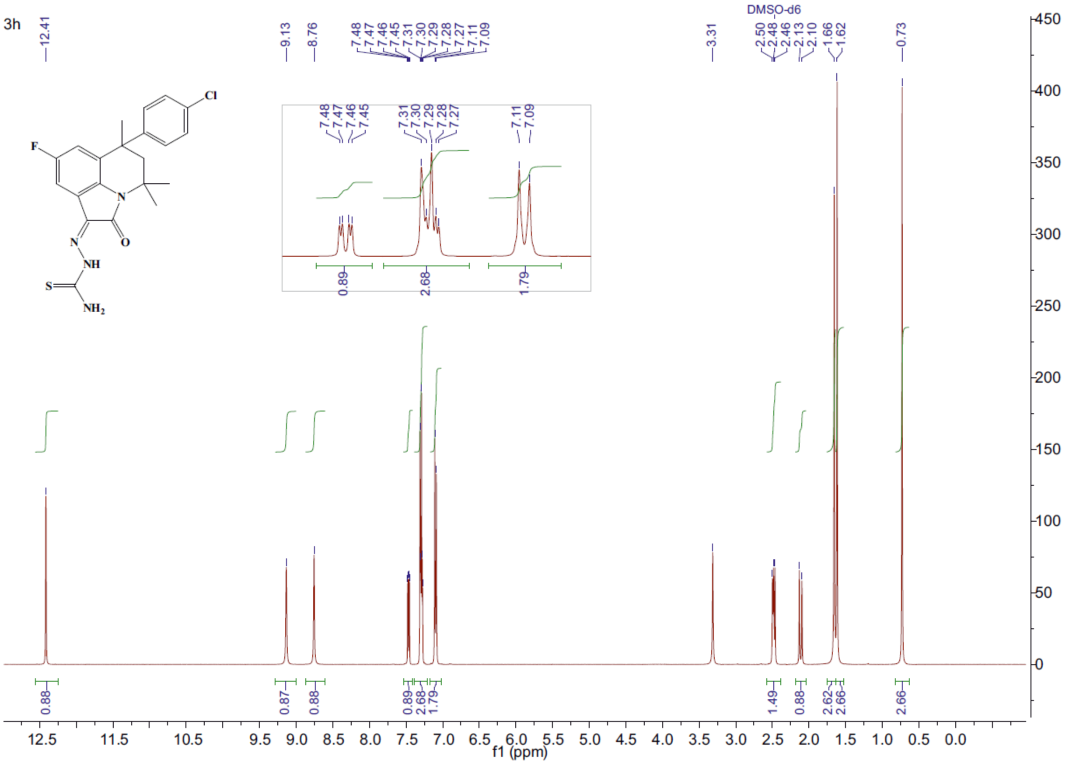

Figure S19. ^{1}H NMR (DMSO-d_6, 400 MHz) spectrum of compound 3h

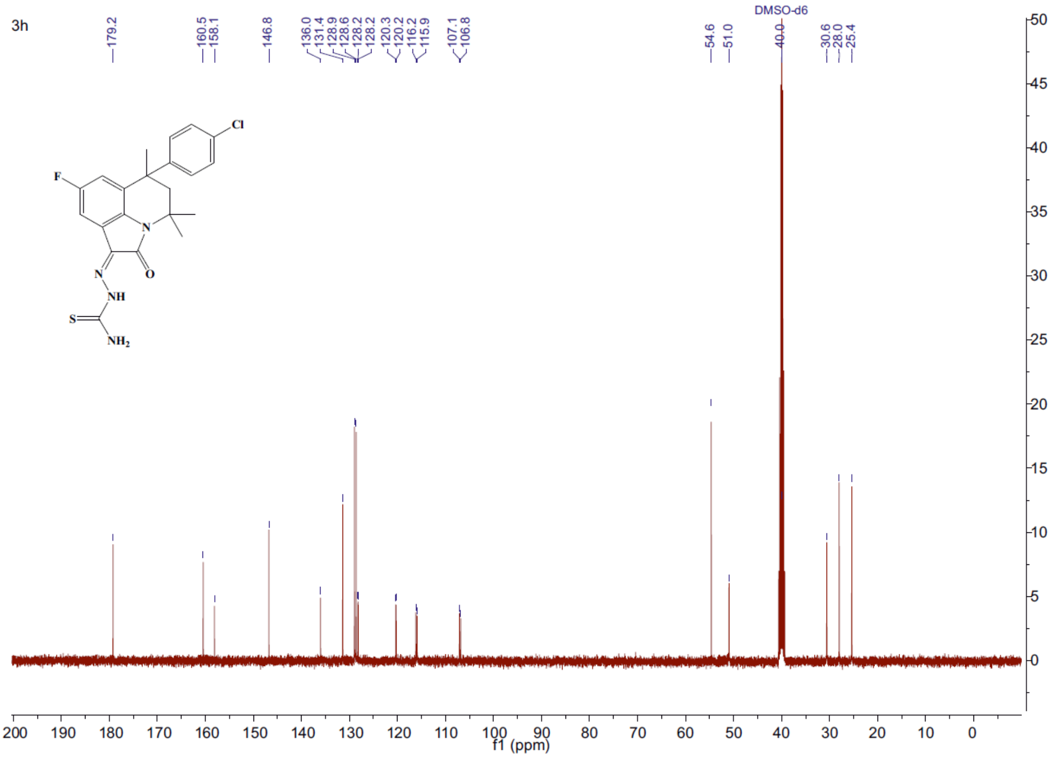

Figure S20. ^{13}C NMR (DMSO-d_6, 101 MHz) spectrum of compound 3h

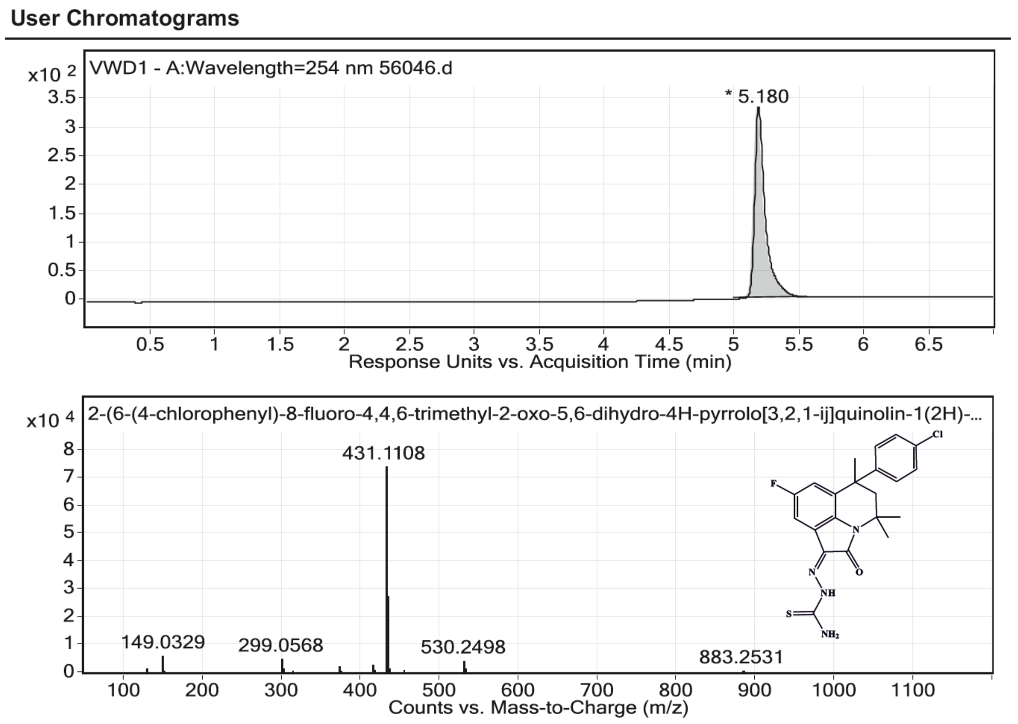

Figure S21. Data of HPLC-MS-ESI analysis of 3h

^{1}H, ^{13}C NMR and data HPLC-HRMS-ESI spectra of (*Z*)-2-(8-bromo-6-(4-chlorophenyl)-4,4,6-trimethyl-2-oxo-5,6-dihydro-4*H*-pyrrolo[3,2,1-*ij*]quinolin-1(2*H*)-ylidene)hydrazine-1-carbothioamide 3i

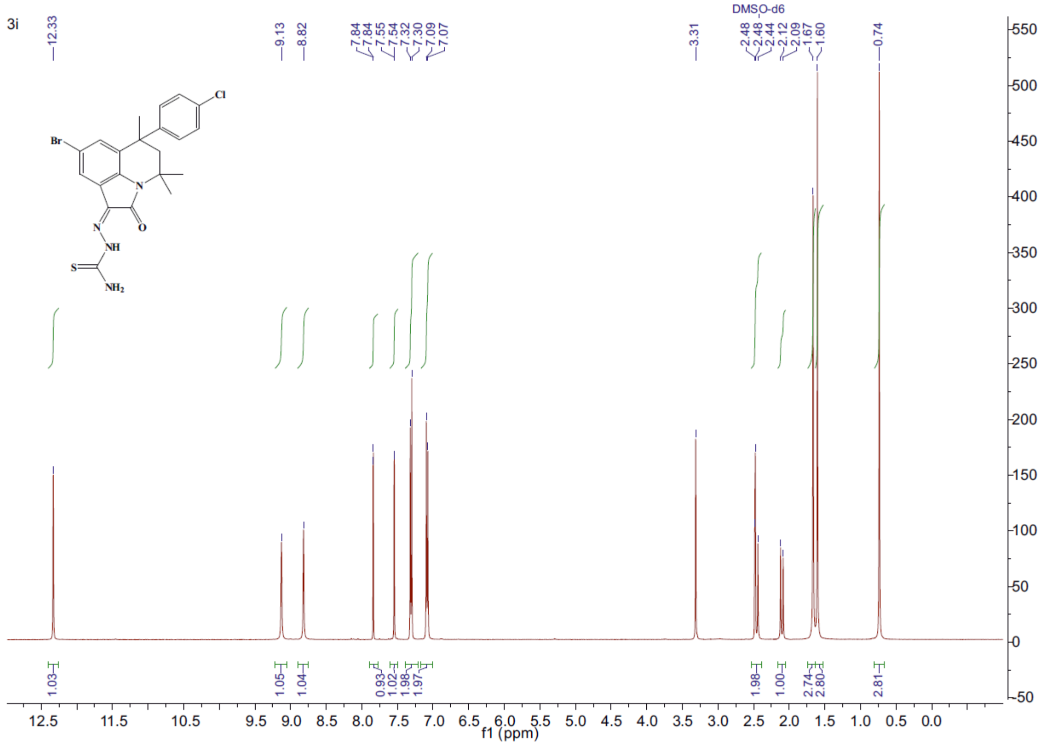

Figure S22. ^{1}H NMR (DMSO-d$_6$, 400 MHz) spectrum of compound 3i

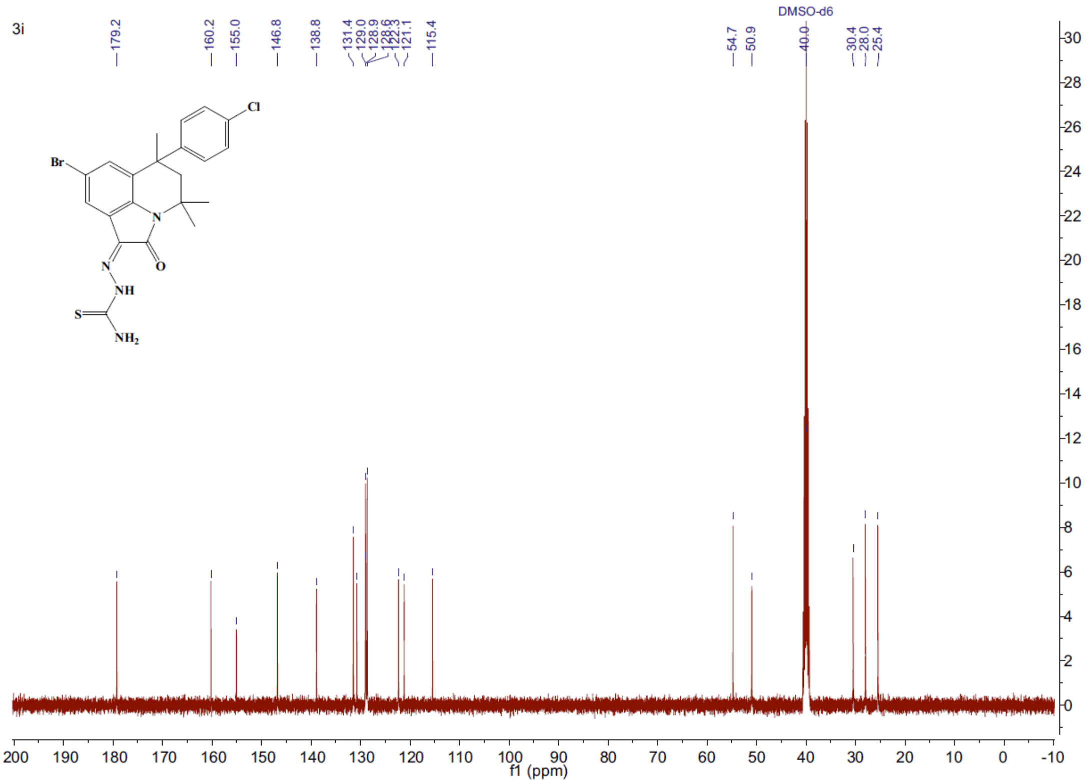

Figure S23. ^{13}C **NMR (DMSO-**d_6**, 101 MHz) spectrum of compound 3i**

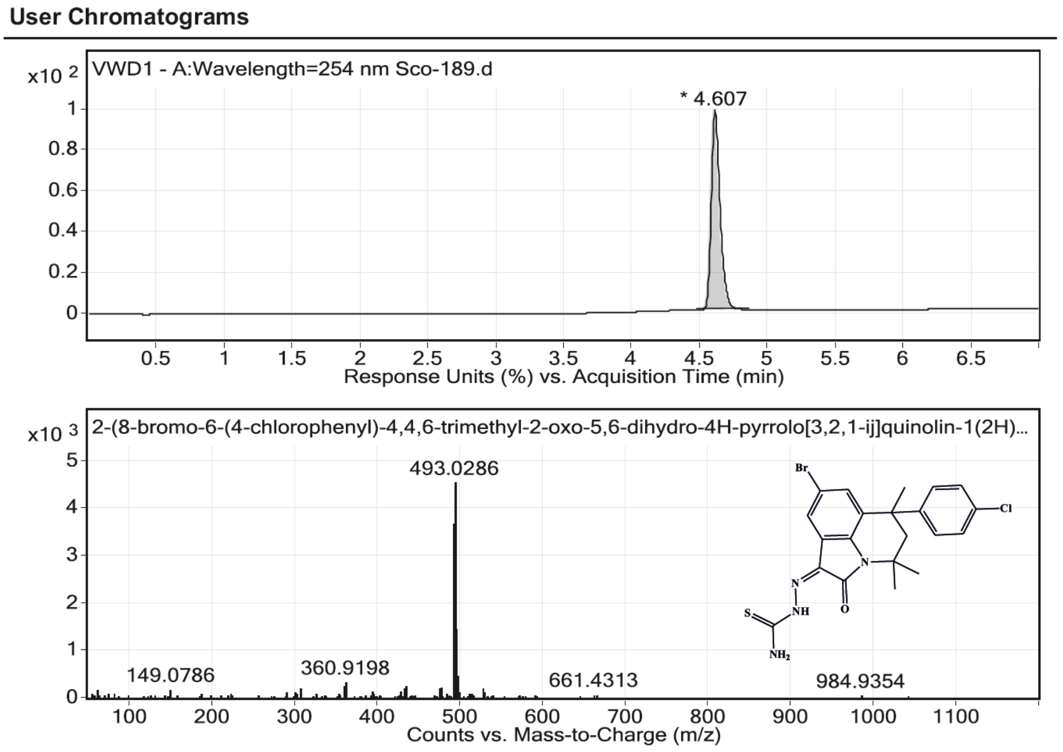

Figure S24. Data of HPLC-MS-ESI analysis of 3i

^{1}H, ^{13}C NMR and data HPLC-HRMS-ESI spectra of (*Z*)-1-(2-(4-(4-chlorophenyl)thiazol-2-yl)hydrazineylidene)-8-methoxy-4,4,6-trimethyl-5,6-dihydro-4*H*-pyrrolo[3,2,1-*ij*]quinolin-2(1*H*)-one 5a

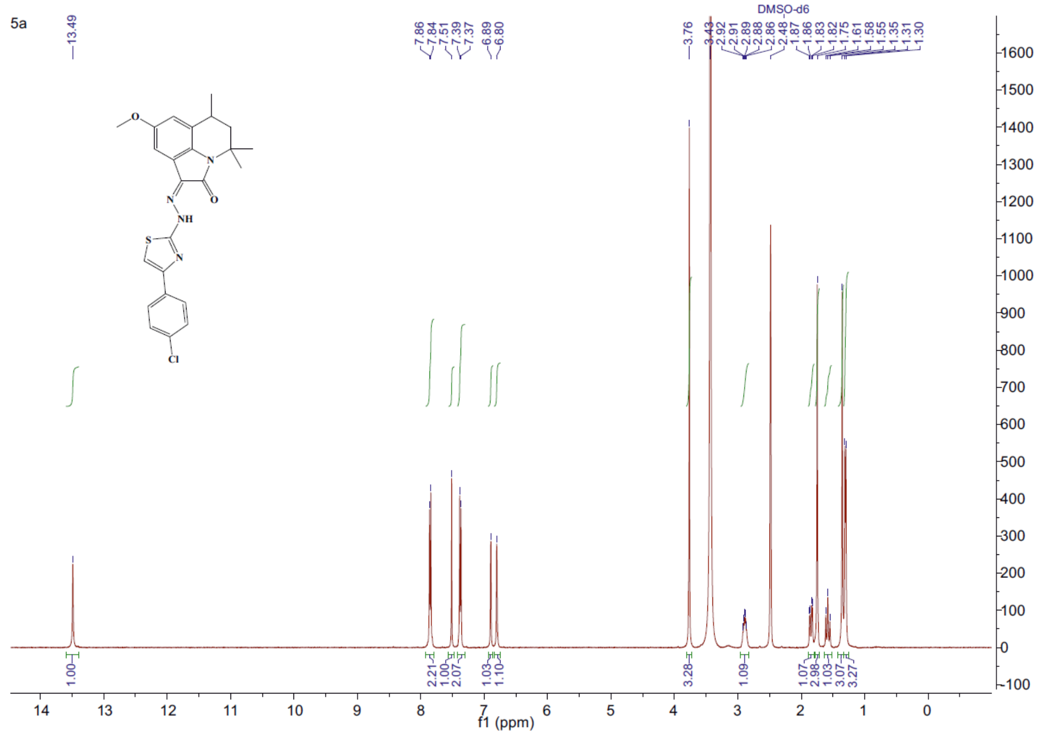

Figure S25. ^{1}H NMR (DMSO-d$_6$, 400 MHz) spectrum of compound 5a

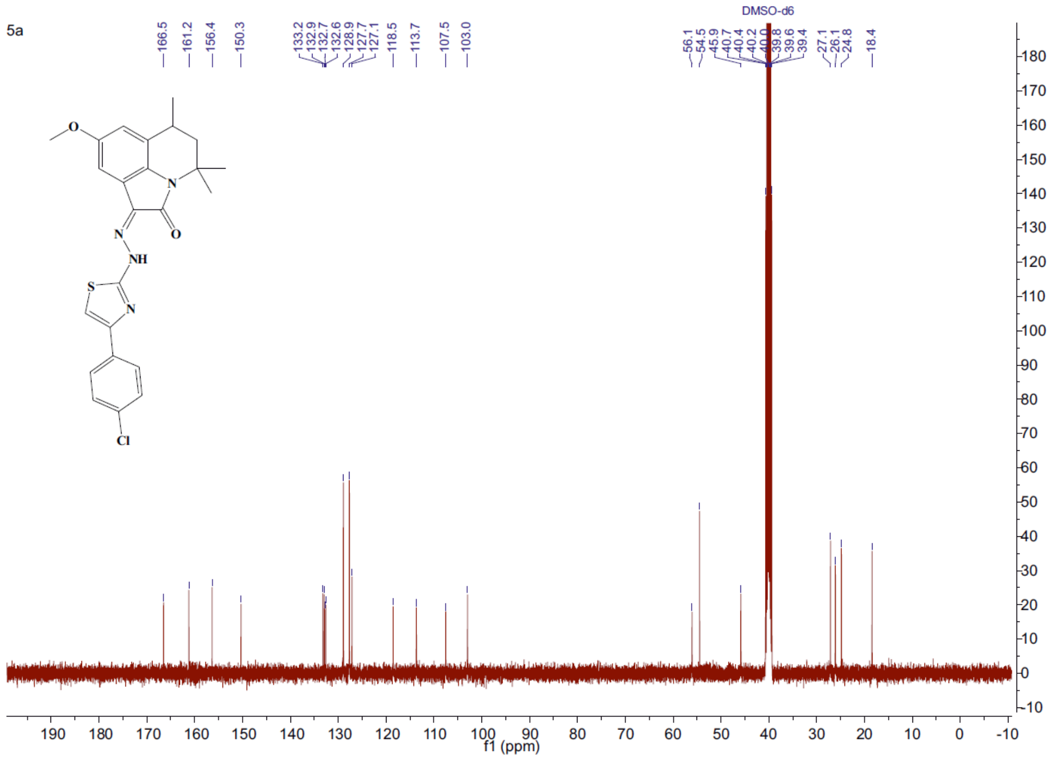

Figure S26. ^{13}C NMR (DMSO-d$_6$, 101 MHz) spectrum of compound 5a

User Chromatograms

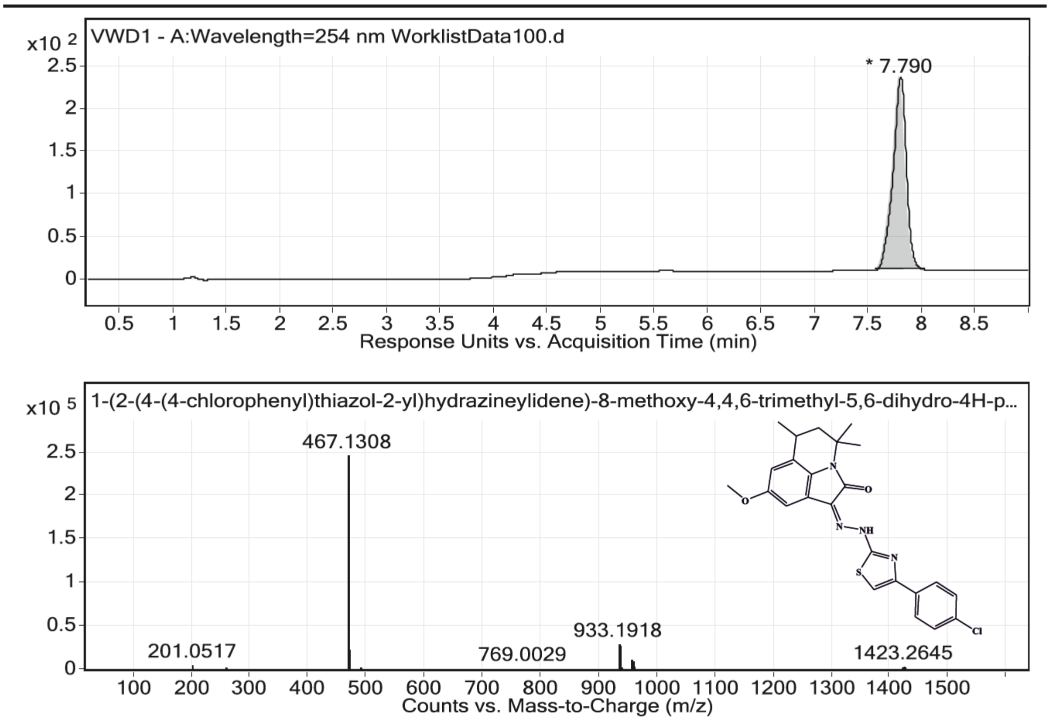

Figure S27. Data of HPLC-MS-ESI analysis of 5a

^{1}H, ^{13}C NMR and data HPLC-HRMS-ESI spectra of (*Z*)-1-(2-(4-(4-fluorophenyl)thiazol-2-yl)hydrazineylidene)-8-methoxy-4,4,6-trimethyl-5,6-dihydro-4*H*-pyrrolo[3,2,1-*ij*]quinolin-2(1*H*)-one 5b

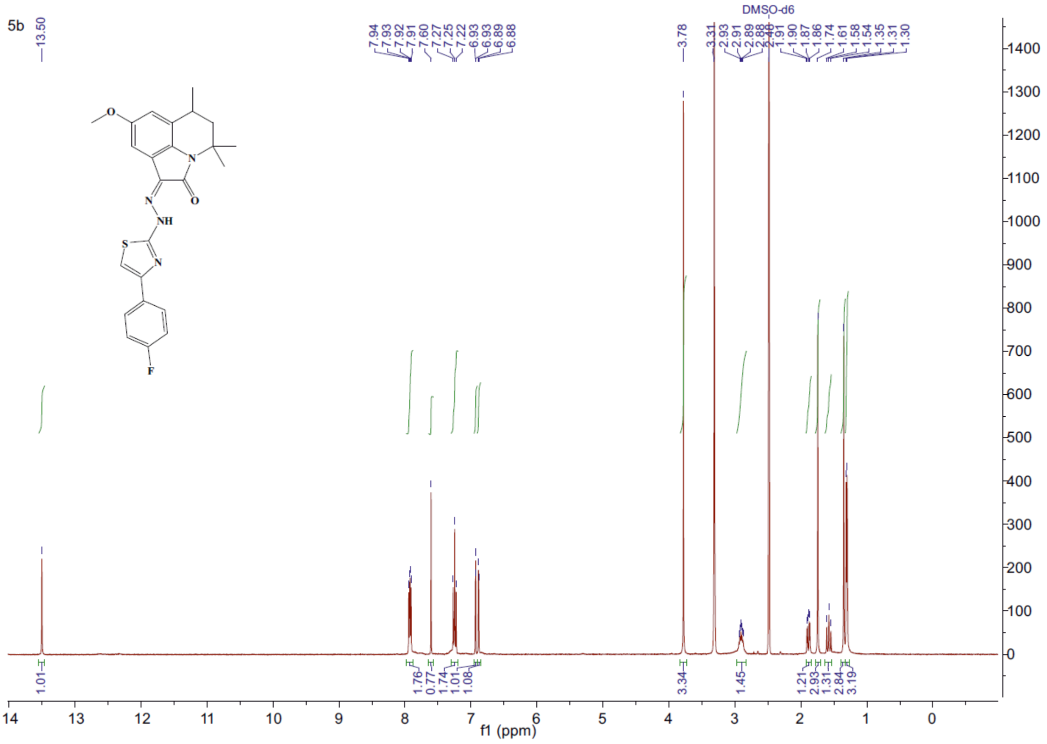

Figure S28. ^{1}H NMR (DMSO-d$_6$, 400 MHz) spectrum of compound 5b

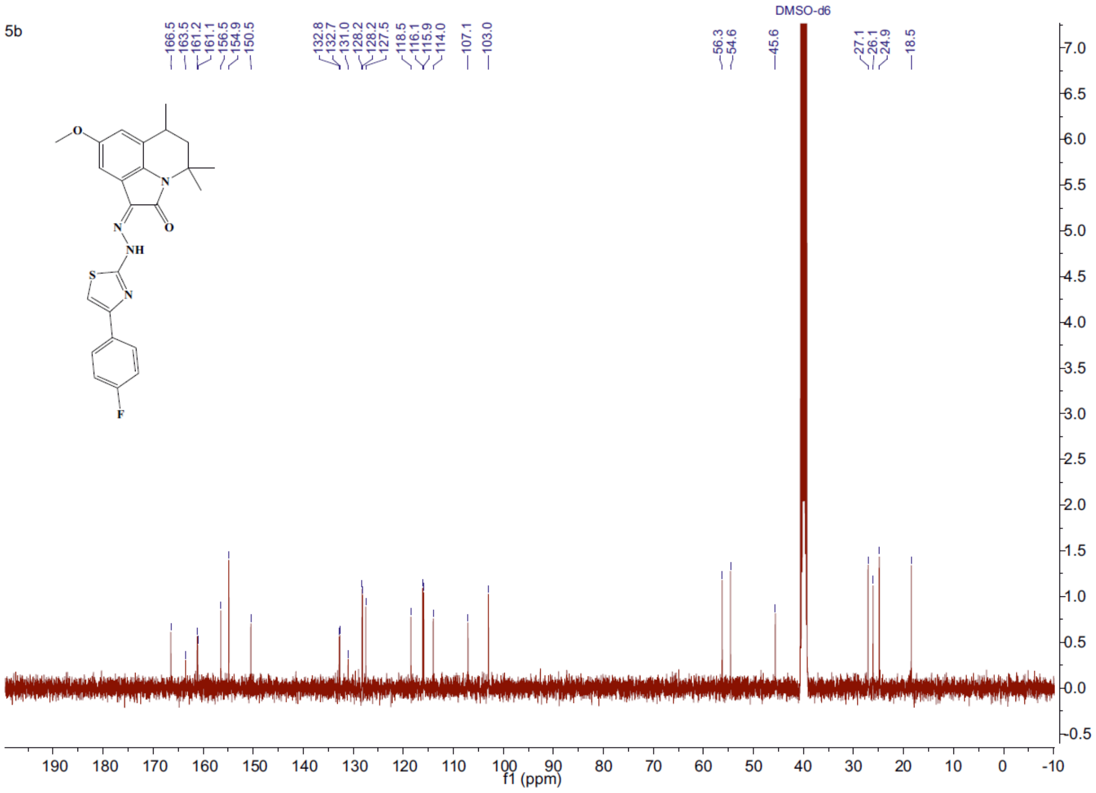

Figure S29. ^{13}C **NMR (DMSO-d$_6$, 101 MHz) spectrum of compound 5b**

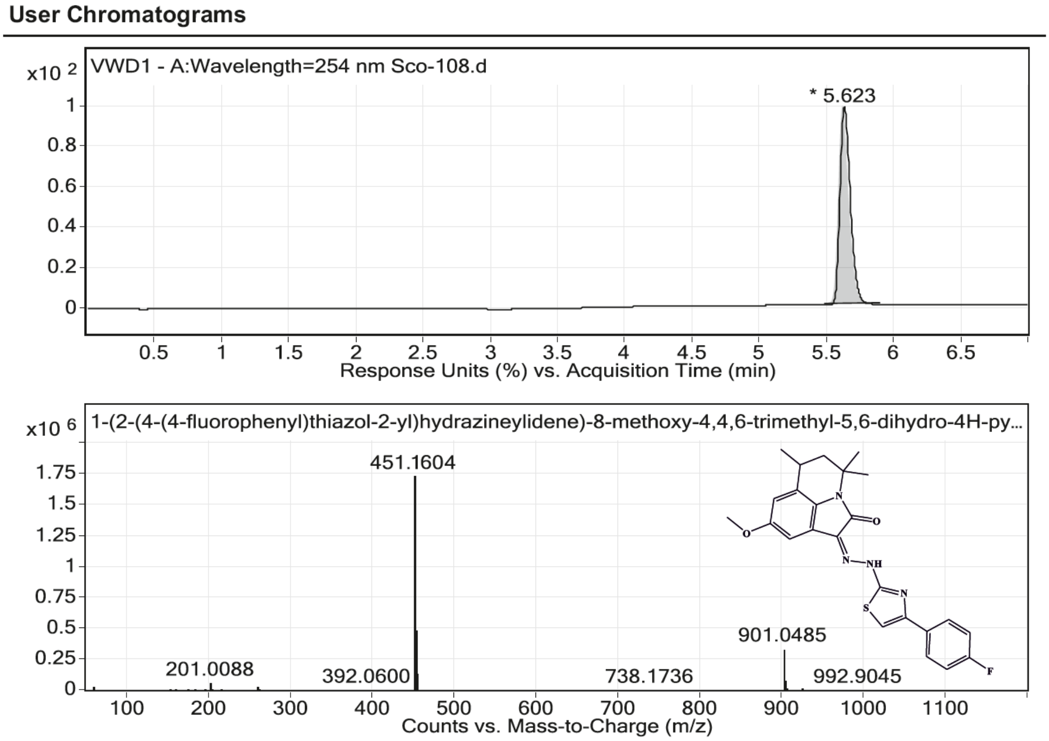

Figure S30. Data of HPLC-MS-ESI analysis of 5b

^{1}H, ^{13}C NMR and data HPLC-HRMS-ESI spectra of (*Z*)-1-(2-(4-(4-bromophenyl)thiazol-2-yl)hydrazineylidene)-8-methoxy-4,4,6-trimethyl-5,6-dihydro-4*H*-pyrrolo[3,2,1-*ij*]quinolin-2(1*H*)-one 5c

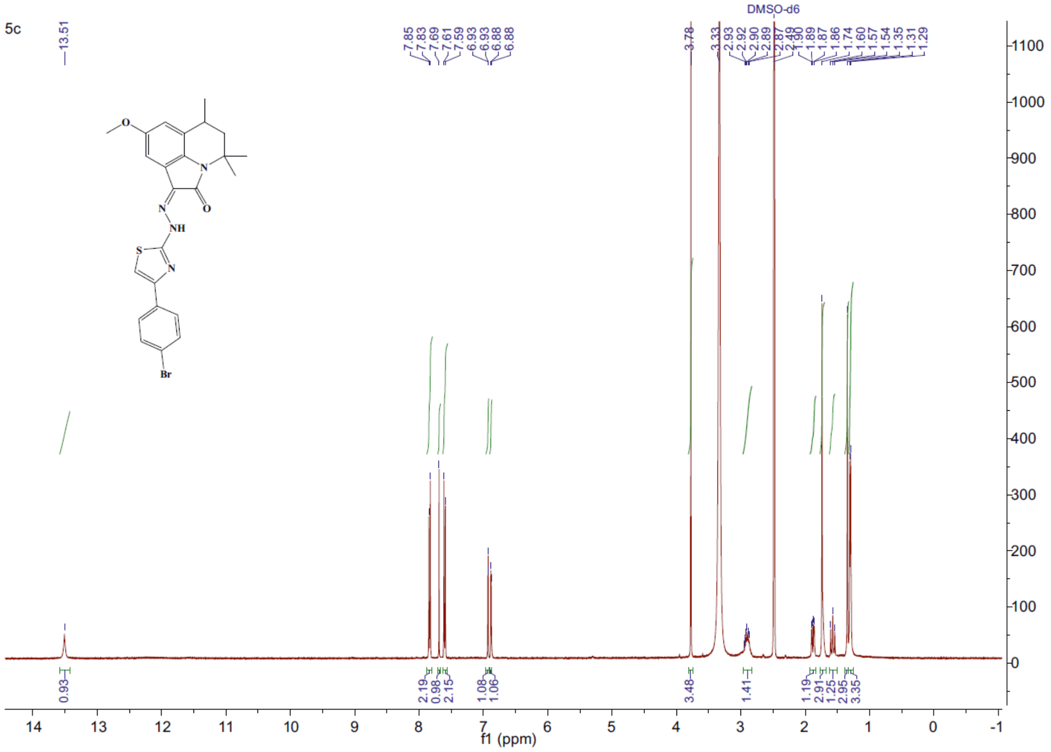

Figure S31. ^{1}H NMR (DMSO-d$_6$, 400 MHz) spectrum of compound 5c

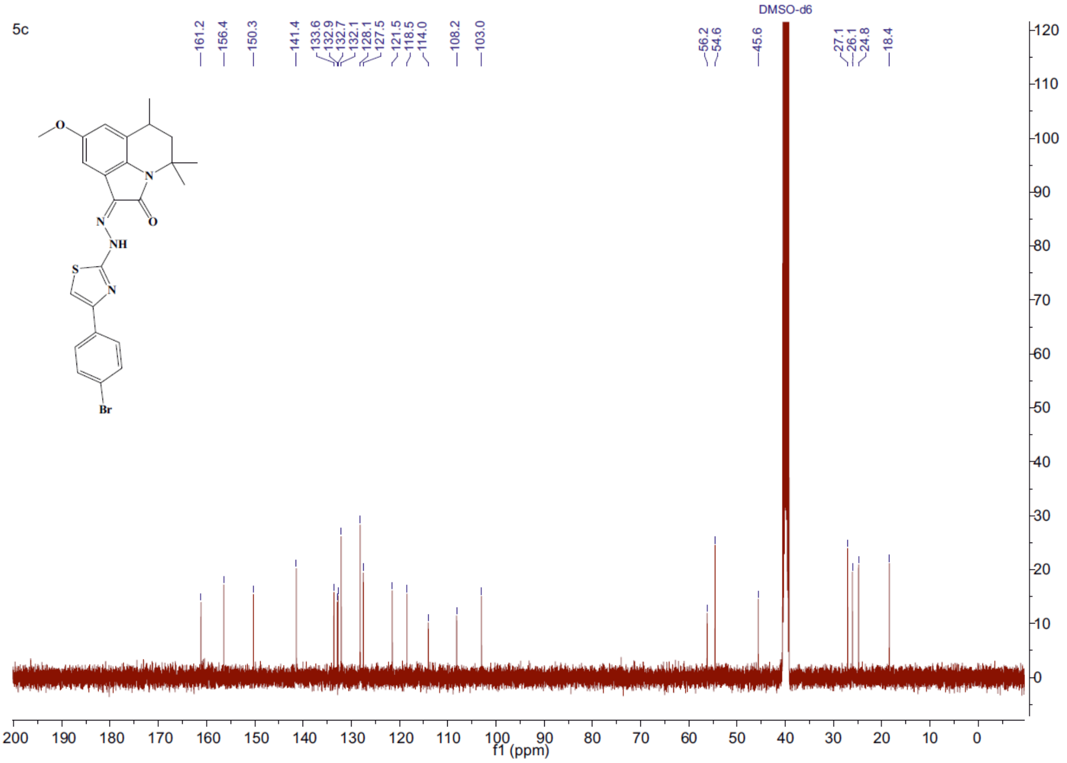

Figure S32. ^{13}C NMR (DMSO-d$_6$, 101 MHz) spectrum of compound 5c

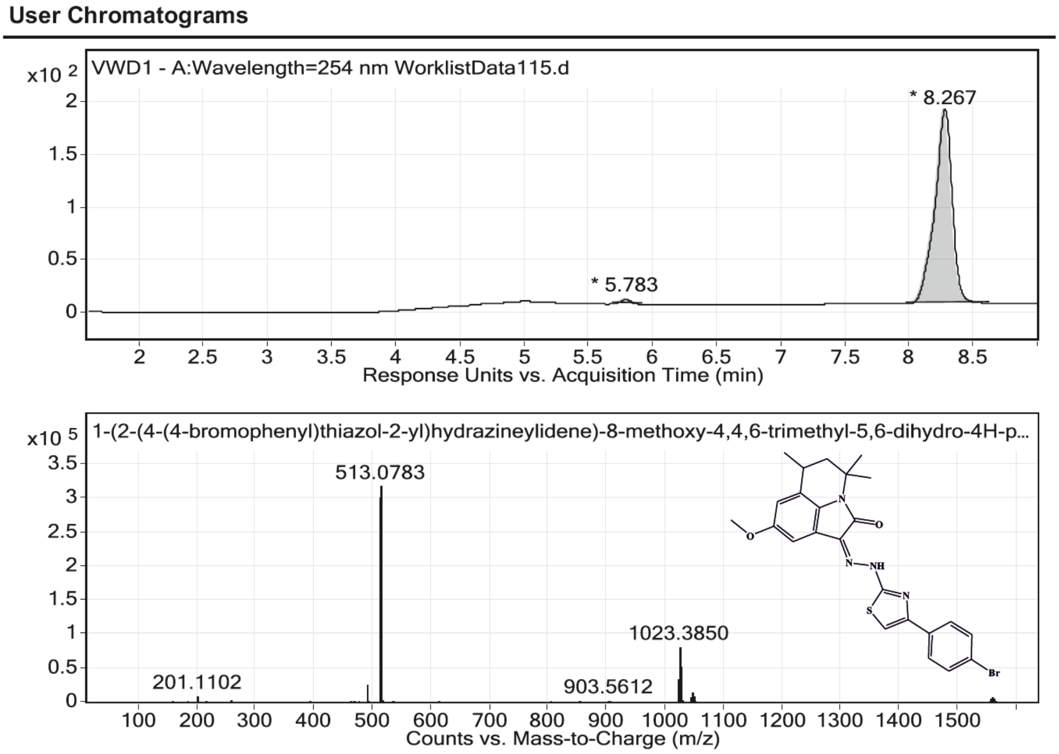

Figure S33. Data of HPLC-MS-ESI analysis of 5c

^{1}H, ^{13}C NMR and data HPLC-HRMS-ESI spectra of (*Z*)-8-Chloro-1-(2-(4-(4-fluorophenyl)thiazol-2-yl)hydrazineylidene)-4,4,6-trimethyl-5,6-dihydro-4*H*-pyrrolo[3,2,1-*ij*]quinolin-2(1*H*)-one 5d

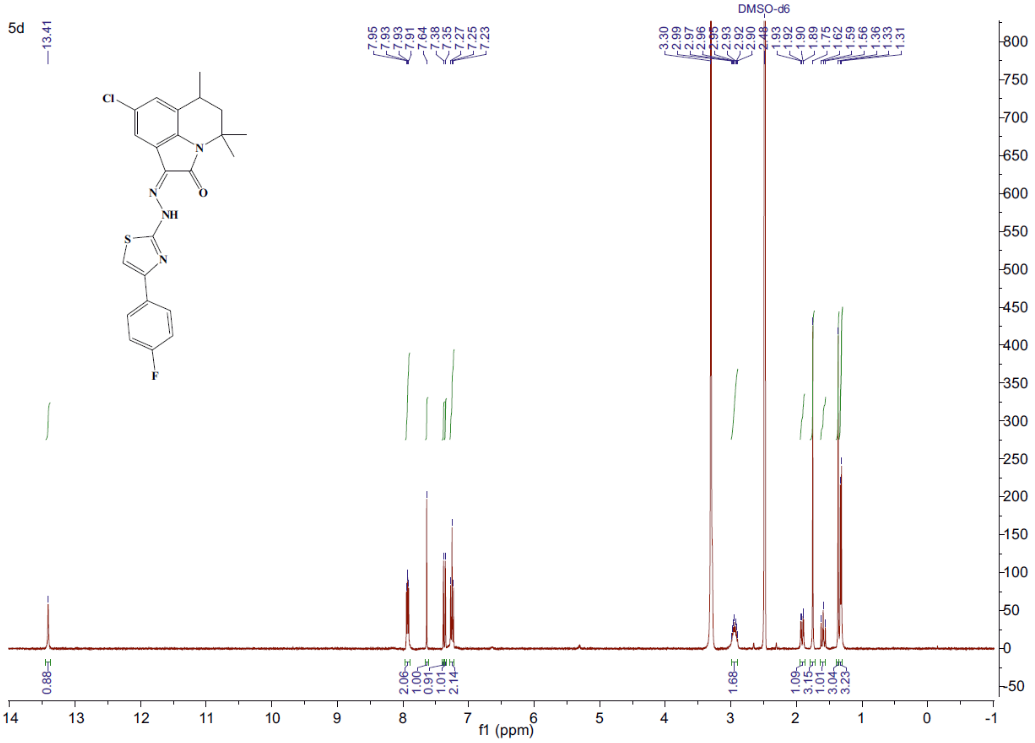

Figure S34. ^{1}H NMR (DMSO-d$_6$, 400 MHz) spectrum of compound 5d

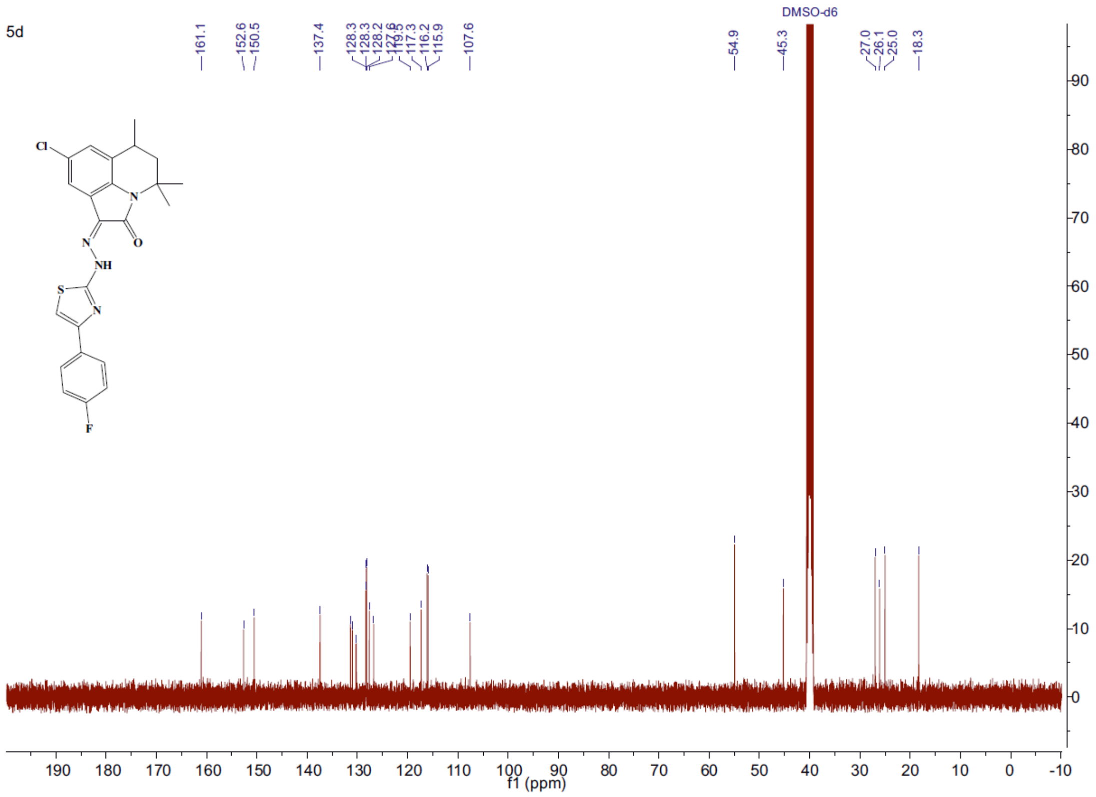

Figure S35. ^{13}C NMR (DMSO-d_6, 101 MHz) spectrum of compound 5d

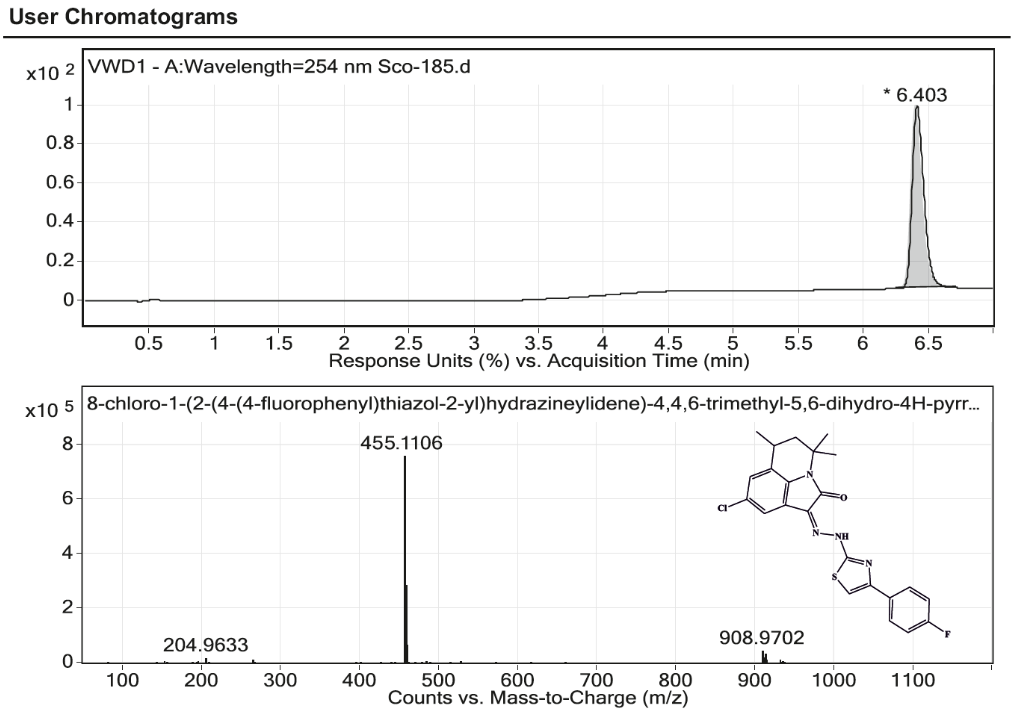

Figure S36. Data of HPLC-MS-ESI analysis of 5d

^{1}H, ^{13}C NMR and data HPLC-HRMS-ESI spectra of (*Z*)-8-chloro-1-(2-(4-(4-methoxyphenyl)thiazol-2-yl)hydrazineylidene)-4,4,6-trimethyl-5,6-dihydro-4*H*-pyrrolo[3,2,1-*ij*]quinolin-2(1*H*)-one 5e

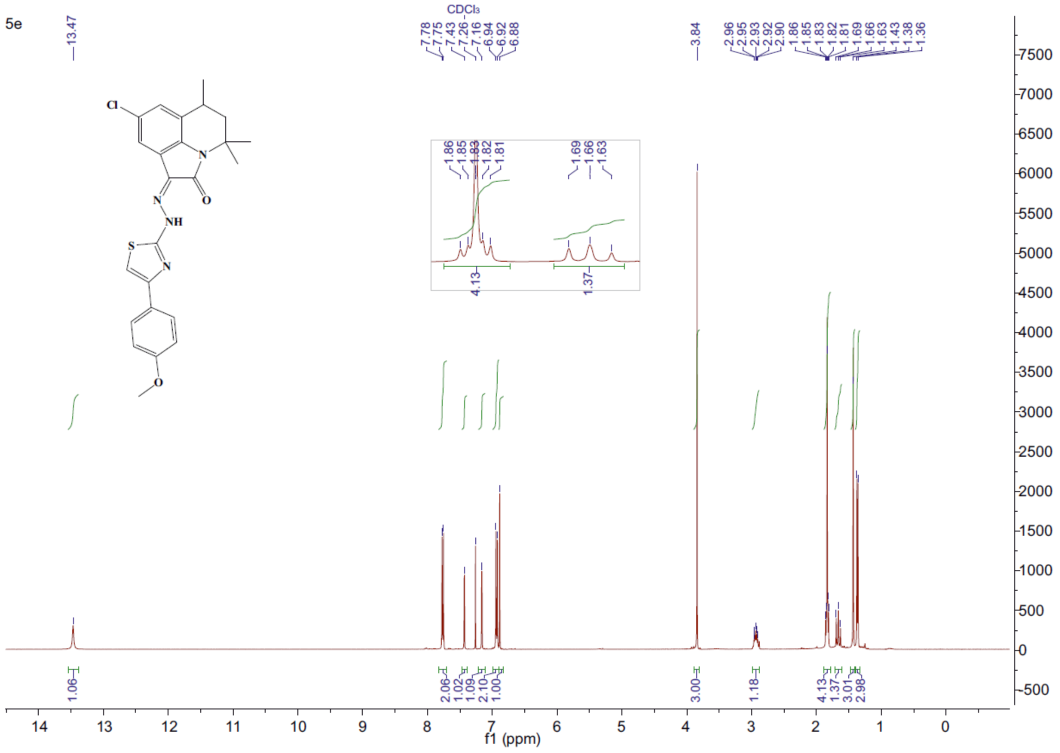

Figure S37. ^{1}H NMR (CDCl₃, 400 MHz) spectrum of compound 5e

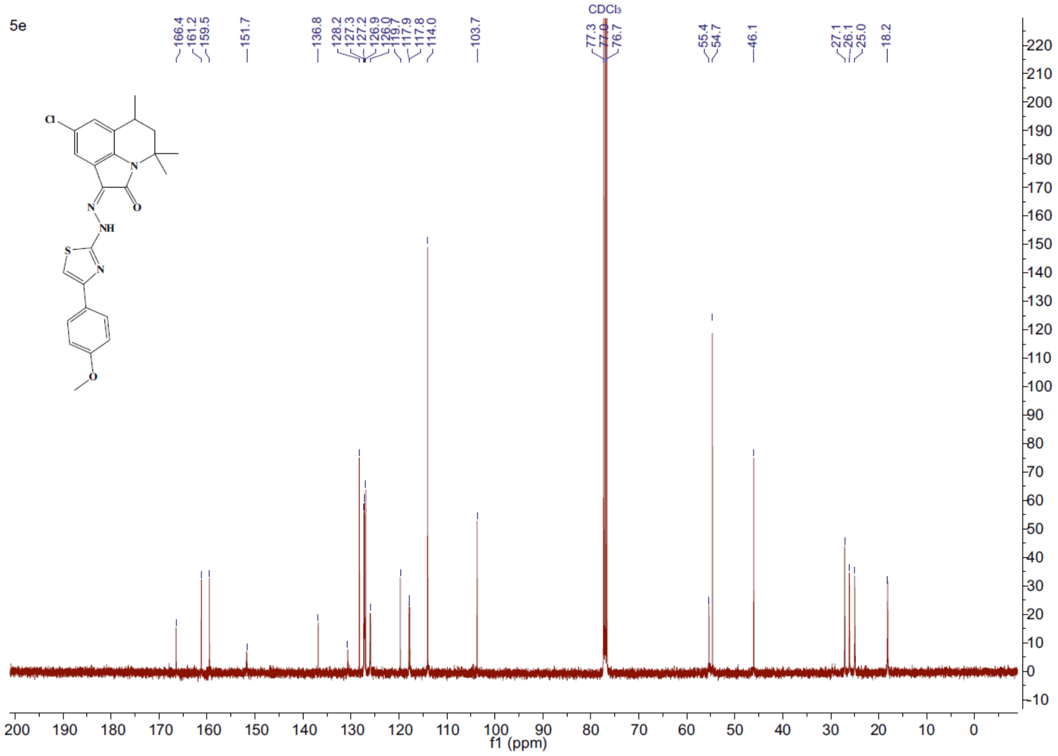

Figure S38. ^{13}C NMR (CDCl₃, 101 MHz) spectrum of compound 5e

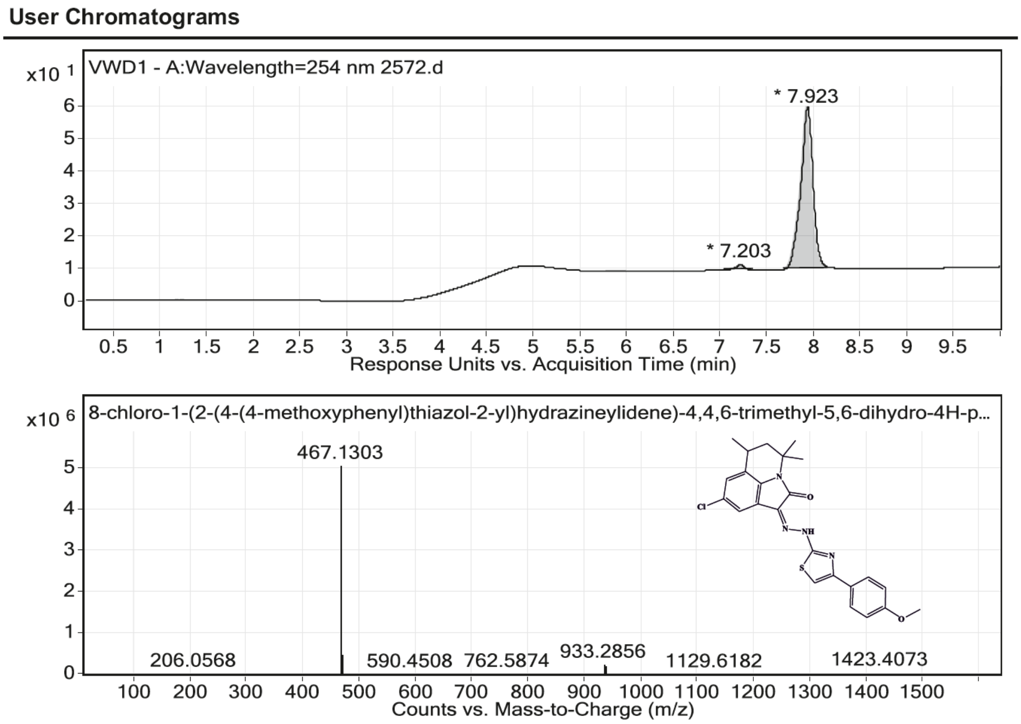

Figure S39. Data of HPLC-MS-ESI analysis of 5e

^{1}H, ^{13}C NMR and data HPLC-HRMS-ESI spectra of (*Z*)-8-fluoro-1-(2-(4-(3-methoxyphenyl)thiazol-2-yl)hydrazineylidene)-4,4,6-trimethyl-5,6-dihydro-4*H*-pyrrolo[3,2,1-*ij*]quinolin-2(1*H*)-one 5f

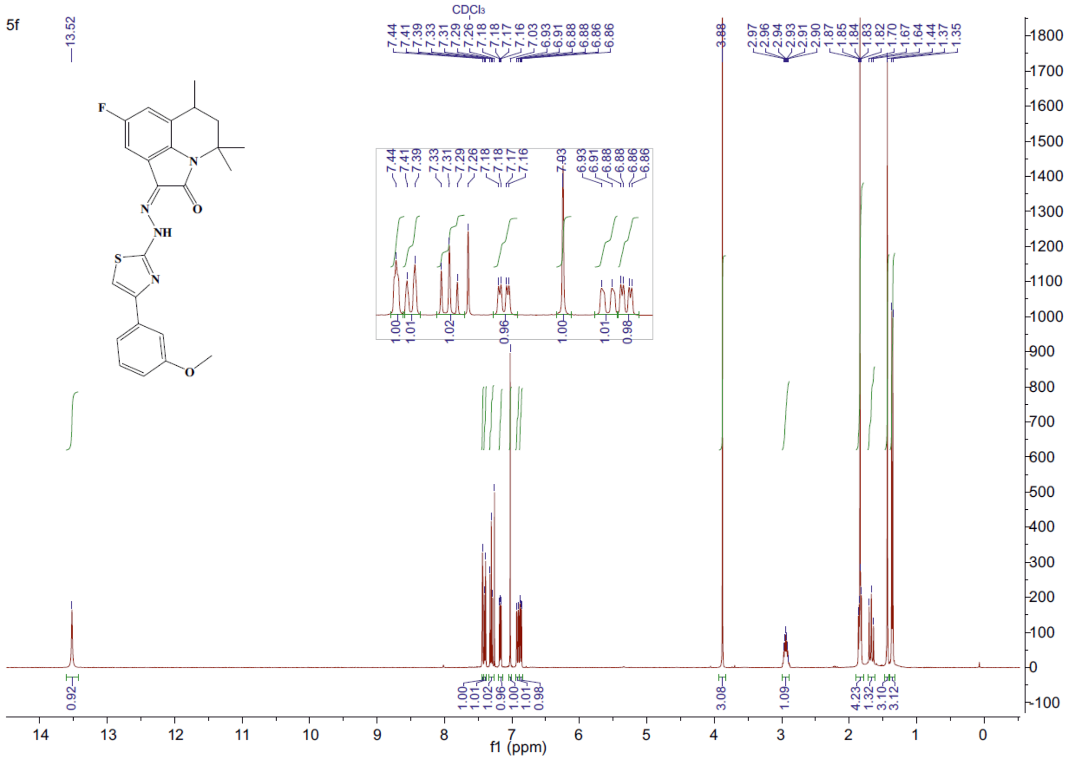

Figure S40. ^{1}H NMR ($CDCl_3$, 400 MHz) spectrum of compound 5f

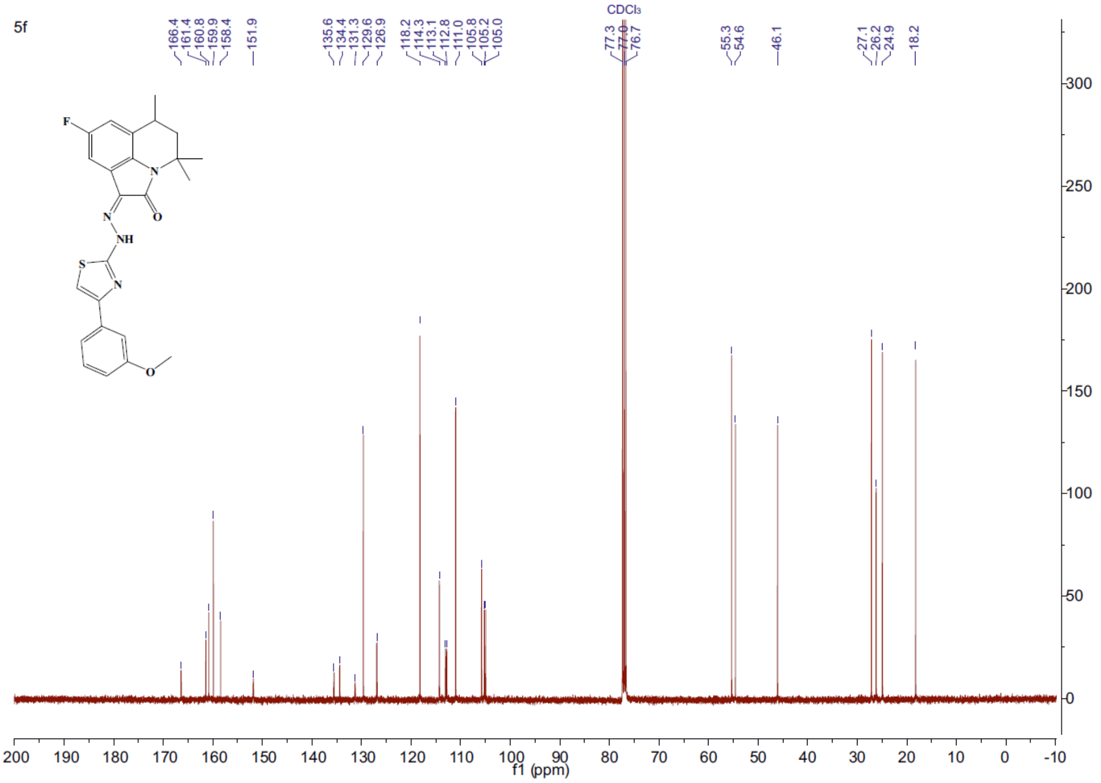

Figure S41. ^{13}C **NMR (** $CDCl_3$ **, 101 MHz) spectrum of compound 5f**

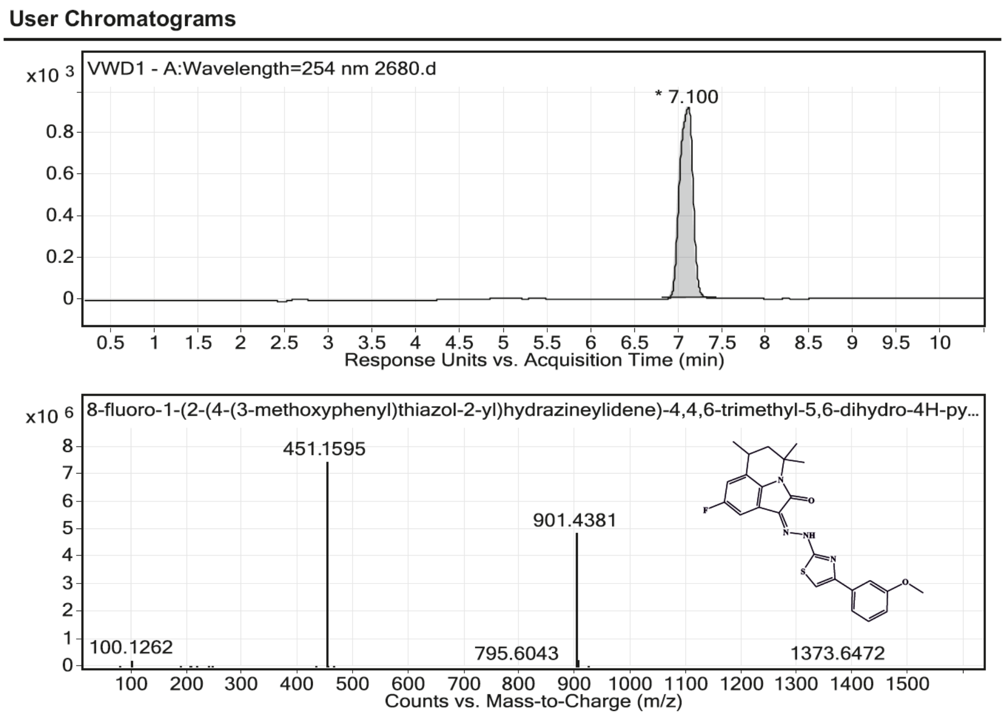

Figure S42. Data of HPLC-MS-ESI analysis of 5f

^{1}H, ^{13}C NMR and data HPLC-HRMS-ESI spectra of (*Z*)-1-(2-(4-(4-chlorophenyl)thiazol-2-yl)hydrazineylidene)-8-fluoro-4,4,6-trimethyl-6-phenyl-5,6-dihydro-4*H*-pyrrolo[3,2,1-*ij*]quinolin-2(1*H*)-one 5g

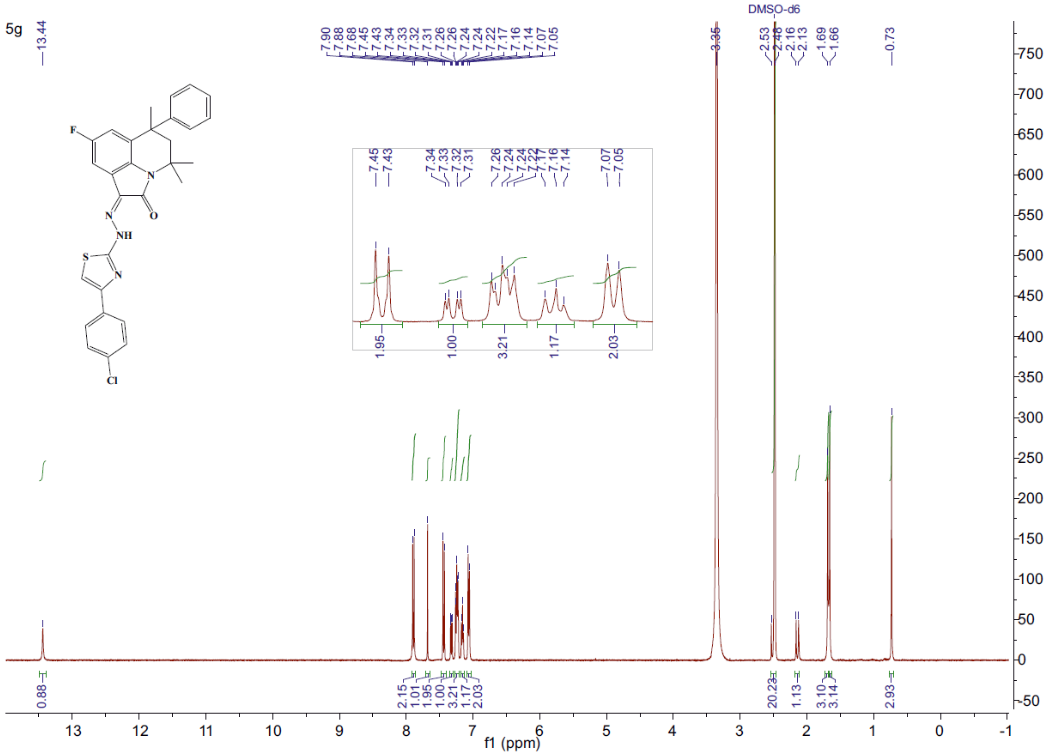

Figure S43. ^{1}H NMR (DMSO-d$_6$, 400 MHz) spectrum of compound 5g

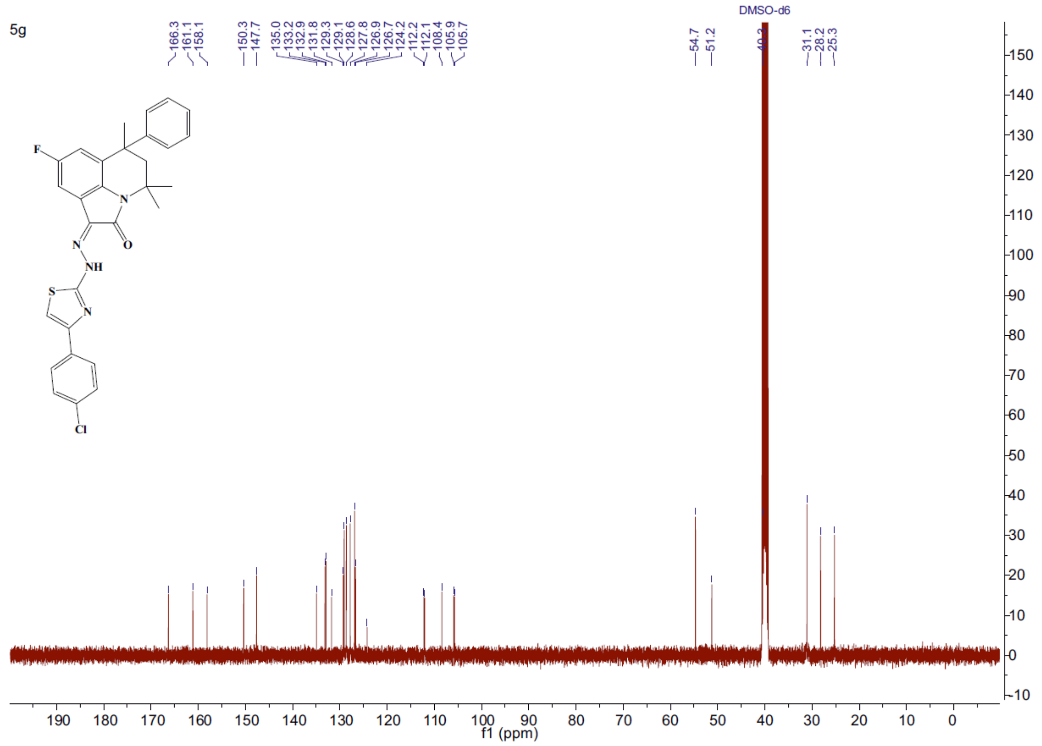

Figure S44. ^{13}C NMR (DMSO-d$_6$, 101 MHz) spectrum of compound 5g

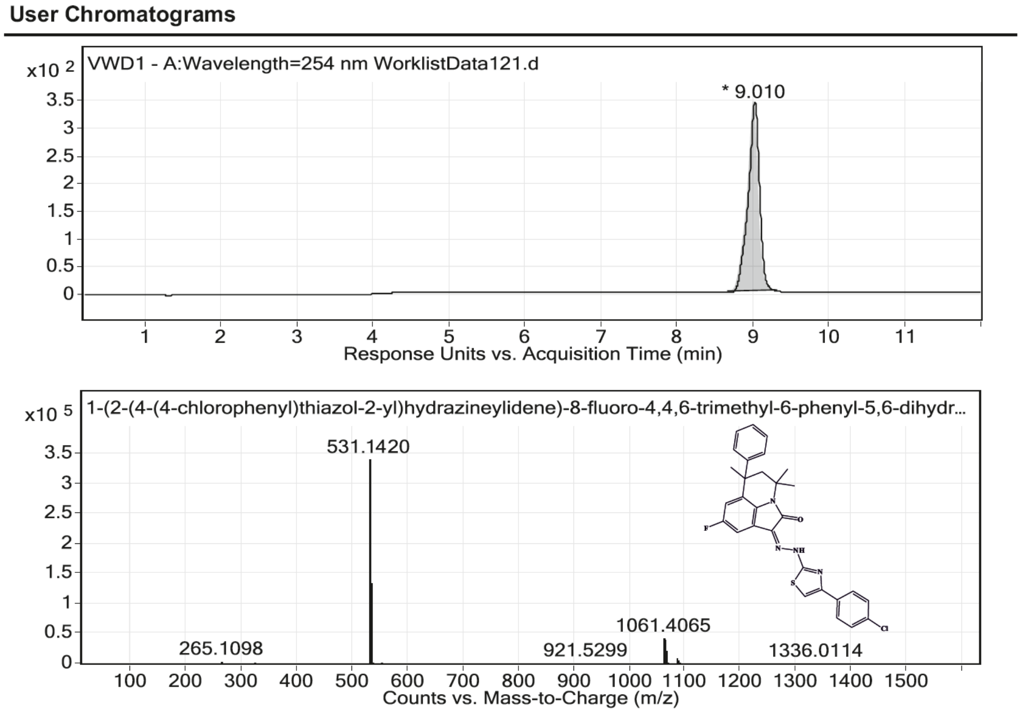

Figure S45. Data of HPLC-MS-ESI analysis of 5g

^{1}H, ^{13}C NMR and data HPLC-HRMS-ESI spectra of (*Z*)-8-bromo-1-(2-(4-(4-chlorophenyl)thiazol-2-yl)hydrazineylidene)-4,4,6-trimethyl-6-phenyl-5,6-dihydro-4*H*-pyrrolo[3,2,1-*ij*]quinolin-2(1*H*)-one 5h

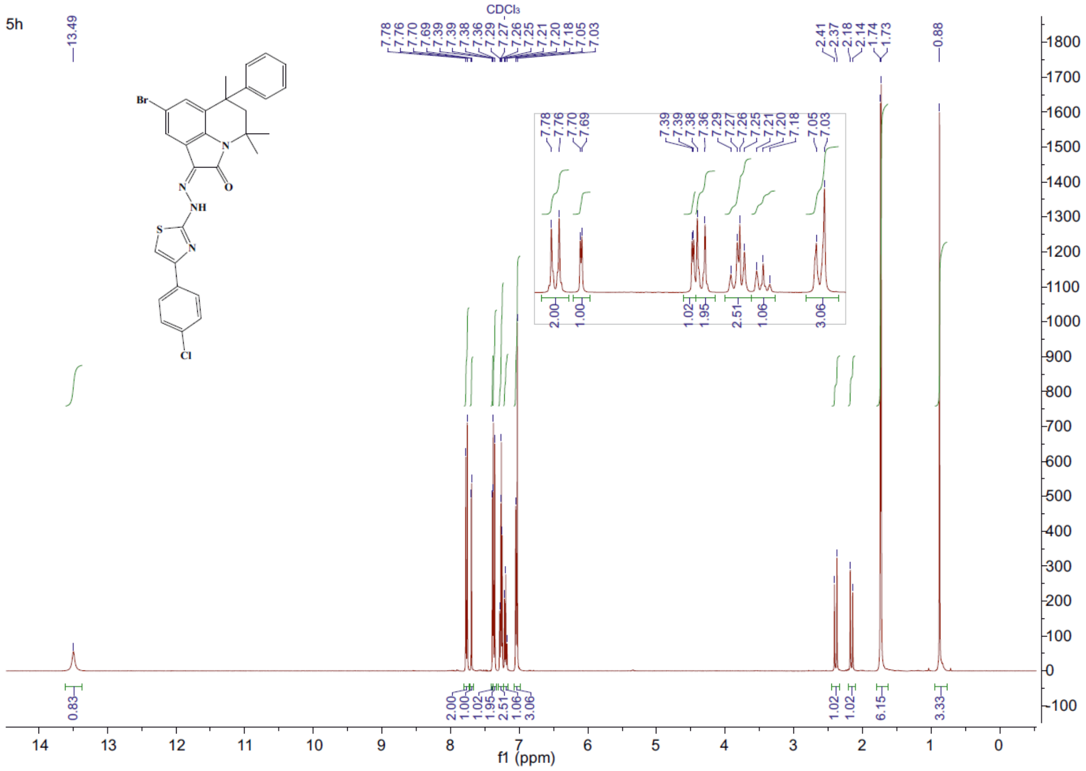

Figure S46. ^{1}H NMR ($CDCl_3$, 400 MHz) spectrum of compound 5h

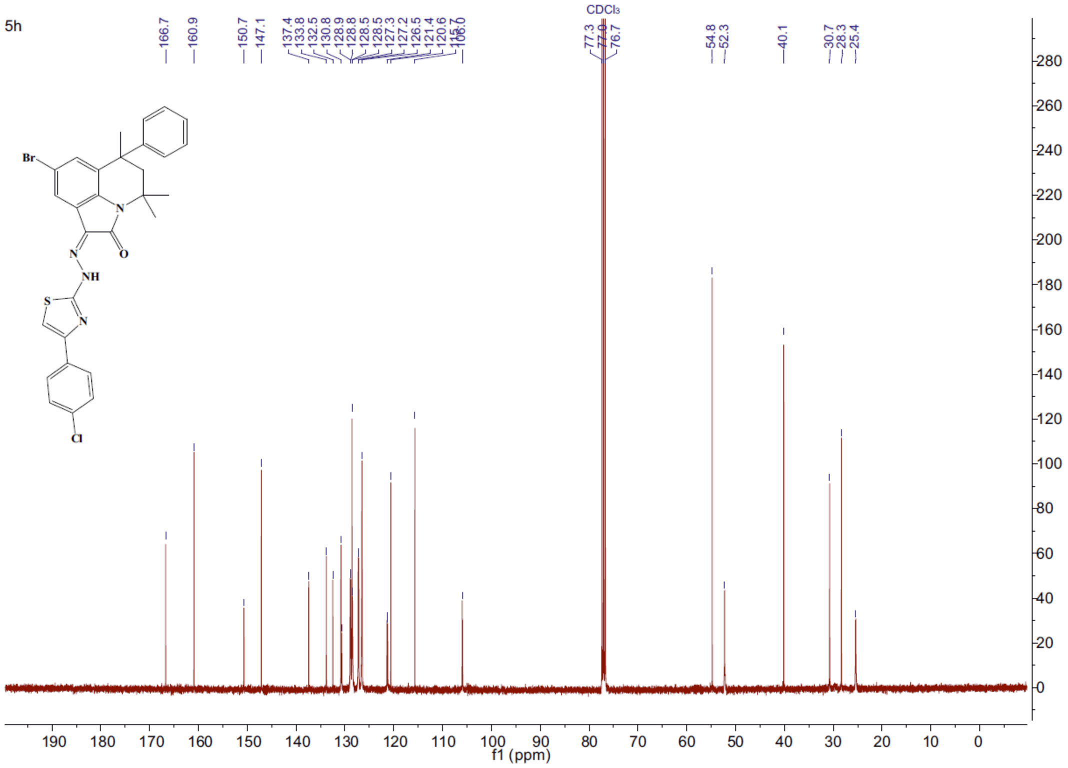

Figure S47. ^{13}C **NMR ($CDCl_3$, 101 MHz) spectrum of compound 5h**

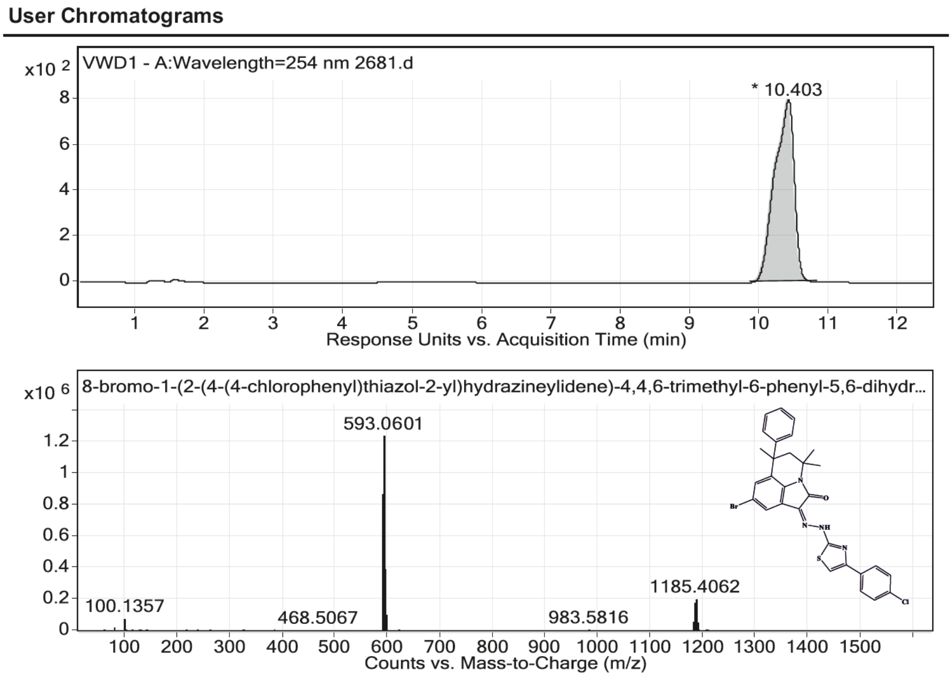

Figure S48. Data of HPLC-MS-ESI analysis of 5h

^{1}H, ^{13}C NMR and data HPLC-HRMS-ESI spectra of (*Z*)-6-(4-chlorophenyl)-1-(2-(4-(4-methoxyphenyl)thiazol-2-yl)hydrazineylidene)-4,4,6-trimethyl-5,6-dihydro-4*H*-pyrrolo[3,2,1-*ij*]quinolin-2(1*H*)-one 5i

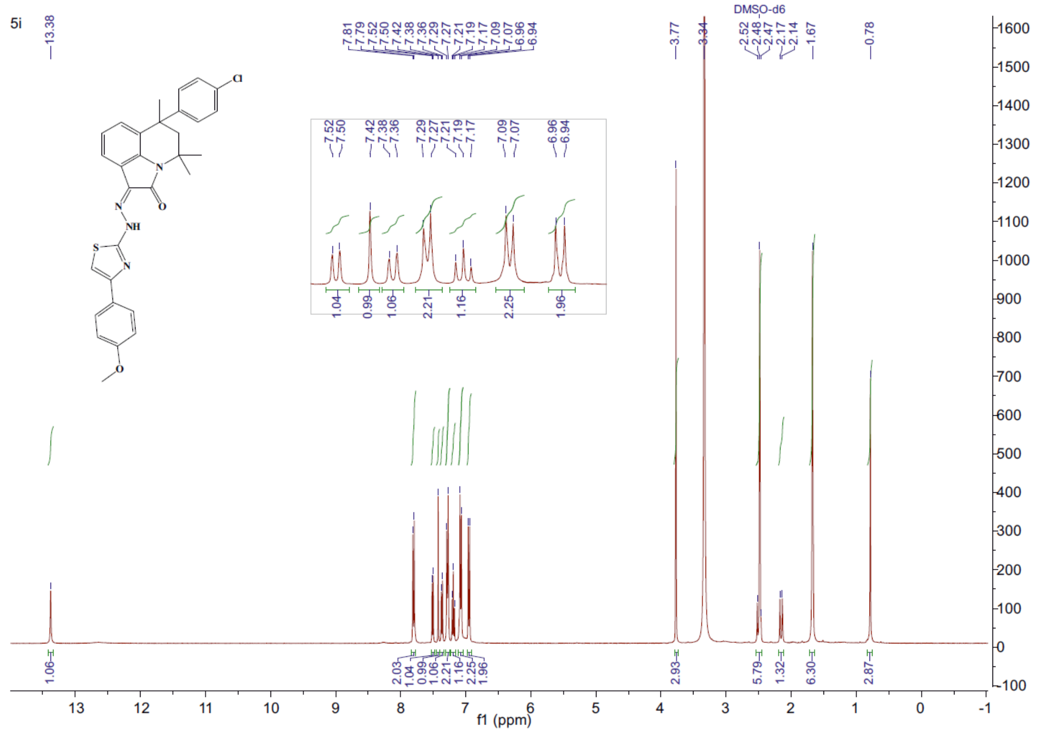

Figure S49. ^{1}H NMR (DMSO-d₆, 400 MHz) spectrum of compound 5i

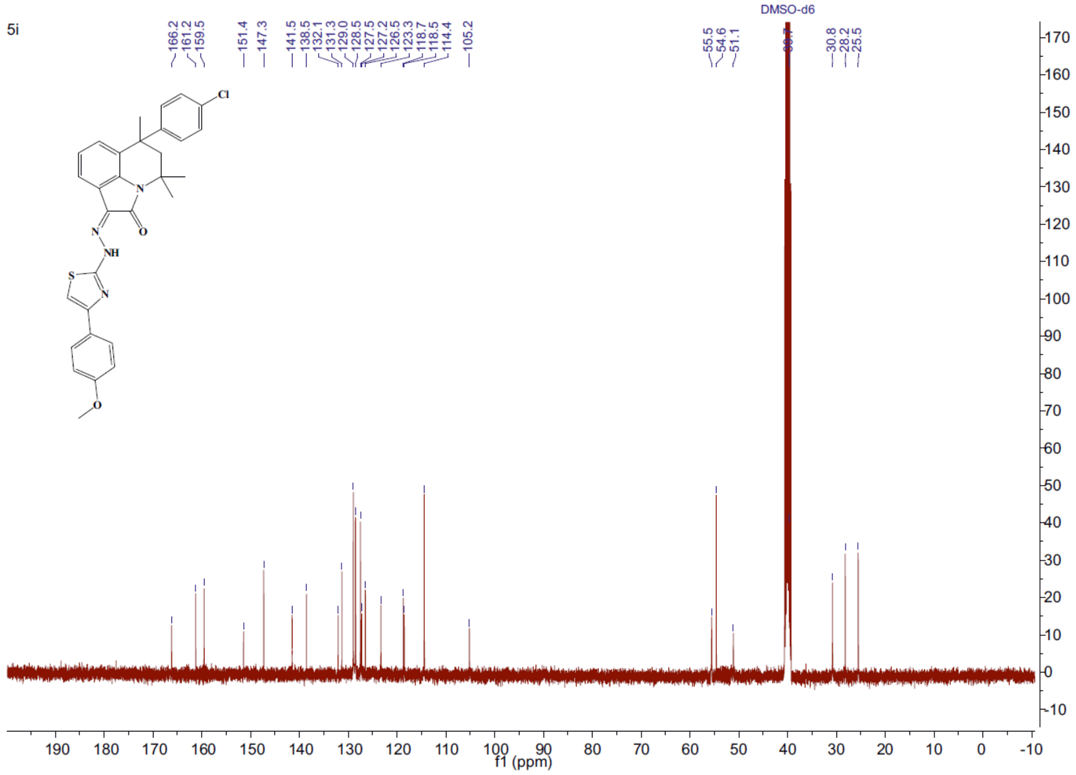

Figure S50. ^{13}C NMR (DMSO-d₆, 101 MHz) spectrum of compound 5i

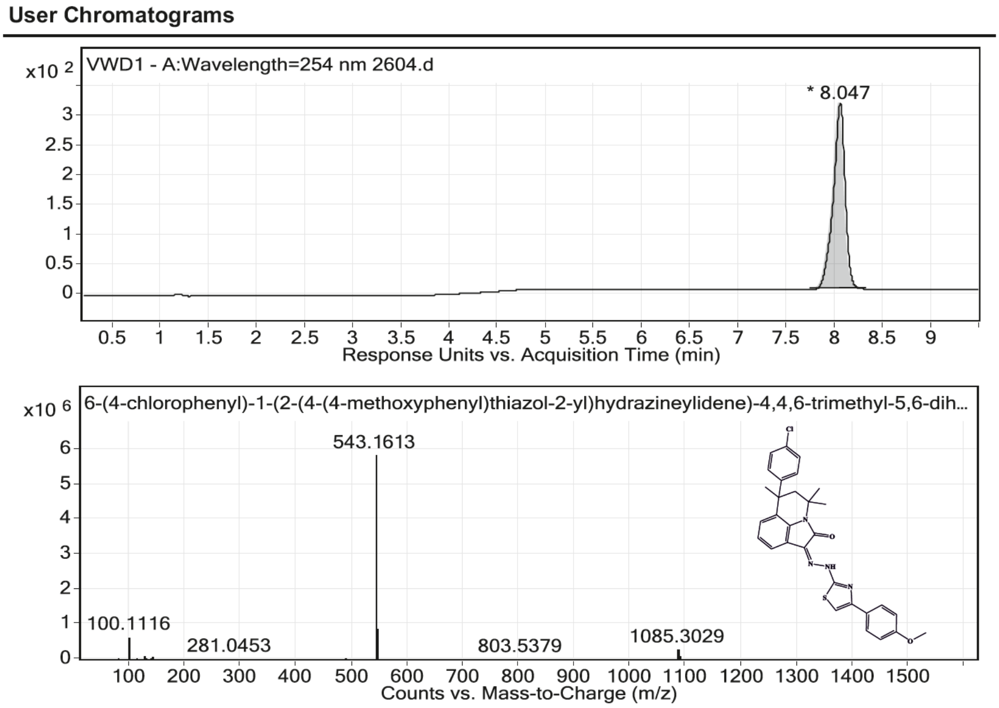

Figure S51. Data of HPLC-MS-ESI analysis of 5i

^1H, ^{13}C NMR and data HPLC-HRMS-ESI spectra of (*Z*)-8-chloro-6-(4-chlorophenyl)-1-(2-(4-(4-methoxyphenyl)thiazol-2-yl)hydrazineylidene)-4,4,6-trimethyl-5,6-dihydro-4*H*-pyrrolo[3,2,1-*ij*]quinolin-2(1*H*)-one 5j

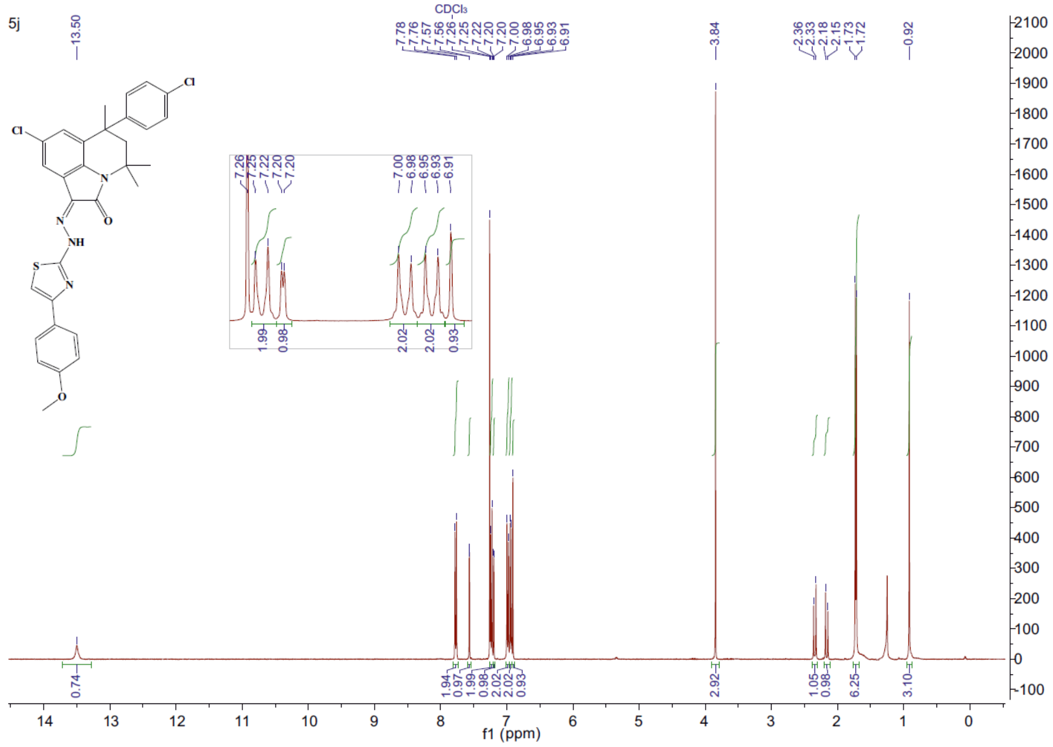

Figure S52. ^1H NMR ($CDCl_3$, 400 MHz) spectrum of compound 5j

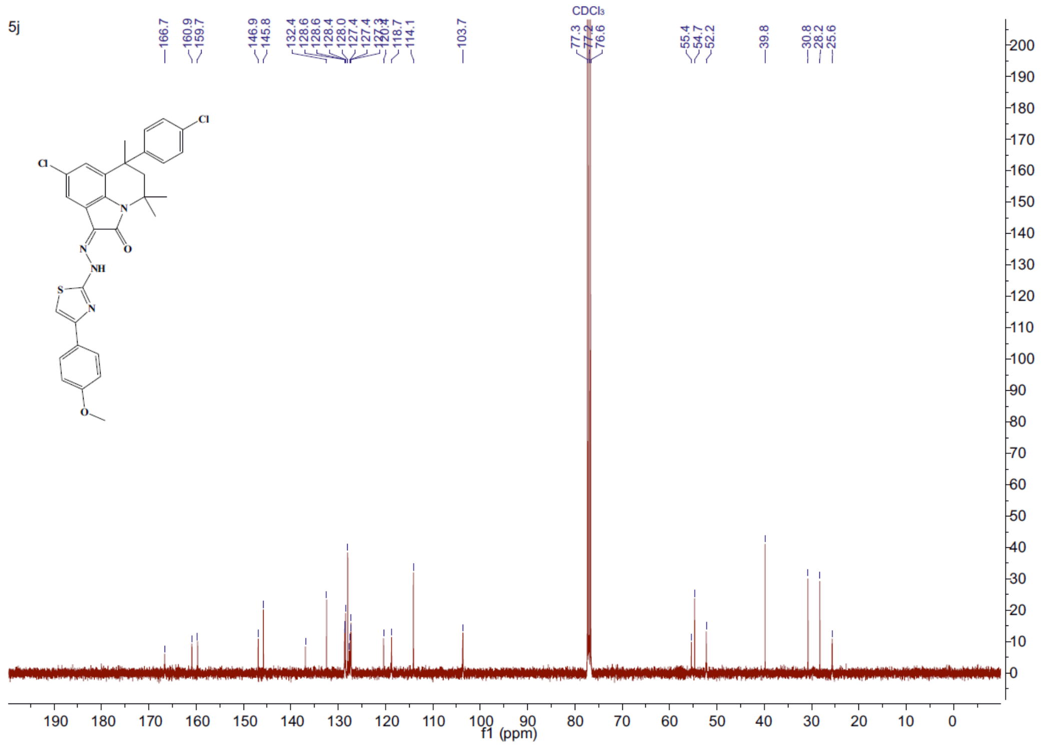

Figure S53. ^{13}C NMR ($CDCl_3$, 101 MHz) spectrum of compound 5j

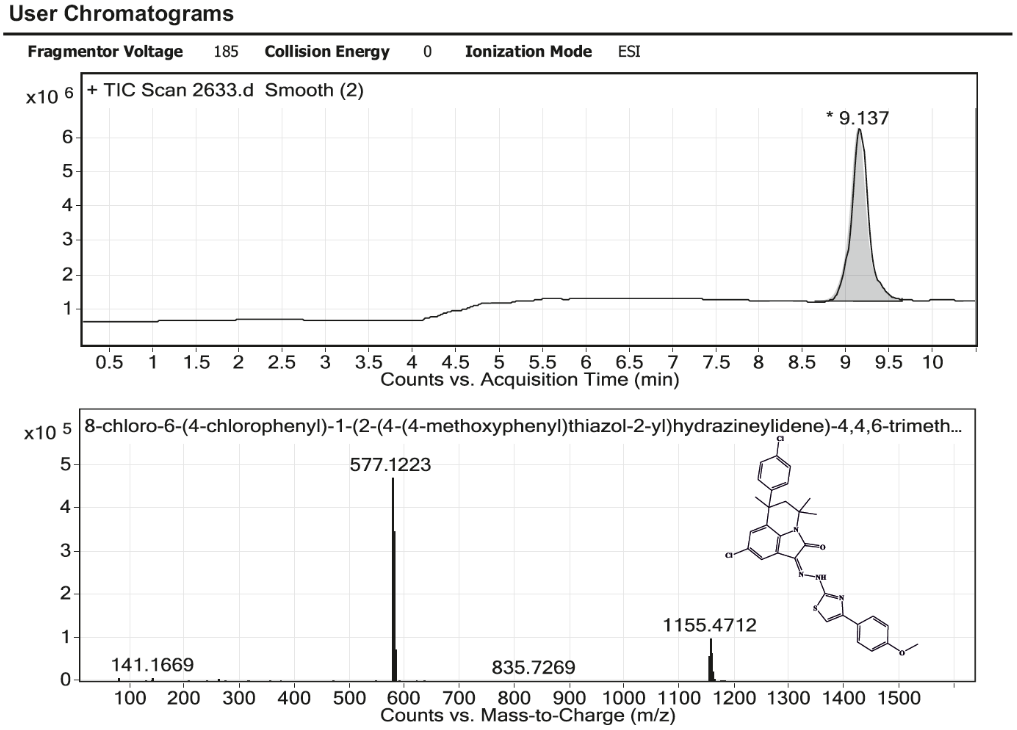

Figure S54. Data of HPLC-MS-ESI analysis of 5j

^{1}H, ^{13}C NMR and data HPLC-HRMS-ESI spectra of (*Z*)-6-(4-chlorophenyl)-1-(2-(4-(4-chlorophenyl)thiazol-2-yl)hydrazineylidene)-8-fluoro-4,4,6-trimethyl-5,6-dihydro-4*H*-pyrrolo[3,2,1-*ij*]quinolin-2(1*H*)-one 5k

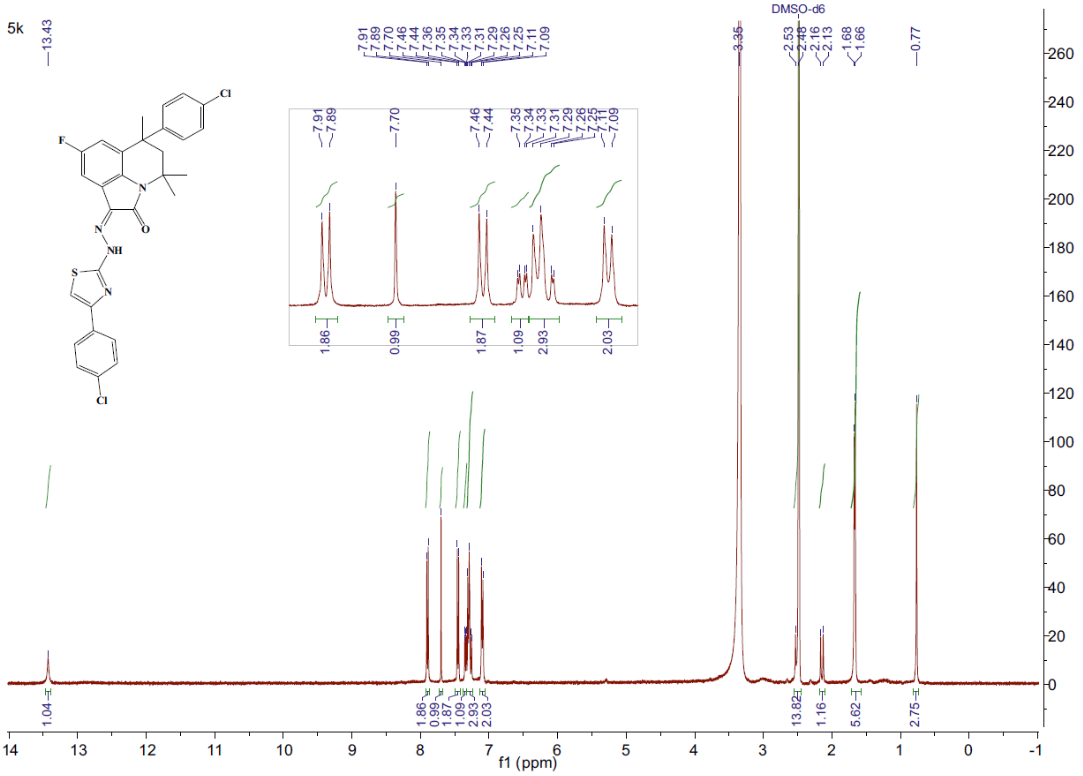

Figure S55. ^{1}H NMR (DMSO-d$_6$, 400 MHz) spectrum of compound 5k

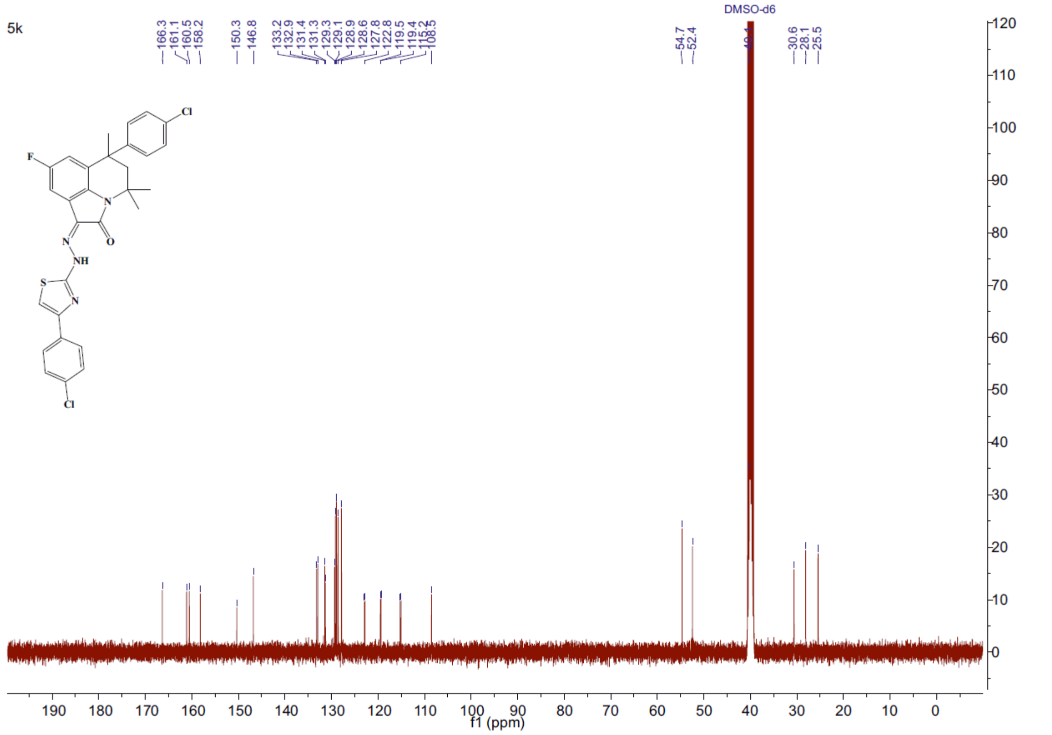

Figure S56. ^{13}C NMR (DMSO-d$_6$, 101 MHz) spectrum of compound 5k

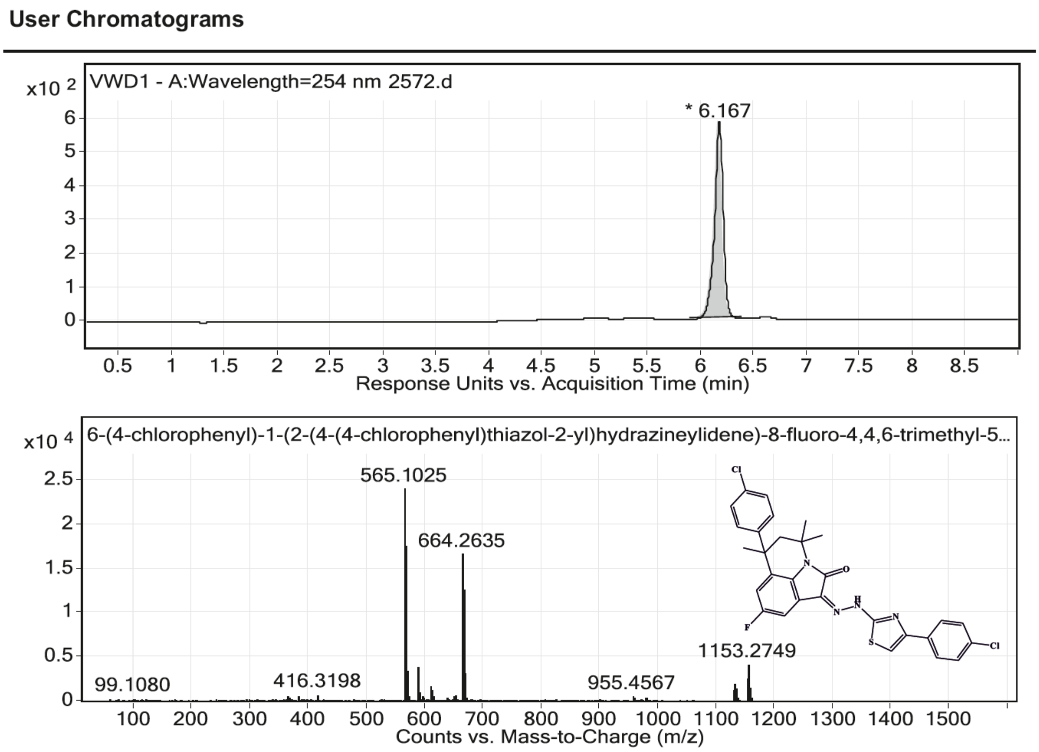

Figure S57. Data of HPLC-MS-ESI analysis of 5k

^{1}H, ^{13}C NMR and data HPLC-HRMS-ESI spectra of (*Z*)-6-(4-chlorophenyl)-8-fluoro-1-(2-(4-(4-fluorophenyl)thiazol-2-yl)hydrazineylidene)-4,4,6-trimethyl-5,6-dihydro-4*H*-pyrrolo[3,2,1-*ij*]quinolin-2(1*H*)-one 5l

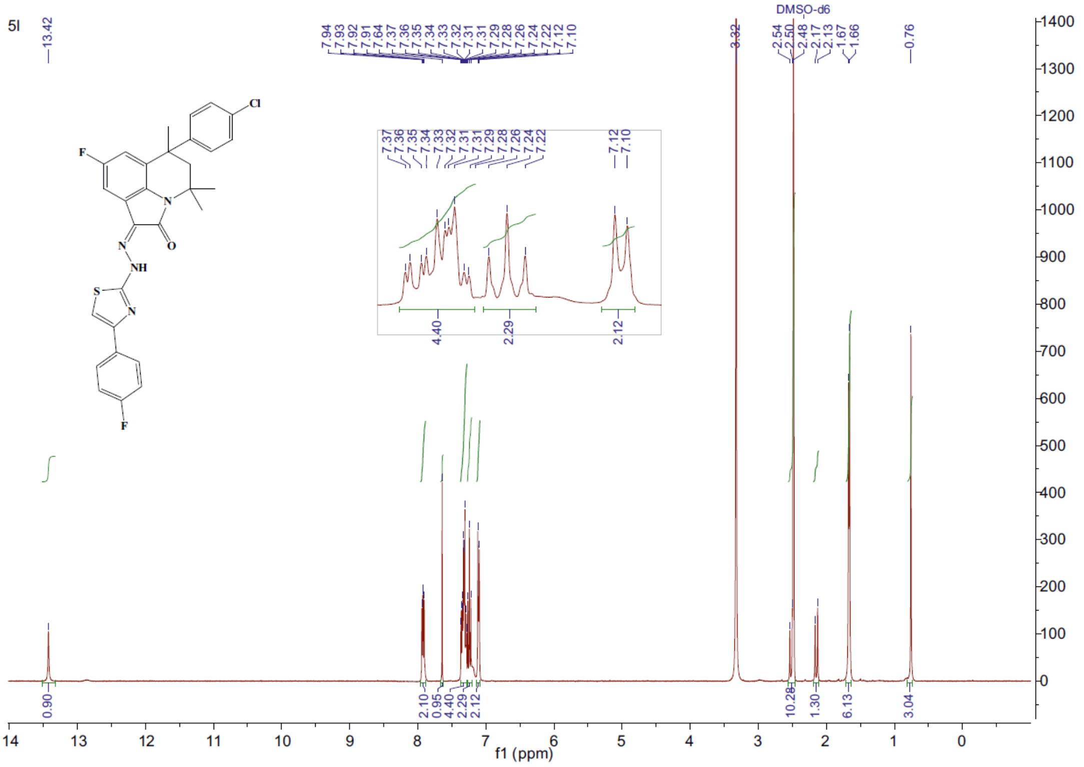

Figure S58. ^{1}H NMR (DMSO-d$_6$, 400 MHz) spectrum of compound 5l

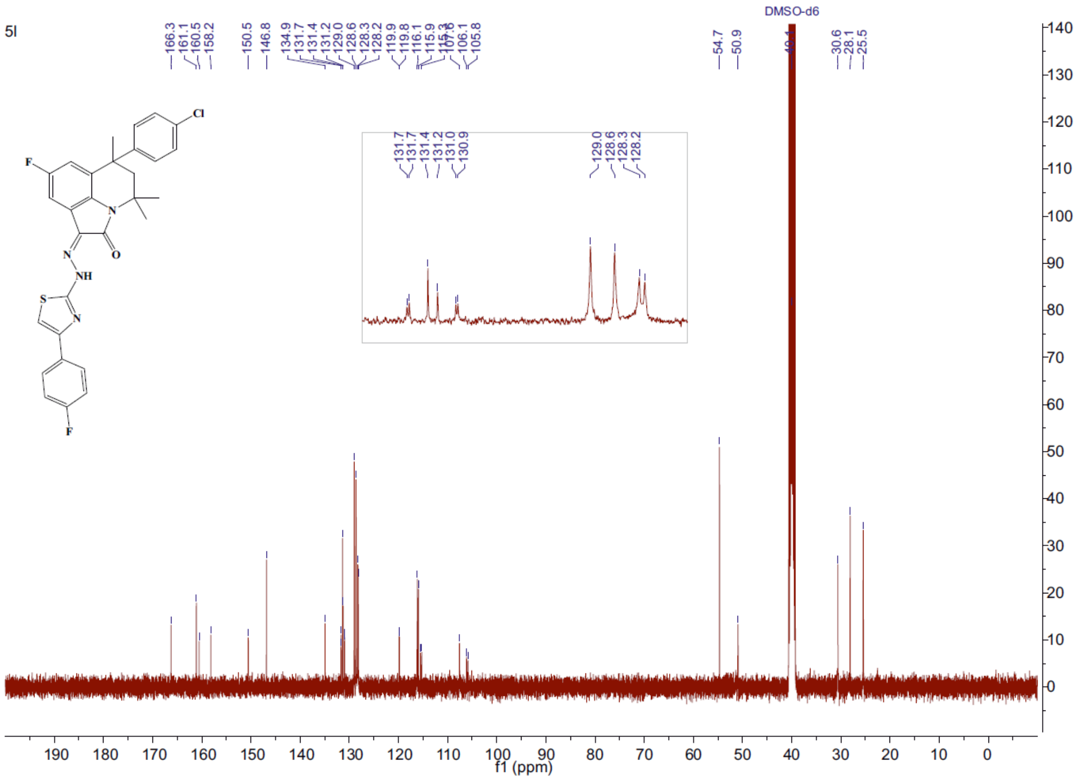

Figure S59. ^{13}C NMR (DMSO-d$_6$, 101 MHz) spectrum of compound 5l

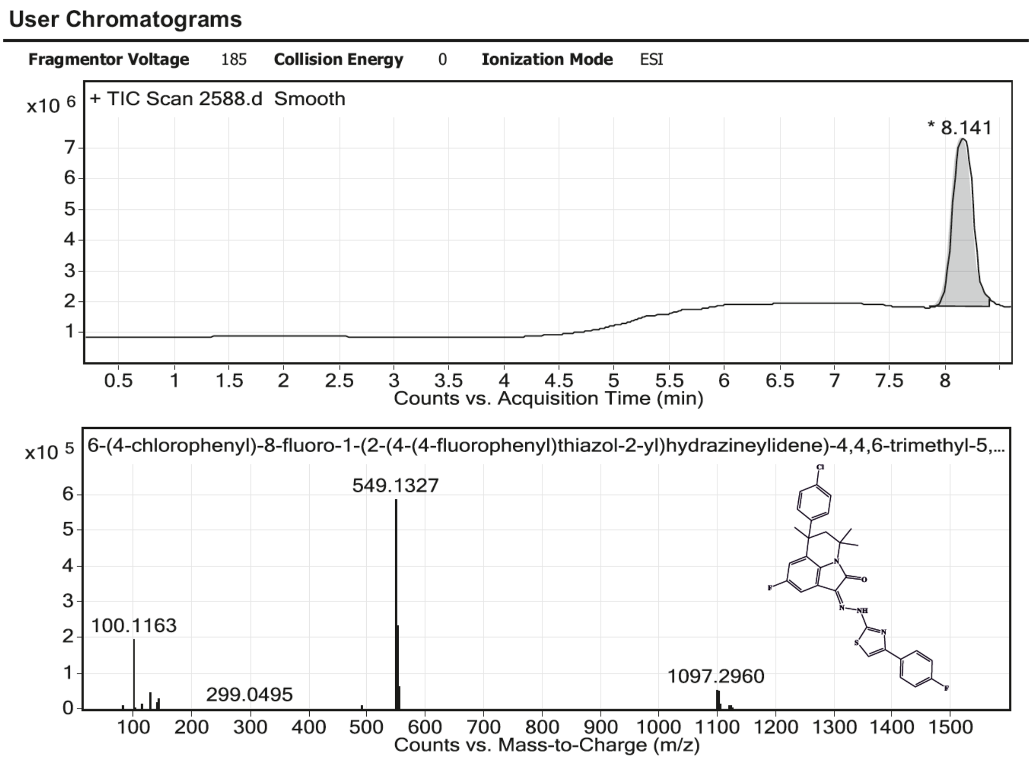

Figure S60. Data of HPLC-MS-ESI analysis of 5l

^{1}H, ^{13}C NMR and data HPLC-HRMS-ESI spectra of (*Z*)-6-(4-chlorophenyl)-1-(2-(4-(3-chlorophenyl)thiazol-2-yl)hydrazineylidene)-8-fluoro-4,4,6-trimethyl-5,6-dihydro-4*H*-pyrrolo[3,2,1-*ij*]quinolin-2(1*H*)-one 5m

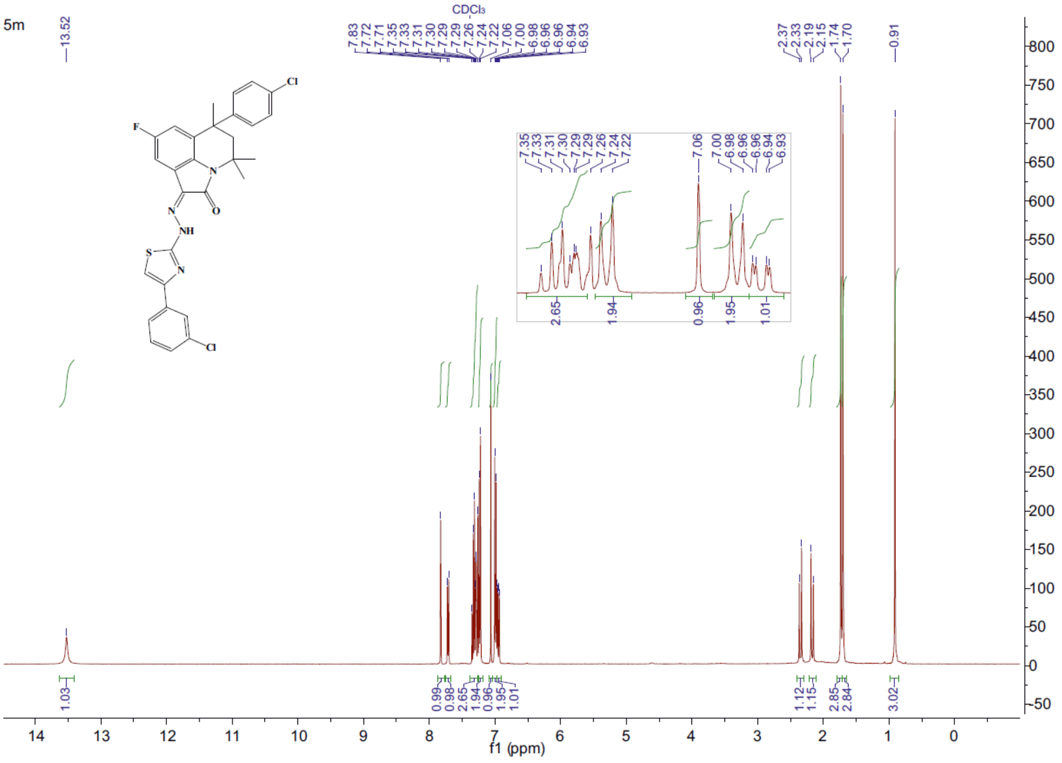

Figure S61. **^{1}H NMR ($CDCl_3$, 400 MHz) spectrum of compound 5m**

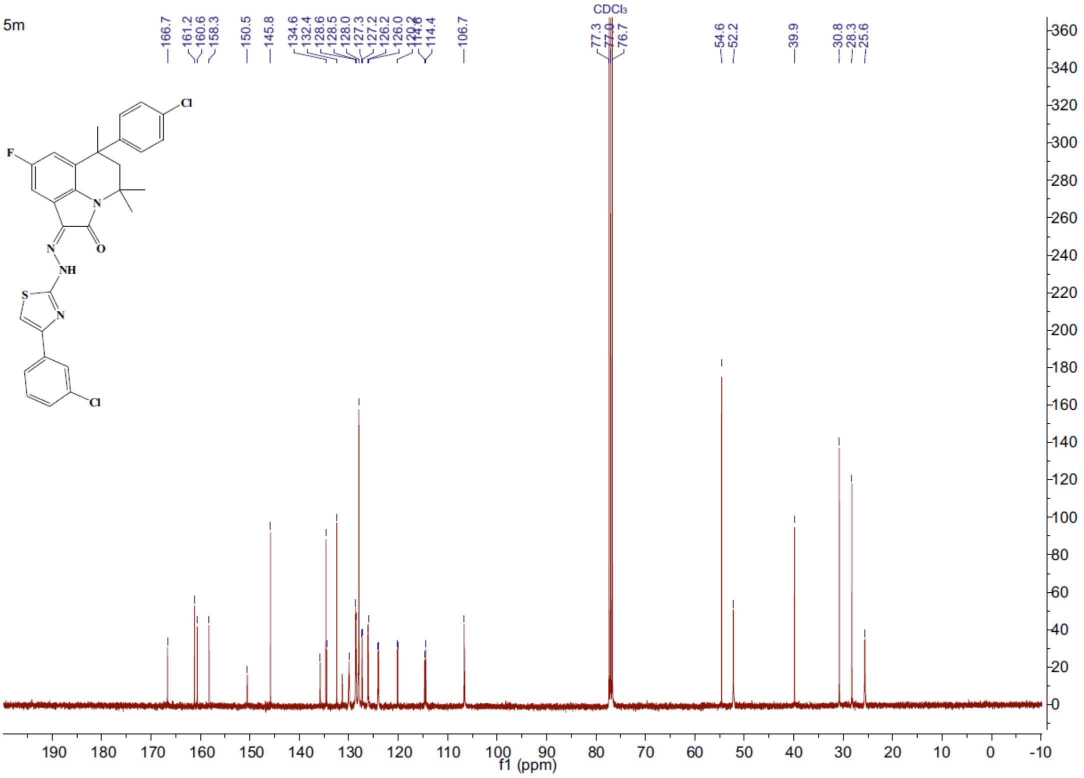

Figure S62. **^{13}C NMR ($CDCl_3$, 101 MHz) spectrum of compound 5m**

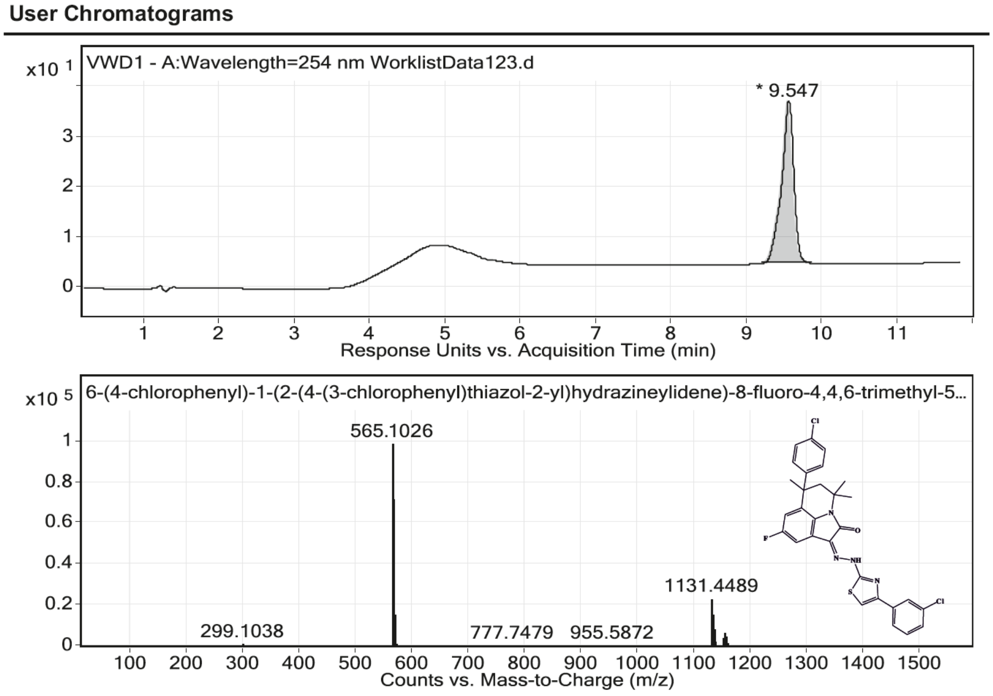

Figure S63. Data of HPLC-MS-ESI analysis of 5m

^{1}H, ^{13}C NMR and data HPLC-HRMS-ESI spectra of (*Z*)-6-(4-chlorophenyl)-8-fluoro-1-(2-(4-(3-methoxyphenyl)thiazol-2-yl)hydrazineylidene)-4,4,6-trimethyl-5,6-dihydro-4*H*-pyrrolo[3,2,1-*ij*]quinolin-2(1*H*)-one 5n

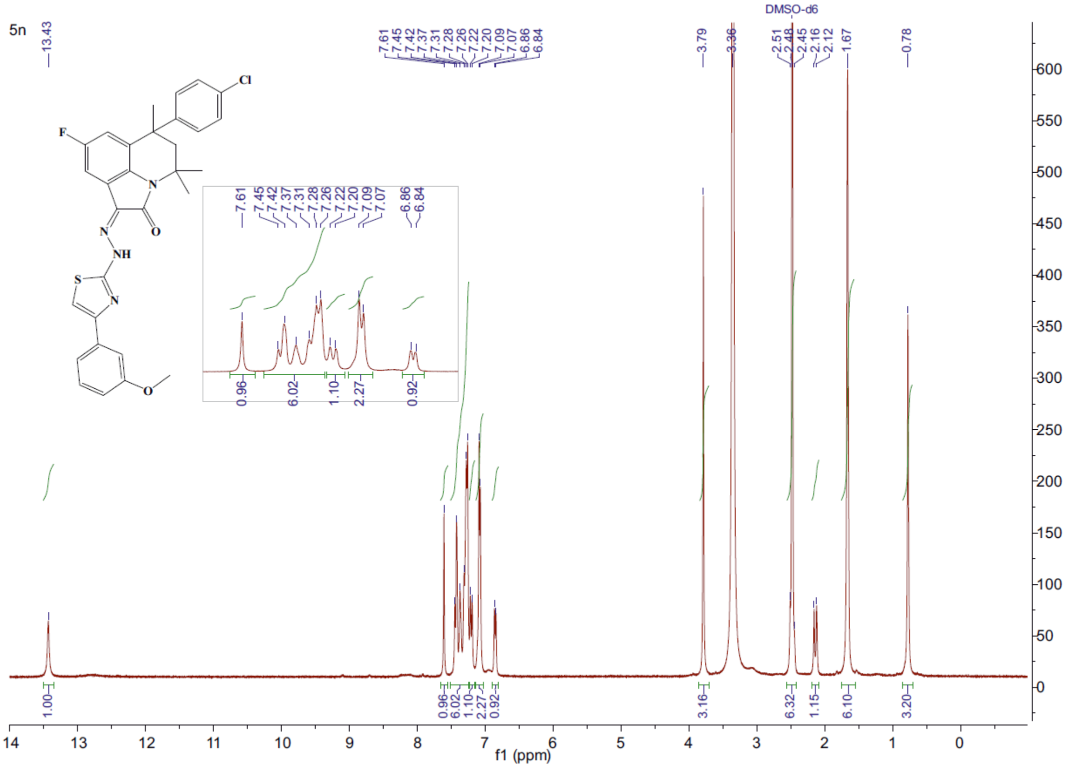

Figure S64. ^{1}H NMR (DMSO-d$_6$, 400 MHz) spectrum of compound 5n

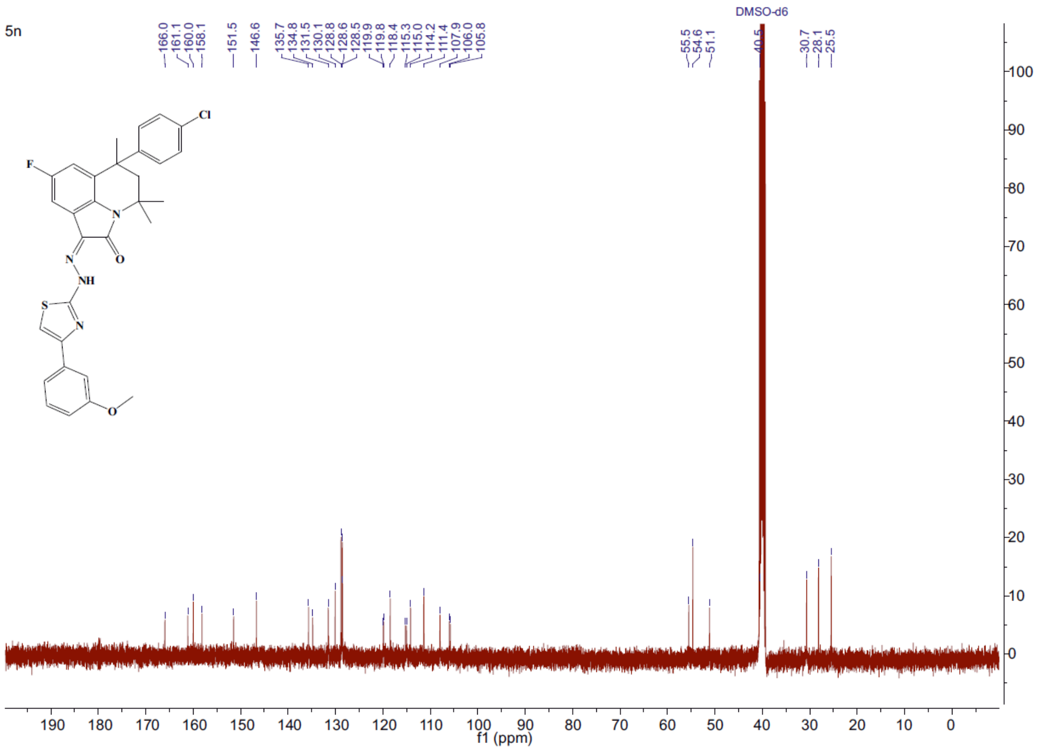

Figure S65. ^{13}**C NMR (DMSO-d**$_6$**, 101 MHz) spectrum of compound 5n**

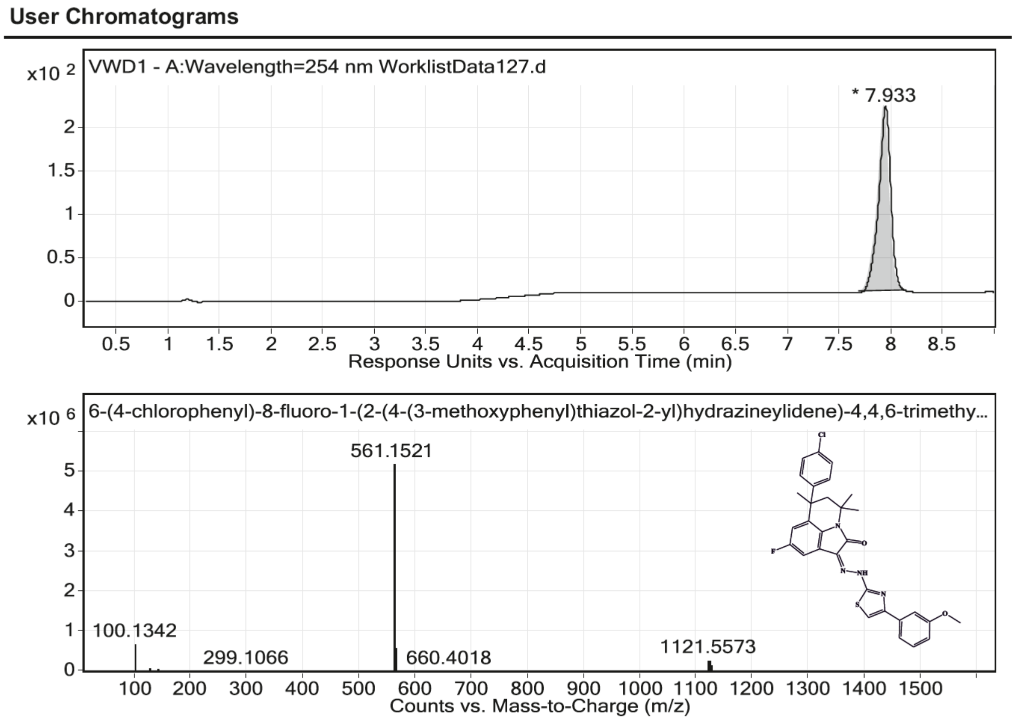

Figure S66. Data of HPLC-MS-ESI analysis of 5n

^{1}H, ^{13}C NMR and data HPLC-HRMS-ESI spectra of (*Z*)-8-bromo-6-(4-chlorophenyl)-4,4,6-trimethyl-1-(2-(4-phenylthiazol-2-yl)hydrazineylidene)-5,6-dihydro-4*H*-pyrrolo[3,2,1-*ij*]quinolin-2(1*H*)-one 5o

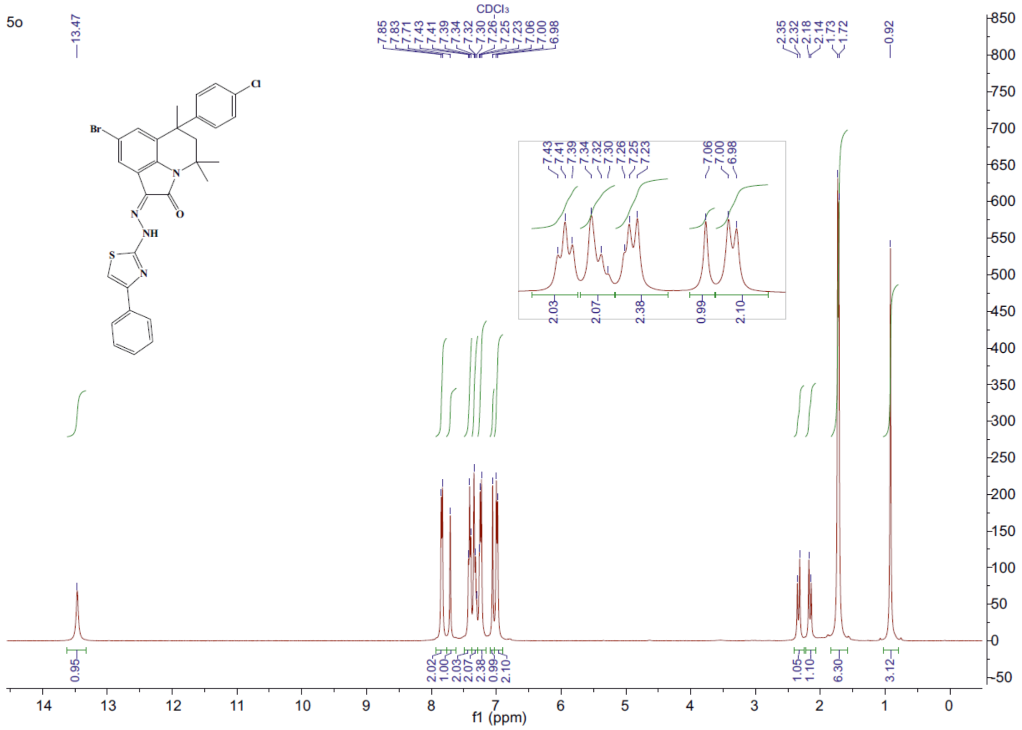

Figure S67. ^{1}H NMR ($CDCl_3$, 400 MHz) spectrum of compound 5o

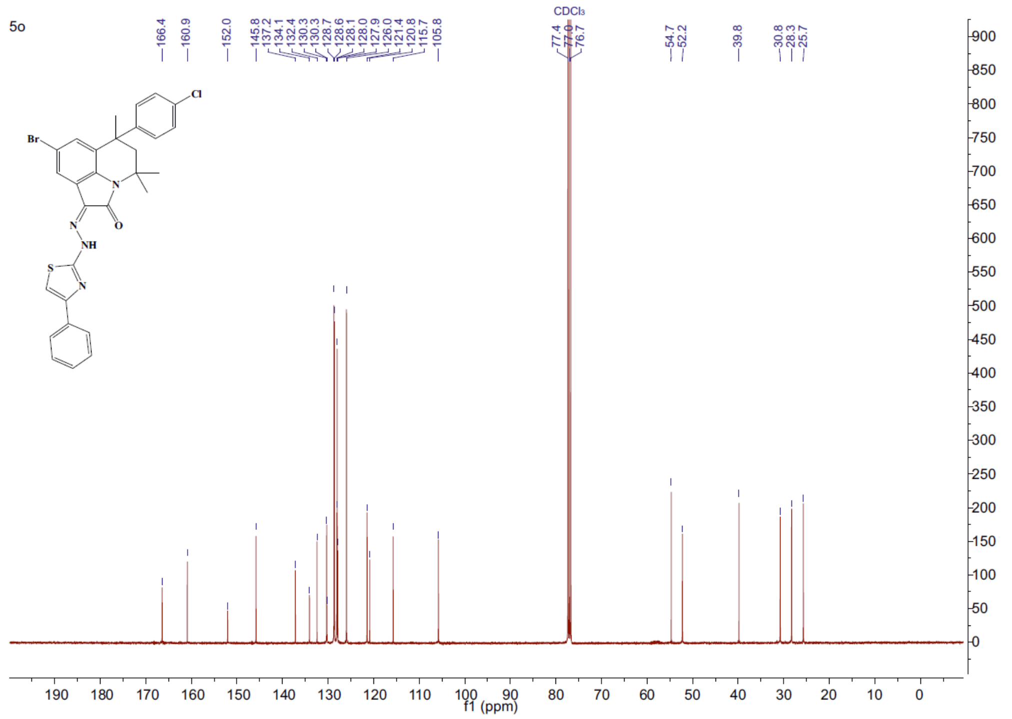

Figure S68. ^{13}C NMR ($CDCl_3$, 101 MHz) spectrum of compound 5o

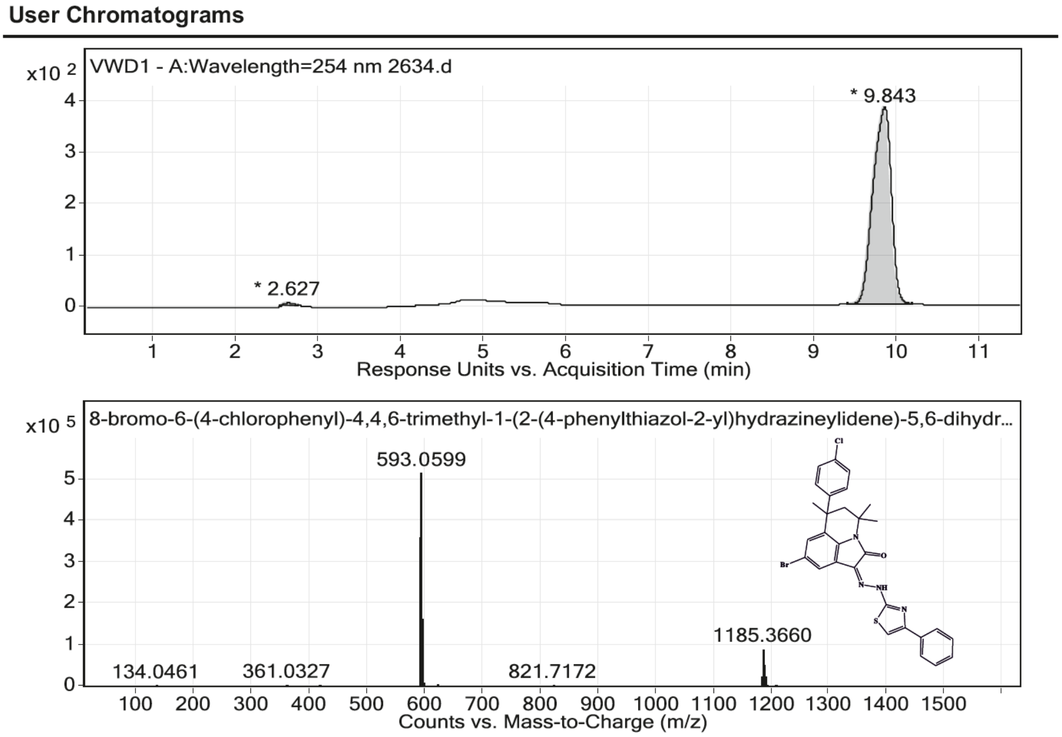

Figure S69. Data of HPLC-MS-ESI analysis of 5o

The dependence of inhibition of factor Xa- and XIa-induced chromogenic substrate hydrolysis on the compound concentration.

The dependence of inhibition of factor Xa- and XIa-induced chromogenic substrate hydrolysis on the concentration of 5d

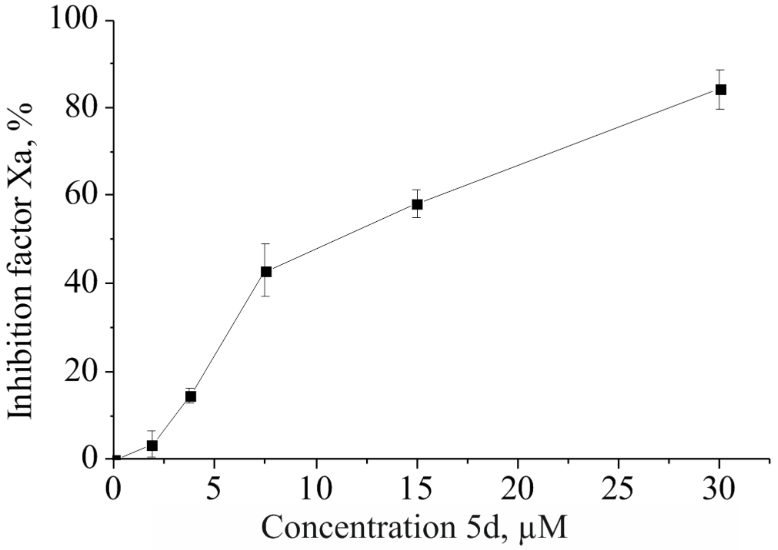

Figure S70. The dependence of inhibition of factor Xa-induced chromogenic substrate hydrolysis on the concentration of 5d

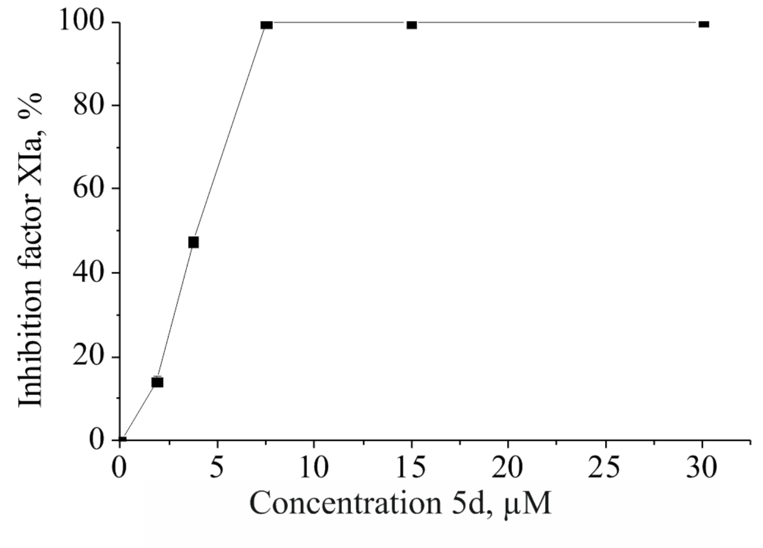

Figure S71. The dependence of inhibition of factor XIa-induced chromogenic substrate hydrolysis on the concentration of 5d

The dependence of inhibition of factor Xa- and XIa-induced chromogenic substrate hydrolysis on the concentration of 5h

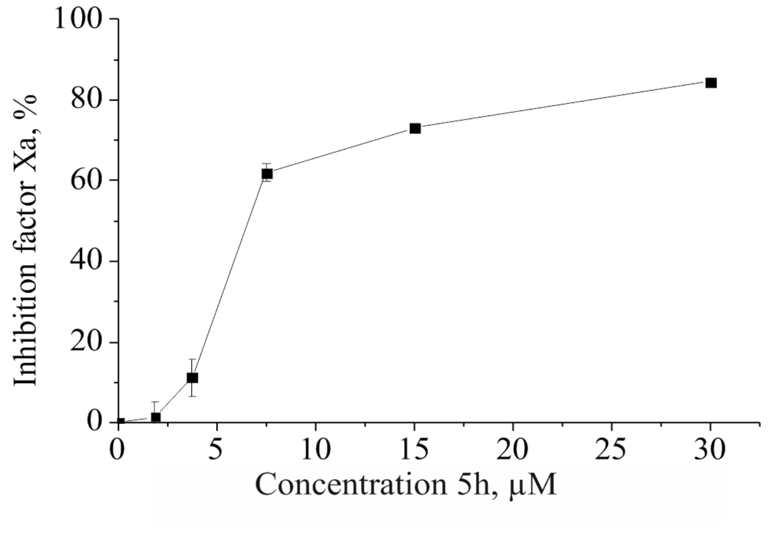

Figure S72. The dependence of inhibition of factor Xa-induced chromogenic substrate hydrolysis on the concentration of 5h

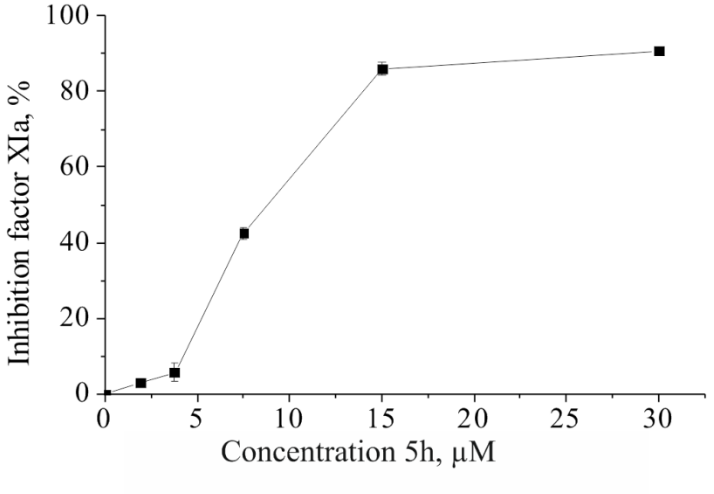

Figure S73. The dependence of inhibition of factor XIa-induced chromogenic substrate hydrolysis on the concentration of 5h

Crystal data and structure refinement parameters for **5d**

	**5d**
Empirical formula	$C_{23}H_{20}ClFN_4OS$
Formula weight	454.94
T, K	100
Crystal system	Monoclinic
Space group	$P2_1/n$
Z	4
a, Å	14.6644(2)
b, Å	9.44790(10)
c, Å	16.2028(2)
α, °	90
β, °	111.423(2)
γ, °	90
V, Å^3	2089.76(5)
D_{calc} (g cm^{-1})	1.446
Linear absorption, μ (cm^{-1})	28.3
F(000)	944
2θmax, °	161.5
Reflections measured	23975

Independent reflections	4567
Observed reflections [I > 2σ(I)]	4381
Parameters	311
R1	0.0451
wR2	0.1171
GOF	1.066
$\Delta\rho_{max}$/ $\Delta\rho_{min}$ (e Å^{-3})	0.466/-0.312
CCDC	2393399

Table S1. Crystal data and structure refinement parameters for 5d
